# Tryptophan Indole Metabolites Reduce Anastomotic Leakage Through Aryl Hydrocarbon Receptor-Driven Interleukin-22 Production

**DOI:** 10.1016/j.jcmgh.2026.101756

**Published:** 2026-02-16

**Authors:** Vasiliki Bantavi, Laura Gloeck, Patrick Leven, Konstantina Zafeiropoulou, Olaf Welting, Hilal Sengül, Patrik Efferz, Bianca Schneiker, Timothy Ryan McCulloch, Christoph Wilhelm, Sandra Blaess, Anneke Fuss, REVEAL study group, Joop L.M. Konsten, Jan Stoot, Nicole D. Bouvy, Joep P.M. Derikx, Bruno Sovran, Wouter J. de Jonge, Sven Wehner

**Affiliations:** 1 Department of General, Visceral, Thoracic, and Vascular Surgery, University Hospital Bonn, University of Bonn, Bonn, Germany; 2 Tytgat Institute for Liver and Intestinal Research, Amsterdam Gastroenterology Endocrinology and Metabolism, Amsterdam University Medical Centers, University of Amsterdam, Amsterdam, The Netherlands; 3 Department of Pediatric Surgery, Emma Children’s Hospital, Amsterdam University Medical Centers, University of Amsterdam, Amsterdam, The Netherlands; 4 Immunopathology Unit, Institute of Clinical Chemistry and Clinical Pharmacology, University Hospital Bonn, University of Bonn, Bonn, Germany; 5 Emma Center for Personalized Medicine, Amsterdam University Medical Center (UMC), University of Amsterdam, Amsterdam, The Netherlands; 6 Neurodevelopmental Genetics, Medical Faculty, Institute of Reconstructive Neurobiology, University of Bonn, Bonn, Germany

**Keywords:** Aryl Hydrocarbon Receptor, Interleukin-22, Intestinal Anastomotic Leakage, Tryptophan Metabolism

## Abstract

**Background & Aims:**

Colorectal cancer (CRC) often requires surgical resection of the tumor and an anastomosis. Anastomotic leakage (AL) occurs in 2.8% to 30% of patients, which increases postoperative morbidity and complications. The preoperative microbiome composition is implicated in AL. Recent studies have shown that microbial tryptophan (Trp) metabolism into aryl hydrocarbon receptor (AhR) indole-derivatives contributes to intestinal tissue healing. Here, we addressed the role of Trp and its metabolites in AL in a CRC patient cohort and a colon-anastomosis mouse model.

**Methods:**

Targeted quantitative metabolomics was performed in preoperative fecal samples from patients with CRC recruited in the REVEAL cohort (n = 388), including 19 AL cases. Anastomotic healing (AH) was tested in a mouse model using wild-type (WT), *AhR*^*−/−*^, *Villin*^*Cre*^*Ahr*^*fl/fl*^, and interleukin (IL)-22 drug-targeting to evaluate the role of AhR and IL-22 in AL.

**Results:**

Fifty-two of the 388 patients with available preoperative fecal samples were matched for AH/AL occurrence (AL, n = 19; AH, n = 33). Among Trp metabolites, indole-3-acetic acid was significantly reduced in AL compared with matched AH male patients. *AhR*^*−/−*^ mice displayed more severe AL, reduced IL-22 expression, and a marked loss of IL-22-expressing type 3 ILCs compared with WT mice. Neutralizing IL-22 antibody augmented AL in WT mice, whereas IL-22Fc application ameliorated AL in *AhR*^*−/−*^ mice. AhR agonism failed to rescue healing under IL-22 deficiency. Furthermore, low-Trp diet-fed WT mice exhibited reduced fecal concentration of AhR agonists, AhR-agonist producing bacteria, and augmented AL. This phenotype was prevented by dietary supplementation with the AhR agonist indole-3-carbinol.

**Conclusions:**

Stimulation of the AhR/IL-22 by synthetic agonists or dietary-derived Trp-metabolites can prevent AL.

## Graphical abstract

**Figure:**
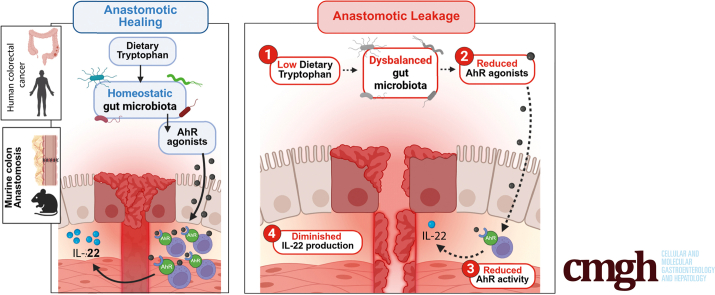

**Summary**

Male patients with anastomotic leakage showed reduced preoperative fecal levels of indole-3 acetic acid. Aryl hydrocarbon receptor promotes anastomotic healing via the interleuken-22 signaling pathway. Aryl hydrocarbon receptor agonists rescue low tryptophan diet-induced healing disturbances in mice.

**What You Need to Know**

**Background**

Preoperative microbiome composition and tryptophan-derived indole metabolites that activate aryl hydrocarbon receptors (AhRs) influence intestinal healing; we examined their role in anastomotic leakage in a colorectal cancer patient cohort and a colon-anastomosis mouse model.

**Impact**

AhR activation through interleukin-22 enhances anastomotic healing, suggesting that stimulating this pathway could reduce the risk of anastomotic leakage. Both synthetic agonists and dietary tryptophan metabolites show therapeutic potential.

**Future Directions**

Future studies should focus on optimizing AhR/interleukin-22-based therapies for anastomotic leakage prevention, exploring the role of diet and AhR agonists in clinical settings, and evaluating postoperative outcomes in patients.

Anastomotic leakage (AL) is a detrimental complication after colorectal surgery, wherein the anastomosis heals incompletely, leading to infections and disturbed gut barrier function.^1^, ^2^, ^3^ It can occur in up to 30% of patients after intestinal resection of the colon or rectum and is more prevalent in distal colorectal and coloanal anastomosis.^4^ AL can lead to complications ranging from small abscess formation to peritonitis, sepsis, and even death if left untreated or treatment is delayed.^5^ It is associated with higher mortality and significant morbidity rates, which result in prolonged hospitalization and increased health care costs.^6^ The etiopathogenesis of AL is not entirely known, and it is recognized as a multifactorial disease where host genetics, gut microbiota, and perioperative inflammation all contribute to risk of leakage.^1^

Disturbance in the gut microbiota composition assumed to play a role in AL as in many chronic inflammatory conditions, such as celiac disease, inflammatory bowel diseases (IBDs), and post-infectious irritable bowel syndrome.^7^, ^8^, ^9^, ^10^, ^11^ Evidence of the impact of gut microbiota on AL was first demonstrated by Shogan et al, showing that postoperative accumulation of *Enterococcus faecalis*, a collagenolytic bacterium, impairs anastomotic healing (AH) and triggers AL.^12^ More recently, Hajjar et al associated specific preoperative microbiota with the pathogenesis of AL in patients undergoing colorectal cancer (CRC) surgery.^13^

Microbial metabolites of tryptophan (Trp), including kynurenine, serotonin, and indole or its derivatives, act as agonists of the aryl hydrocarbon receptor (AhR).^14^^,^^15^ It is known that AhR activation regulates the production of certain cytokines, such as interleukin (IL)-10, IL-17, and IL-22, in immune cells, which all play pivotal roles in mucosal healing.^11^^,^^16^^,^^17^ Interestingly, inflammatory lesions were associated with altered Trp metabolism, leading to reduced levels of AhR agonists and its downstream target, IL-22.^14^^,^^18^^,^^19^

In the current study, we investigated the protective role of indoles against AL in a patient cohort and a mouse model. We particularly described a critical role of indole formation in activating the AhR-driven IL-22 production to support AH.

## Results

### Reduced Levels of Fecal Indole-3-acetic Acid Associate With Anastomotic Leakage in Patients With CRC

**Figure 1:**
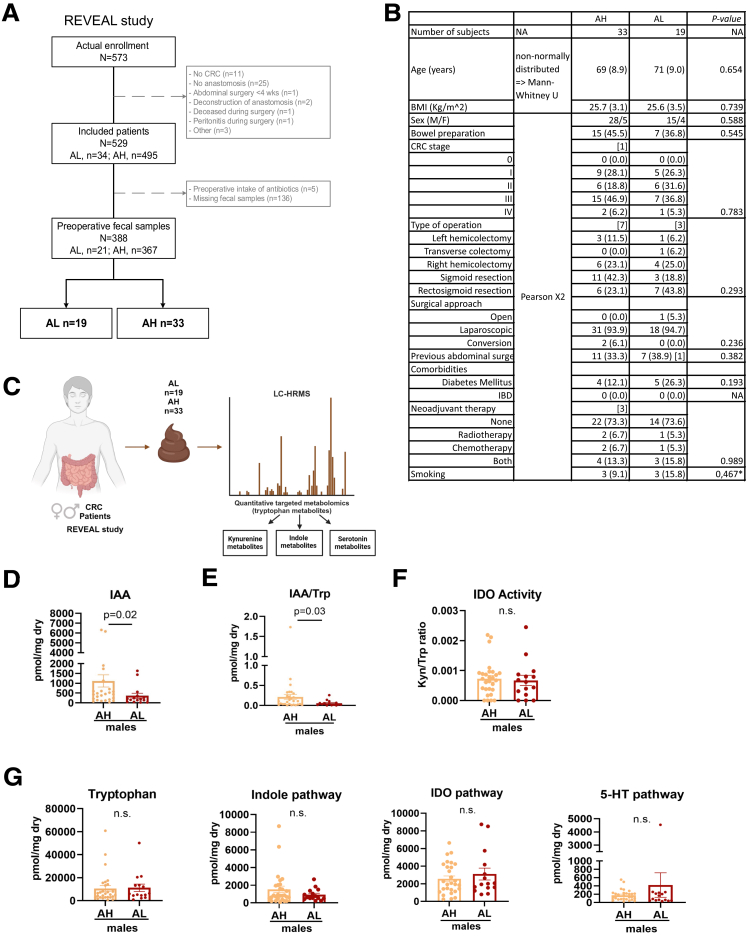
**REVEAL study.** (*A*) Scheme of the REVEAL study for Trp metabolite analysis. (*B*) Flow chart of the REVEAL study. (*C*) Patient characteristics. (*D*) Concentration of IAA in the preoperative stool samples of male patients (AH: n = 28; AL: n = 15) that underwent CRC surgery. (*E*) IAA/Trp ratio in males. (*F*) IDO activity (calculated as the kynurenine-to-Trp ratio) in male patients. (*G*) Quantification of Trp and total metabolites from the indole, kynurenine (IDO) and serotonin (5-HT) pathway in stool of male patients. Each dot represents a patient, with bars showing mean ± SEM. Statistical significance was determined by Mann-Whitney *U* test or Pearson χ^2^. Numerical variables resemble the median. Categorical variables: n (% within group), percentages have been calculated in the available data. Square brackets indicate missing data. ∗Previous smokers are included in the ‘’No’’ group. ∗*P* < .05.

To address the role of gut luminal AhR agonists in patients with AL, we analyzed the concentrations of Trp metabolites in preoperative stool samples from patients of the REVEAL study (Figure 1*A*).^20^ Preoperative stool samples could be collected from 388 of 529 included patients, of whom 21 developed AL, whereas 367 presented with normal AH (Figure 1*B*). Trp metabolomics was performed on 19 patients with AL (15 males, 4 females) and 33 patients with AH (28 males, 5 females) with similar clinical characteristics (Figure 1*C* and Supplementary Table 1). Analysis showed a trend towards higher levels of indole-3-acetic acid (IAA), a known AhR agonist^14^^,^^16^ in patients with AH vs AL (*P* = .06) (Supplementary Table 2), but no difference in the other metabolites related to the indole, kynurenine (indoleamine 2,3-dioxygenase [IDO]), or serotonin (5-hydroxytryptamine [5-HT]) pathway or in Trp itself (Supplementary Table 2) between patients with AL and AH. The overall IDO activity, determined by the kynurenine-to-Trp ratio, also remained unchanged between the 2 groups (Supplementary Table 2). Given that male sex is a risk factor for AL, we then compared Trp metabolite levels within the male patients and observed that the IAA and indole-3-lactic acid (ILA) concentrations were significantly higher in the stool of healing male patients compared with patients with AL (*P* = .02 and *P* = .04, respectively) (Figure 1*D*, Supplementary Table 3). Supportingly, the IAA-to-Trp ratio was higher in male patients with AH compared with male patients with AL (*P* = .03), suggesting that Trp is metabolized towards the indole pathway (Figure 1*E*). No difference in the IDO activity (Figure 1*F*), Trp itself, or the other metabolites related to the indole, kynurenine (IDO), or serotonin (5-HT) pathway was observed (Figure 1*G*, Supplementary Table 3). Analysis of female patients showed no difference in the metabolites between AH and AL (Supplementary Table 4).

### AhR Deficiency Leads to Enhanced Rates of AL in a Mouse Model of Colon Anastomosis

**Figure 2:**
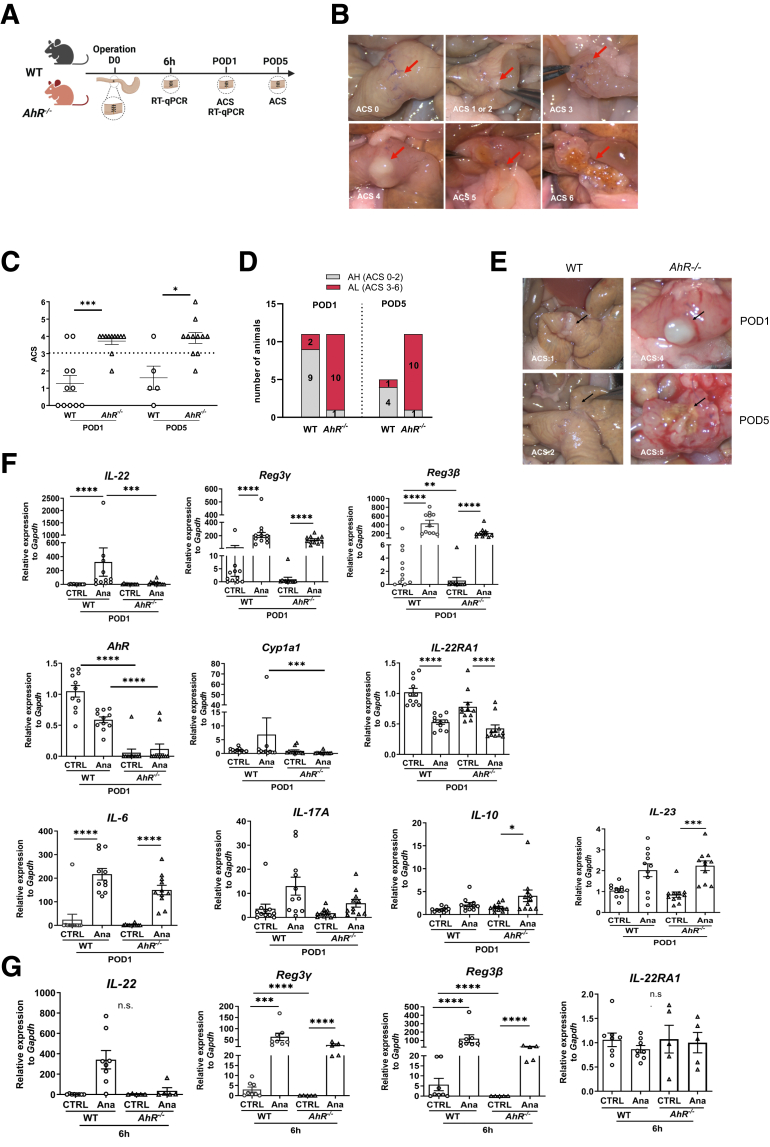
**AhR deficient mice are sensitive to colonic AL.** (*A*) Scheme of the surgical method and timeline. (*B*) Representative images for each score. (*C*) ACS in WT and *AhR*^*−/−*^ mice showed a difference between the 2 groups on POD1 (WT: n = 11; *AhR*^*−/−*^: n = 11) and on POD5 (WT: n = 5; *AhR*^*−/−*^: n = 11). (*D*) *AhR*^*−/−*^ mice showed more frequent AL (ACS ≥3) compared with WT mice on POD1, and on POD5. (*E*) Representative in situ images of WT and *AhR*^*−/−*^ mice on POD1 and POD5. (*F* and *G*) Gene expression of AhR-related genes in anastomotic (Ana) and naïve colonic tissues (CTRL) collected on POD1 (*E*; *AhR*^*−/−*^: n = 11; WT: n = 11) and 6 hours postoperatively (*F*; *AhR*^*−/−*^: n = 5; WT: n = 8). Each dot represents 1 mouse, with bars showing mean ± SEM. *Arrows* indicate the anastomosis and the complications. Statistical significance was determined by Mann-Whitney *U* test (*C*; comparison of groups per timepoint) or 2-way ANOVA with Tukey’s post hoc test (*G* and *H*). ∗*P* < .05; ∗∗*P* < .01; ∗∗∗*P* < .001; ∗∗∗∗*P* < .0001.

Given that IAA is a known AhR agonist,^16^ we evaluated the role of AhR in AH/AL in AhR-deficient (*AhR*^*−/−*^) and wild-type (WT) mice at different time points after an end-to-end proximal colon anastomosis (Figure 2*A*). Anastomosis was assessed on postoperative day (POD) 1 and 5 using an anastomotic complication score (ACS),^21^ with ACS ≥3 representing AL (Figure 2*B*, Supplementary Table 5). On POD1, AL rates increased in *AhR*^*−/−*^ (90.9% ACS ≥3) compared with WT mice (18.2% ACS ≥3; *P* < .001) (Figure 2*C–E*). On POD5, ACS scores were still elevated in *AhR*^*−/−*^ mice compared with WT mice (*P* < .05), with 2 of 11 AL mice presenting the highest AL levels (ACS = 5–6), characterized by severe abscess formation, spread of pus, and signs of fecal peritonitis, (Figure 2*C–E*).

Next, we analyzed the expression of AhR and its downstream target genes, including immune-related and host-defense genes. Notably, these analyses were initially performed on POD1, as the immune response is most prominent within the first day after surgery (Figure 2*F*). *AhR* expression was detected in WT but not in *AhR*^*−/−*^ mice (Figure 2*F*). Interestingly, we found a notable increase (144-fold; *P* < .0001) in expression of *IL-22*, a cytokine acting on epithelial cells^22^^,^^23^ and supporting barrier function in the anastomotic tissue of WT mice. Notably, *IL-22* expression remained significantly low in the anastomotic tissue of *AhR*^*−/−*^ mice (Figure 2*F*). We also investigated IL-22 expression as well as the expression of some of the canonical *IL-22* regulated genes, namely *Reg3b, Reg3g*, and *IL-22RA1*, one of the *IL-22* receptors expressed by epithelial cells, at both 6 hours and on POD1 tissues (Figure 2*F* and *G*). At 6 hours, the expression of *IL-22* was higher than at POD1 in WT mice (151-fold; *P* = .06) (Figure 2*G*). The expression of *Reg3b* and *Reg3g* was significantly altered between naive colon (control [CTRL]) of WT and *AhR*^*−/−*^ mice at 6 hours and only for *Reg3b* on POD1, although we observed no difference in *IL-22RA1* expression between WT and *AhR*^*−/−*^ at either time point (Figure 2*F* and *G*). Additionally, we investigated the expression of other pro- and anti-inflammatory cytokines, such as *IL-17A*, *IL-23*, *IL-10*, and *IL-6,* previously linked to the AhR pathway (Figure 2*F*). Levels of *IL-6* were significantly higher in the anastomotic tissue in both WT and *AhR*^*−/−*^ mice compared with their respective naïve tissues, but unlike *IL-22,* there was no significant difference between WT and *AhR*^*−/−*^ mice (Figure 2*F*). *IL-17A* showed a trend of downregulation in the anastomotic tissue of *AhR*^*−/−*^ compared to WT mice (Figure 2*F*), whereas *IL-10* and *IL-23* showed no difference between genotypes (Figure 2*F*) and a slight increase in *AhR*^*−/−*^ anastomosis compared to naïve controls. Together, AhR-driven *IL-22* gene expression is strongly induced during anastomosis.

### AhR-Deficient Mice Display Diminished Numbers of IL-22-Producing ILC3s

**Figure 3:**
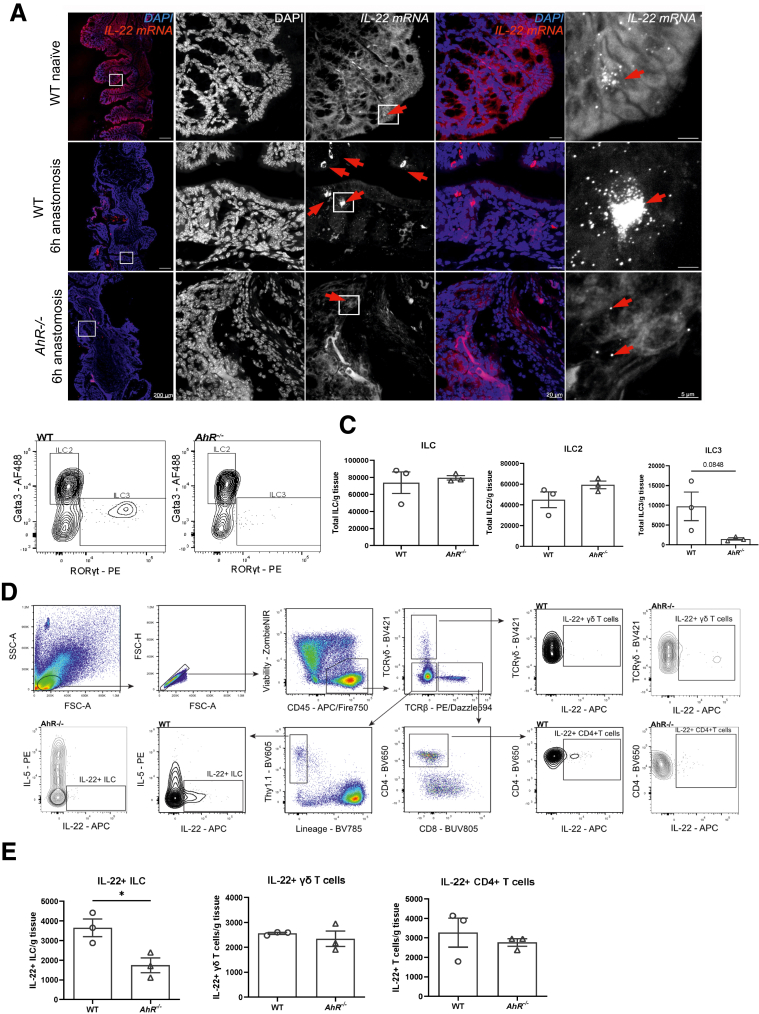
**Detection of IL-22 mRNA and IL-22 producing cells.** (*A*) Representative RNAscope fluorescent in situ hybridization of *IL-22* mRNA in naïve WT mice, and WT and AhR^−/−^ mice 6 hours after anastomotic surgery. Nuclei were stained with DAPI (*blue*); *IL-22* mRNA signals are shown in *white or red* (in the merged images). Regions of interest (ROIs) with higher magnification are indicated by *white rectangles*. Scale bar from left to right: 200 μm, 20 μm, 5 μm. n = 2 mice per group. *Arrows* indicate *IL-22* mRNA. (*B*) Representative flow cytometry plots showing Gata3 (ILC2 marker) and RORγt (ILC3 marker) expression in anastomotic tissue of either WT or *AhR*^*−/−*^ mice 12 hours after surgery. (*C*) Total ILC, ILC2, and ILC3 cell numbers per gram of anastomotic tissue. (*D*) Gating strategy used to identify IL-22 producing cells in anastomotic tissue of WT mice 12 hours post-surgery and after an additional 6 hours of IL-1β/IL-23 stimulation. Additional representative plots of IL-22 and either TCRγδ (γδ T cells), IL-5 (ILC), or CD4 (CD4^+^ T cells) are shown for both WT and *AhR*^*−/−*^ mice. (*E*) Numbers of IL-22-producing ILCs, γδ T cells and CD4^+^ T cells per gram of anastomotic tissue. (*B–E*) n = 3 mice per group. Bars showing mean ± SEM. Statistical significance was determined by Student’s 2-tailed *t* test. ∗*P* < .05.

As *IL-22*, as well as its downstream targets *Reg3b* and *Reg3g*, were significantly reduced in anastomotic tissue of *AhR*^*−/−*^ mice compared with WT mice, we wanted to investigate its cellular origin. Using RNAscope, we were able to visualize in situ IL-22 mRNA expression in naïve WT tissue and 6 hours after anastomosis surgery, the time point when anastomotic *IL-22* mRNA expression was highest (Figure 3*A*). Interestingly, *IL-22* mRNA expression appeared tightly clustered in the anastomotic region of WT mice, whereas it was absent in both naïve WT and *AhR*^*−/−*^ mice. Additionally, *AhR*^*−/−*^ mice displayed a strongly reduced *IL-22* mRNA signal in anastomotic tissue (Figure 3*A*). Next, we investigated the cellular origin of IL-22 by flow cytometry. As the AhR is important for the development of type 3 innate leukocyte cells (ILCs),^24^ we first characterized both ILC2 and ILC3 populations in both WT and *AhR*^*−/−*^ mice. Although we observed no difference in overall total ILC numbers, total ILC2s were increased, whereas ILC3s were reduced in *AhR*^*−/−*^ mice (*P* = .0848) (Figure 3*B* and *C*). IL-22 is known to be produced by multiple immune cells, including various types of lymphoid cells, but not epithelial cells.^25^ Analysis of cells isolated from the anastomosis for their capacity to produce IL-22 revealed ILCs, γδ T cells, and CD4^+^ T cells as cellular origin in both WT and *AhR*^*−/−*^ mice. However, only IL-22^+^ ILCs were significantly reduced in AhR^−/−^ compared with WT mice (Figure 3*D* and *E*). These findings highlight the importance of AhR signaling and ILCs in AL, indicating that a reduction of ILC3s subsequently induces IL-22 deficiency, which might be one of the molecular pathways behind AL.

### AhR-Driven IL-22 Supports Anastomotic Healing

**Figure 4:**
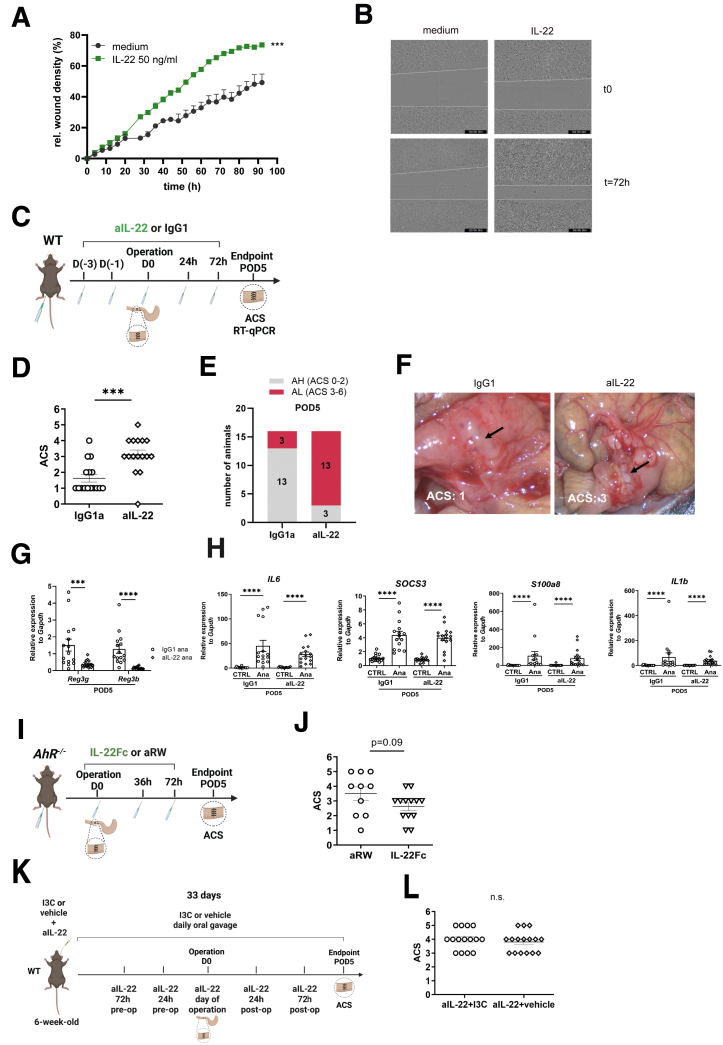
**AhR-triggered IL-22 release supports AH.** (*A* and *B*) Scratch test on HT-29 treated with human recombinant IL-22: quantification of the relative wound density and representative images. (*C*) Scheme of the anastomotic surgery experiment in WT mice receiving pre- and postoperative intraperitoneal injections of neutralizing aIL-22 antibody (n = 16) or IgG1a control (n = 16). (*D*) ACS from anastomotic surgery on POD5 corresponding to (*C*). (*E*) aIL-22-treated WT mice showed more frequent AL (ACS ≥3; 13 of 16 mice) compared with control IgG1a-treated WT mice (3 of 16) on POD5. (*F*) Representative images from IgG1a and aIL-22-treated mice. (*G*) Transcript expression of *Reg3g* and *Reg3b*, 2 downstream targets of IL-22 in the anastomotic tissue of IgG1 and aIL-22-treated WT mice. (*H*) Transcript expression of *IL-6*, *IL-1β*, *S100a8*, and *Socs3* in the anastomotic and non-anastomotic control tissue of IgG1 and aIL-22-treated WT mice. (*I*) Scheme of the anastomotic surgery in *AhR*^*−/−*^ mice that received postoperative intraperitoneal injections of recombinant IL-22Fc protein (n = 13) or aRW control (n = 10). (*J*) ACS from anastomotic surgery on POD5 corresponding to (*I*). (*K*) Scheme of the anastomotic surgery experiment in WT mice treated with aIL-22 antibody and I3C (n = 16) or vehicle control (n = 16). (*L*) ACS from anastomotic surgery on POD5 corresponding to (*K*). Data represents pooled results of 2 independent experiments. Each dot represents a mouse, with bars showing mean ± SEM. Statistical significance was determined by unpaired *t*-test (*A*, *G* and *J*), Mann-Whitney *U* test (*D* and *L*), or 2-way ANOVA with Tukey’s post hoc test (*H*). ∗*P* < .05; ∗∗∗*P* < .001; ∗∗∗∗*P* < .0001. aIL-22, antil-IL-22.

Consequently, we studied the role of IL-22 in healing and AL. Treatment with recombinant IL-22 induced faster wound closure in an epithelial scratch assay (Figure 4*A* and *B*). To study the potential supportive role of IL-22 in vivo, we first treated WT mice with an anti-IL-22 neutralizing antibody (aIL-22 nAb) (Figure 4*C*). The aIL-22 treatment strongly increased AL rates (Figure 4*D–F*), underlining the essential role of IL-22 in transmural wound healing. In addition, aIL-22 nAb treatment revealed a significant reduction of *Reg3b* and *Reg3g*, 2 downstream target genes of IL-22 (Figure 4*G*). We also investigated the expression of *IL-6, IL-1β, S100a8,* and *Socs3*, but did not observe any difference between aIL-22- and IgG1a-treated mice, suggesting a distinct role for IL-22 in its potential to prevent AL (Figure 4*H*).

Given the supportive role of AhR and IL-22 in AH, we investigated whether intraperitoneal application of recombinant IL-22 protein rescues *AhR*^*−/−*^ mice from AL (Figure 4*I*). *AhR*^*−/−*^ mice treated with recombinant IL-22Fc protein displayed reduced AL compared with the anti-ragweed isotype control (aRW)-treated littermates, albeit not significant (*P* = .09) (Figure 4*J*). These data indicate that IL-22 can partially compensate for the absence of AhR, supporting its role as a downstream mediator of AhR-dependent intestinal healing.

### AhR Agonism via Indole-3-Carbinol Depends on IL-22 Signaling for Anastomotic Healing

To determine whether AhR-driven AH requires IL-22 activation, we next examined the effect of blocking IL-22 while activating AhR through the AhR agonist indole-3-carbinol (I3C). WT mice were given either I3C or vehicle by oral gavage, and then they were injected with the aIL-22 antibody (Figure 4*K*; n = 16 per group). One mouse from the I3C-treated group was excluded from the analysis as it was euthanized on POD4 to meet humane endpoint criteria. Both groups exhibited impaired AH, as evidenced by comparable high ACS, with no significant difference between I3C- and vehicle-treated mice (*P* = .5899) (Figure 4*I*). These findings indicate that neutralization of IL-22 profoundly compromises anastomotic healing, and that AhR agonism through I3C exerts its effects on AH predominantly via the IL-22 signaling pathway.

### Epithelial AhR Activation Is Involved in Anastomotic Healing

**Figure 5:**
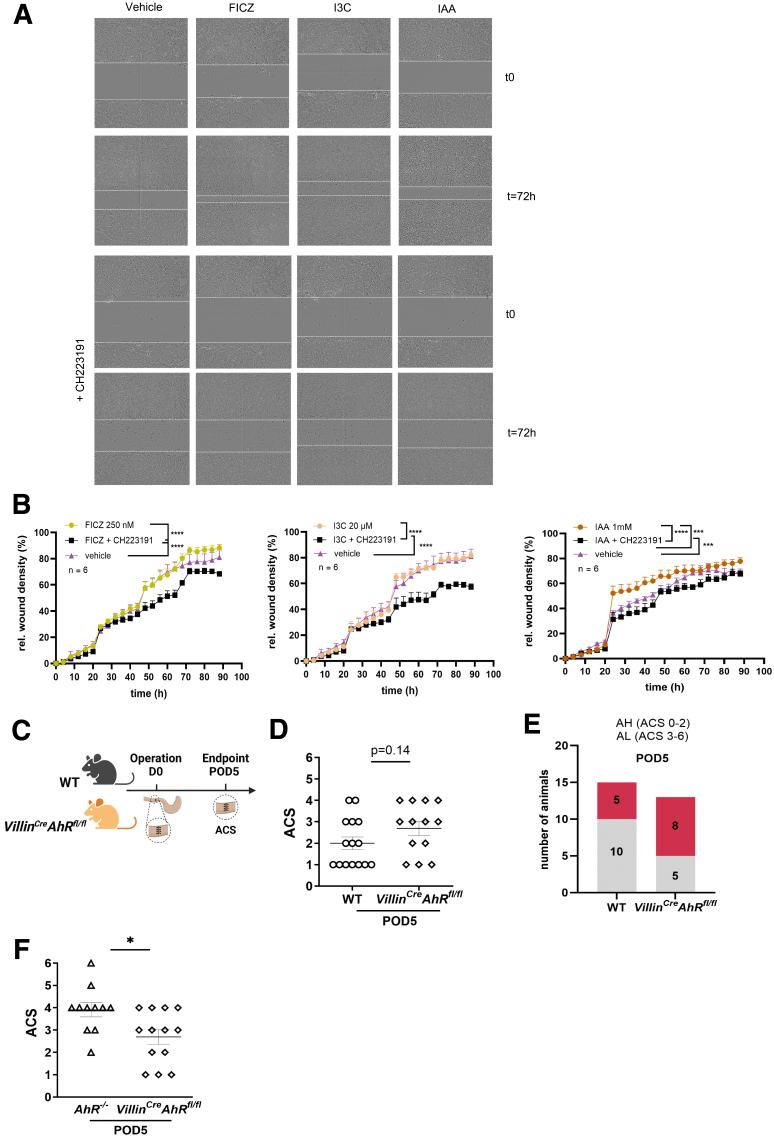
**Epithelial-restricted AhR activation in cell migration and AH.** (*A*) Representative images from a scratch test on HT-29 cells treated with either I3C, IAA, or FICZ (*upper panel*), or one of these substances and AhR antagonist CH223191 (*bottom panel*) at the day of the scratch (t = 0) or three days later (t = 72 hours). (*B*) Quantification of the relative wound density (%) of conditions in (*A*) over the course of 88 hours in 4-hour intervals. Results show mean ± SD (n = 6). (*C*) Scheme of the anastomotic surgery experiment in *Villin*^*Cre*^*AhR*^*fl/fl*^ mice. (*D* and *E*) ACS and frequency of AL occurrence from the anastomotic surgery in *Villin*^*Cre*^*AhR*^*fl/fl*^ mice (n = 13) and their WT littermates (n = 15). (*F*) Comparison of ACS between *Villin*^*Cre*^*AhR*^*fl/fl*^ and full *AhR*^*−/−*^ mice. Each dot represents a mouse, with bars showing mean ± SEM. Statistical significance was determined by 2-way ANOVA with Dunnett’s multiple comparisons test (*B*), and Mann-Whitney *U* test (*D* and *F*). ∗*P* < .05; ∗∗*P* < .01; ∗∗∗∗*P* < .0001.

Because epithelial expression of AhR is known to support intestinal epithelial healing,^26^ we also aimed to elucidate if epithelial AhR activation contributes to AH. First, to prove that AhR is essential for epithelial regeneration, we performed an in vitro experiment. In a scratch assay, carried out with HT29 human intestinal epithelial cells (IECs), treatment with the AhR agonists FICZ (6-formylindolo[3,2b] carbazole), or IAA accelerated wound closure, but not I3C (Figure 5*A* and *B*). The process could be blocked by simultaneous addition of the AhR inhibitor CH223191 in all treatments, demonstrating a role for AhR in epithelial wound healing (Figure 5*A* and *B*).

Next, we assessed whether epithelial AhR-signaling also affects in vivo full thickness bowel wall healing in *Villin*^*Cre*^*AhR*^*fl/fl*^ mice and their Cre-negative littermates subjected to the colon anastomosis model (Figure 5*C*). A comparison of AL severity levels between *Villin*^*Cre*^*AhR*^*fl/fl*^ and full *AhR*^*−/−*^ mice showed that *AhR*^*−/−*^ mice have higher ACS scores than *Villin*^*Cre*^*AhR*^*fl/fl*^ mice (Figure 5*F*). However, this effect was not present when AH was compared between *Villin*^*Cre*^*AhR*^*fl/fl*^ mice and their Cre-littermates, although the latter had slightly but not significantly reduced numbers of animals with ACS score ≥3 compared with their *Villin*^*Cre*^*AhR*^*fl/fl*^ littermate mice (*P* = .14). (Figure 5*D* and *E*). These findings reveal that, although AhR-signaling supports epithelial healing in vitro, it does not play a dominant role in AH in vivo.

### Low Trp Diet Alters Bacterial AhR Agonists Production, Leading to Increased AL

**Figure 6:**
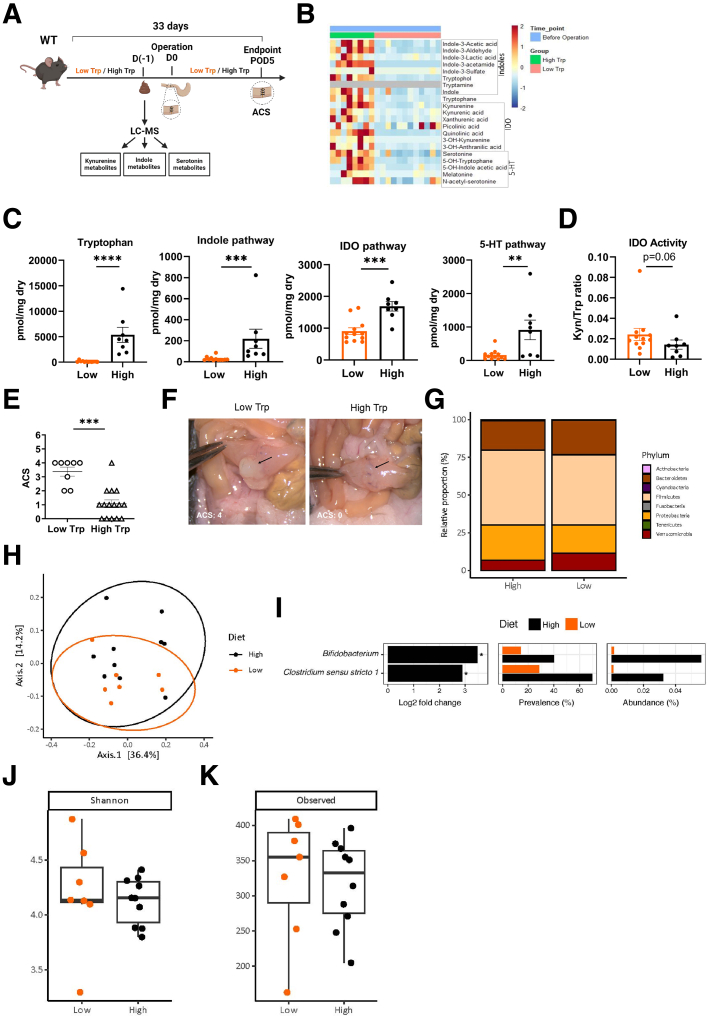
**Low-Trp diet alters bacterial AhR ligand production leading to increased AL.** (*A*) Scheme of Trp diet experiments. (*B*) Heatmap of Trp metabolites in preoperative fecal samples of WT mice fed a low-Trp (n = 12) or high-Trp (n = 8) diet, depicting the feces in the high-Trp group having higher concentrations of all the metabolites that are produced from the 3 Trp metabolic pathways compared with the feces from the low-Trp group, where they are significantly reduced. (*C*) Quantification of Trp and total metabolites from the indole, IDO, and 5-HT pathways. (*D*) IDO activity. (*E*) ACS on POD5 from operated WT mice after low- (n = 8) or high-Trp (n = 15) diets. (*F*) Representative images. (*G*) Mucosal-adherent relative abundance at the phylum level for mice fed the low Trp diet (n = 7) or the high-Trp (n = 10) diet. (*H*) PCoA for bacterial *16S rRNA* gene sequences based on unweighted UniFrac distance in the mucosal swabs of mice fed the low Trp diet (n = 7) or the high-Trp (n = 10) diet. (*I*) Relative abundance of taxa annotated to *Bifidobacterium* and *Clostridium sensu stricto 1*. (*J*) Alpha diversity of mucosal-adherent microbiota of WT mice fed a low-Trp diet (n = 7) or a high-Trp diet (n = 10), shown by the Shannon index and (*K*) the observed richness. Each dot represents a mouse, with bars showing mean ± SEM. Statistical significance was determined by Mann-Whitney *U* test (*C* and *E*: indoles, tryptophan, *D*) or unpaired *t* test (*C*: IDO pathway, 5-HT pathway). For differences in microbial composition, all statistical analyses were performed in R. PERMANOVA was applied using the vegan Adonis function on distance matrices. Differences in genus relative abundances between groups were found using the DESeq method. ∗∗*P* < .01; ∗∗∗*P* < .001; ∗∗∗∗*P* < .0001.

We next speculated that nutritional support, designed to favor AhR agonist formation, could prevent AL. Trp-rich diets in WT and *IDO1*^*−/−*^ or NOD/DQ8 mice^11^ have been shown to favor the growth of *Lactobacillus*, a species metabolizing Trp into AhR agonists, thereby promoting intestinal homeostasis. Therefore, we hypothesized that lack or decrease of dietary Trp would increase AL rates. We fed WT mice with diets containing either low (0.1%) or high (1%) Trp for 28 days prior to surgery and up until POD5 (Figure 6*A*). We first assessed the capacity of low- and high-Trp diets to affect the preoperative fecal Trp metabolites profile. Targeted liquid chromatography-mass spectrometry (LC-MS) Trp metabolomics quantification focused on catabolic Trp derivatives, categorized as metabolites of the kynurenine, serotonin, or indole pathways (Figure 6*B*). As expected, fecal Trp concentration was higher in the high-Trp diet group (Figure 6*C*) and was associated with increased concentration of metabolites from the indole pathway, such as IAA, ILA, Trp, and indole (Figure 6*C*, Supplementary Table 6). Metabolites of the kynurenine (IDO) and serotonin (5-HT) pathways, such as kynurenine, kynurenic acid, xanthurenic acid, quinolinic acid, 3-OH-anthranilic acid, 3-OH-kynurenine and serotonin, 5-OH-tryptophan, 5-OH-indole acetic acid, melatonin, and N-acetyl-serotonin were also higher in the high-Trp diet in the healthy WT mice (Figure 6*C* and *D*, Supplementary Table 6). Finally, the kynurenine-to-Trp ratio, a marker for IDO1 enzyme activity, used as a proxy of inflammation, tended to be lower in the feces of the high-Trp diet-fed mice (*P* = .06) (Figure 6*C*). In consequence to the low Trp diet, we observed strongly impaired AH (Figure 6*E* and *F*), with most animals exhibiting abscess formation (ACS = 4), whereas high Trp diet-fed mice mostly presented with simple adhesions or were not affected at all (ACS 0–2) (Figure 6*E* and *F*).

Given that dietary Trp levels have been shown to affect murine fecal microbiome composition, we were wondering if there are also changes to the mucosal-associated microbiome under low- or high-Trp diets in the postoperative course. 16S rRNA sequencing of mucosal swabs were taken from a distant unaffected colonic part on POD5 to not interfere with the anastomosis itself. The colonic mucosal microbial composition of both low- and high-Trp diet-fed animals, constituted mostly of *Firmicutes*, *Bacteroidetes*, and *Proteobacteria* (Figure 6*G*), with minor differences in their relative proportion to the total biomass (*P*-adjusted > .05 for all taxa at phylum level). However, there was no difference in the mucosal-adherent microbial α-diversity of the mice that received the high-Trp diet compared with the mice fed the low-Trp diet (*P* = .733 and *P* = .494, for Shannon-index and observed richness, respectively) (Figure 6*J* and *K*). The treatment with high- or low-Trp diet could also not explain differences in amplicon sequence variant (ASV) community structure (β-diversity) for the unweighted UniFrac distance (R^2^ = .06; *P* = .405) (Figure 5*H*). Interestingly, within the 17 mucosal swabs, 768 distinct ASVs were found. Of these, 52 ASVs differed significantly between the mice that received a high-Trp diet and the mice fed the low-Trp diet (Supplementary Table 8). At genus level, mice fed the high-Trp diet had higher relative abundance of taxa annotated to *Bifidobacterium* and *Clostridium sensu stricto* 1 than mice treated with low-Trp diet (log2fold change [FC] = 3.5 and 2.9, for *Bifidobacterium* and *Clostridium sensu stricto* 1, respectively; *P*-adjusted = .051, for both taxa (Figure 5*I*). Subsumed, although high- and low-Trp diets only moderately affect overall mucosal microbial richness and diversity, we found distinct changes in AhR-agonist generating genera towards a higher presence under high-Trp diets.

### Supplementation With Indole-3-Carbinol Reverses Low Tryptophan-Induced AL

**Figure 7:**
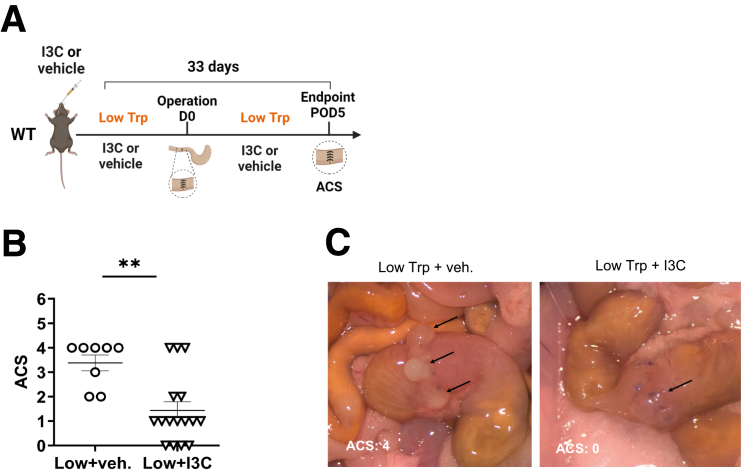
**Supplementation with I3C reverses low Trp-induced AL.** (*A*) Scheme of the I3C/low-Trp diet experiments. WT mice receiving a low-Trp diet were gavaged daily with I3C or vehicle prior- and post-surgery. (*B*) ACS on POD5 from operated WT mice after low-Trp diet and vehicle (n = 8) or I3C (n = 16) supplementation. (*C*) Representative images. Each dot represents a mouse, with bars showing mean ± SEM. Statistical significance was determined by Mann-Whitney *U* test. ∗∗*P* < .01.

To finally validate the implication of low luminal levels of AhR agonists in the low-Trp diet-induced AL, we investigated whether low-Trp diet-fed mice can be rescued from increased AL rates by oral supplementation with I3C (Figure 7*A*). Mice were fed a low-Trp diet and were gavaged daily with I3C, an AhR agonist precursor endogenously metabolized into the active AhR agonists 3,3'-diindolylmethane (DIM) and indolo [3,2-b] carbazole (ICZ). Strikingly, I3C strongly reduced signs of AL, as manifested by the lower ACS (Figure 7*B* and *C*).

Taken together, our findings provide new evidence about the supportive role of the AhR/IL-22 axis in AH and offer potential measures (eg, perioperative IL-22 or dietary Trp supplementation) as supportive measures to prevent AL.

## Discussion

AL is a serious complication following intestinal resection, and its etiology and pathophysiology are still insufficiently characterized. Recent studies addressed the role of the gut microbiome in homeostasis as well as during inflammation and found evidence of a particular role in the latter. Microbiome changes (eg, an increase of bacteria with higher collagenolytic activity^12^^,^^27^^,^^28^) are associated with higher AL rates. Recently, Hajjar et al reported distinct signatures of bacteria related to AL and revealed that this phenotype can be transferred to naïve mice by fecal microbiota transplantation, resulting in enhanced anastomotic inflammation.^13^

The gut microbiome composition depends on diets and, in turn, determines the availability of many bioactive metabolites relevant to intestinal homeostasis and inflammation. The metabolism of the essential amino acid Trp plays a fundamental role in microbiome-host crosstalk. Herein, we provide evidence about a causal role of reduced levels of gut luminal Trp metabolites, particularly AhR-activating indoles, in the pathogenesis of AL in male patients with CRC emphasized by mechanistic insights on the beneficial role of the AhR/IL-22 axis in AH in a murine AH model. First, among a list of 21 Trp metabolites, we associated lower preoperative fecal IAA levels with AL in male patients with CRC but not in the cohort containing males and females. This observation highlights sex as an important factor, coinciding with previous studies listing male sex as a risk factor for AL.^29^

Given that IAA is an AhR agonist, we proved that AhR is essential for proper AH by subjecting pan AhR deficient mice (*AhR*^*−/−*^) displaying signs of AL already on POD1 and even worsening on POD5. Among several tested AhR-dependent target genes, only *IL-22* was strongly induced in anastomotic tissue, especially in the immediate postoperative phase, and fully depends on AhR presence. The early postoperative induction of IL-22 aligns with previous studies, showing that *IL-22* is immediately secreted within the first hours of inflammation.^30^ Moreover, IL-22 prompts colonic mucosal epithelial cells to induce secretion of antimicrobial peptides (AMPs) and other molecules related to host protection,^22^^,^^31^, ^32^, ^33^ findings we could partially confirm within *AhR*^*−/−*^ mice showing a trend towards attenuated *Reg3b* and *Reg3g* gene expression compared with WT mice. Not unexpectedly, AMP expression was not entirely abolished by AhR deficiency, as the release of AMP by Paneth cells and resulting mucosal healing^34^, ^35^, ^36^ was shown to depend on various factors apart from *IL-22*.^22^^,^^36^ Furthermore, multiple groups have demonstrated that AhR acts with IL-22 in maintaining or restoring the epithelial barrier integrity and immunity through various innate immune cell types, such as ILC3, particularly the IL-22 producing NKp46^+^ RORγt^+^ ILC3 cells.^37^^,^^38^ We confirmed the presence of these IL-22^+^ ILCs in the anastomotic tissue of WT mice, which were reduced in *AhR*^*−/−*^ mice.

IL-22 is a highly relevant cytokine due to its ambivalence in inflammation, where it can be protective or pathogenic, depending on the experimental disease model, the involved tissue, and timing (immediate vs delayed).^30^ In the colon of dextran sulfate sodium (DSS)-induced colitis mice, a preclinical model for IBD, mRNA levels of *IL-22* were elevated and seemed protective, as antagonism of IL-22 signaling worsened the disease score and impaired wound recovery.^31^^,^^39^^,^^40^

In agreement with these functions, our findings identify IL-22 as a crucial mediator of intestinal AH. Neutralization of IL-22 in WT mice impaired wound closure and led to AL, confirming that IL-22 signaling is indispensable for proper repair after intestinal surgery. Because IL-22 expression at the anastomotic site depends on AhR signaling, these findings position IL-22 as a downstream target of AhR-mediated regulation in intestinal AH. AhR agonism through IC3 failed to restore healing when IL-22 was neutralized, as both I3C- and vehicle-treated mice exhibited comparable leakage rates. This demonstrates that AhR-induced repair requires intact IL-22 signaling and that AhR acts upstream to regulate IL-22 production. These results suggest that AhR activation alone may be insufficient to drive mucosal healing in conditions where IL-22 signaling is compromised, such as chronic inflammation or microbial dysbiosis.^41^

Conversely, administration of recombinant IL-22Fc protein to *AhR*^*−/−*^ mice only partially improved healing, suggesting a synergistic rather than unidirectional interaction between AhR and IL-22. Further pharmacological studies exploring IL-22Fc dosing, formulations, timing, and application route will be valuable to determine whether exogenous IL-22 can effectively compensate for impaired AhR activity.

It has been shown that AhR is broadly expressed in epithelial, stromal, and immune compartments, highlighting its potential to regulate both immune responses and epithelial regeneration. In our *AhR*^*−/−*^ mice, IL-22 was markedly reduced, largely due to a dramatic decrease in ILC3 cells compared with WT mice. These data indicate that AhR is essential for maintaining IL-22 producing immune cells in our anastomotic model and suggest that impaired IL-22 signaling underlies the anastomotic healing disturbances observed in the *AhR*^*−/−*^ mice.

The AhR is broadly expressed in various intestinal cells.^24^^,^^26^^,^^42^, ^43^, ^44^ Besides its effect in immune cells, which appears to be the main driver of AH, AhR signaling was described being supportive for epithelial healing by a direct action on epithelial cells.^45^^,^^46^ Therefore, we also investigated whether epithelial AhR contributes to AH. ACS scores of *Villin*^*Cre*^*Ahr*^*fl/fl*^ mice did only moderately, but not significantly, differ from their Cre-negative AhR^fl/fl^ littermates. The missing difference indicates that immune cells, particularly ILC3s, are key for the AhR-mediated beneficial action in intestinal “transmural” healing, a process that requires more cells than the epithelium. Nevertheless, our findings do not totally exclude a role of epithelial AhR action in AH, as Metidji et al showed that IL-22 expression is increased in *Villin*^*Cre*^*Ahr*^*fl/fl*^ mice compared with WT mice. This might be due to the elevated levels of AhR agonists that are available to the mucosal immune cells as they were not bound anymore by epithelial AhR.^44^ Therefore, AH in *Villin*^*Cre*^*Ahr*^*fl/fl*^ mice might be partially supported by increased levels of IL-22, and this could explain the modest but non-significant increase of mice with elevated ACS scores in the *Villin*^*Cre*^*Ahr*^*fl/fl*^ group. This explanation is consistent with the concept that epithelial restoration depends on crosstalk between immune-derived cytokines and epithelial responsiveness.^47^

Given the beneficial effect of AhR presence on AH, we studied a nutrition-based AhR agonistic concept as a potentially clinically relevant approach to support AH. Many experimental models have been exploited to test hypotheses regarding dietary-induced shifts in the microbiome. For instance, a high-fat/low-fiber diet, described as a Western diet, creates a dysbiotic microbial community, which increases the AL rate.^48^ Moreover, an enriched Trp diet has displayed decreased immunopathology in nonobese diabetic (NOD) mice expressing the DQ8 celiac disease susceptibility gene (*NOD/DQ8*) after exposure to gluten.^11^ Pre- and perioperative dietary interventions have been a major consideration among surgeons, and one human study has shown an association between higher habitual preoperative dietary fiber intake and lower risk of postoperative complications in patients with CRC.^49^ However, although a healing supportive effect has been suggested, no human clinical trials have been conducted yet.

In line with previous findings reporting increased fecal levels of indole-producing bacteria (eg, *Lactobacillus spp*) and enrichment of AhR agonists after 3-weeks of high-Trp diet feeding to mice,^11^ we detected increased fecal levels of AhR agonists in high-Trp diet-fed WT mice. These agonists included indoles and Trp catabolites generated by the host cells from the IDO and 5-HT pathways, which in the low-Trp diet led to disturbed healing, as shown by the ACS. In contrast to the findings of Lamas et al,^11^ we also observed increased levels of kynurenine. We can speculate that this difference is due to divergent housing conditions in the animal facilities, other genetic backgrounds and microbiomes, or durations of the feeding regimes. However, as kynurenine has been shown to act as an AhR agonist,^50^ its higher presence might promote AhR signaling. As the low-Trp diet promoted AL, strict monitoring and perioperative control of dietary Trp contents in patients scheduled for surgery might present a promising future clinical intervention in the context of AL prevention. Although this study focused on the importance of the microbiome in AH/AL through its production of indoles, it is worth mentioning that intestinal host cells can also metabolize Trp. Different dietary Trp concentration affected the fecal metabolite profile of the mice fecal samples but did not affect significantly the colonic mucosal microbiome. Noteworthy, in a small human crossover study in healthy subjects, oral Trp supplementation showed only modest alterations in fecal microbiome. In particular, the authors reported that supplementation of healthy individuals with oral L-Trp affected duodenal rather than colonic fecal Trp metabolites concentrations as readout through AhR activity assays, leaving the conclusion that Trp is mainly catabolized in duodenum lumen.^51^ Future work should demonstrate these local differences for patients subjected to colonic surgery.

Furthermore, previous studies reported that losing the fragile equilibrium between host cells and gut microbiota leads to various diseases.^14^ Interestingly, the IDO and 5-HT pathways often display higher activation than the indole pathway in multiple intestinal and metabolic diseases.^52^^,^^53^ This pathway shift might either occur due to the impaired ability of the gut microbiota to metabolize Trp into AhR or their direct or indirect (via their effect on the host cells) influence on the other 2 pathways. However, although indoles improve gut and overall human metabolic health, partly by acting as anti-inflammatory molecules via AhR/IL-22 activation,^16^^,^^17^^,^^54^ there are few indole derivatives, such as indole, which is converted into indoxyl sulfate in the liver and is positively correlated with chronic kidney disease.^55^^,^^56^ Therefore, exploiting quantitative methods to determine the concentration of indole and its derivatives in every clinical condition is important for the interpretation of the side effects.

In summary, the present study demonstrates a critical link between AhR and IL-22 signaling pathways, provides new insight into intestinal transmural AH, and suggests potential therapeutic strategies to enhance AH. Supplementation of AhR agonists, recombinant IL-22Fc protein, or preoperative nutritional changes towards diets enriched in Trp seemed to promote physiological AhR activation, which positively affected AH. From a translational perspective, targeting the AhR/IL-22 axis may be particularly relevant for colorectal surgery, where AL remains a major cause of postoperative morbidity and mortality.

### Limitations

This study has few limitations. First, IL-22Fc showed only a trend in improving the impaired AH in *AhR*^*−/−*^ mice. More extensive pharmacological studies are needed to find optimized regimes for improving AH by IL-22 application. Secondly, it remains unclear which cells, apart from epithelial cells, contribute to AhR signaling in AH. However, due to the broad AhR expression in various cell types,^42^, ^43^, ^44^ a concerted action from multiple cell types seems likely. Furthermore, although we set our endpoint on POD5 to enable observation of the healing phenotype, this time point in the wound healing process largely involves tissue remodeling.^57^ Thus, it is defined by the secretion of molecules associated with extracellular remodeling and wound healing, and not cytokines, which are rather expressed in the immediate inflammatory phase of healing.

## Materials and Methods

### Human Study

Patients with colorectal cancer scheduled to undergo surgical resection with anastomosis were recruited in the REVEAL trial, a multicenter, prospective, observational study.^20^ Patients with AL and AH were matched to achieve a ∼1:2 case-control cohort based on age, sex, body mass index (BMI), tumor location, and neoadjuvant therapy. Participants provided written informed consent. The study protocol followed principles of Good Clinical Practice and was conducted in accordance with the Declaration of Helsinki. The study was approved by the Medical Ethical Committee of the Maastricht University Medical Centre (Maastricht, the Netherlands) and registered at ClinicalTrials.gov (ClinicalTrials.gov, Number: NCT02347735). Feces were collected by the patients on the day prior to surgery and immediately stored at the −20°C freezers of the local hospitals. When patients’ recruitment was completed, all samples were transferred to Amsterdam Medical Centers, location AMC, Amsterdam, The Netherlands, and kept for permanent storage at −80°C.

### Animals

Eight-to-ten-week-old male and female *AhR*^*−/−*^ mice and their WT littermates on the C57BL/6JRccHsd background were used. Female mice were purchased either from Janvier-Labs (C57BL/6J) or Envigo (RMS B.V.; C57BL/6JRccHsd) and allowed to acclimatize for 1 week before surgery. Only females were purchased to reduce fights and minimize the number of animals according to the 3R rules. Littermates of the *AhR*^−/−^ and *Villin*^*Cre*^*Ahr*^*fl/fl*^ mice were cohoused after weaning and throughout the experiment. All the mice were maintained in individually ventilated cages under specific pathogen-free (SPF) conditions with standard rodent food, unless otherwise stated in the text, and tap water ad libitum. The mice were randomly re-caged into the different groups in each experiment, and they were randomized within blocks in each experiment. All experiments in Bonn were performed according to the federal law for animal protection and were approved by the State Agency for Nature, Environment and Consumer Protection (LANUV) (#81-02.04.2021.A179). All animal experiments in Amsterdam were approved by the Amsterdam University Medical Center Animal Experiments Committee, and the protocol complied with the Dutch Animal Experimental Act (DMO20-11104-1-11).

### Surgery

Prior to surgery, mice received a subcutaneous injection of analgesic 15 minutes before the operation: tramadol (30 mg/kg body weight; Grünenthal) at the UKB; baytril (5 mg/kg; Bayer) and buprenorphine (0.1 mg/kg; Ecuphar S.A.) at the AMC. The mice underwent general anesthesia with isoflurane (2%–4%). The abdomen was shaved, the skin was disinfected, and a protective salve was placed on the eyes. The mice were placed on a heating pad where they were maintained on pressurized air (2 L/min) at the UKB or oxygen at the AMC and isoflurane (1.5%–2%). After a 2-cm median laparotomy, the cecum and proximal colon were identified, mobilized outside of the abdominal cavity, and placed onto sterile saline infused gauze. The proximal colon (approximately 1 cm from the cecum) was transected, and an end-to-end anastomosis was constructed with 8 interrupted sutures, using 8-0 Vicryl sutures (Ethicon; Cat no. V547G). The integrity of the anastomosis was tested by light pressure of the area with cotton swabs. After creation of the anastomosis, the colon was placed back in the abdominal cavity and warm NaCl was added. The peritoneum was closed with 5-0 Vicryl and the skin sutured with the 5-0 silk (C-0762067). Drinking water was supplemented with tramadol (1 mg/mL; Aliud Pharma) to assist on analgesia (UKB).

### Anastomotic Complication Score

The AH was evaluated using the previously described ACS.^21^^,^^57^^,^^58^ A score between 0 and 2 was considered regular AH, with no signs of AL, whereas a score ≥3 is regarded as AL (Supplementary Table 5). The operations and assessment of anastomosis were performed blindly by the researchers.

### Neutralizing IL-22 Antibody and I3C Administration

For the AhR activation and IL-22 depletion study, 6-week-old female C57BL/6J mice (n = 16) were given daily I3C (100 μL of 25 mg/kg body weight, Sigma-Aldrich, Cat. No. I7256; n = 16), or saline via oral gavage for 3 weeks prior to surgery until sacrifice. The mice were injected with 150 μg/100 μL of anti-IL-22 neutralizing antibody (clone 8E11; Genentech) at the following time points: 72 hours and 24 hours prior to surgery, immediately after surgery, and 24 hours and 72 hours after surgery. All mice were euthanized on POD 5.

### Custom TrP Mouse Diet and I3C treatment

For the diet studies, 5-week-old female C57BL/6J mice (n = 16 per diet) were fed either customized purified diet S0016-E080 (0.1% Trp AA diet) or S0016-E084 (1% Trp AA diet; SSNIFF.de) (Supplementary Table 7), for 3 weeks prior to surgery until sacrifice. In the S0016-E084 group, one mouse died before the operation, and was thus removed from the analysis. An additional set of mice fed with the low tryptophan diet S0016-E080 (0.1% Trp AA diet) and received daily oral gavage of I3C (100 μL of 25 mg/kg body weight, Sigma-Aldrich, Cat. No. I7256; n = 16), or saline (n = 8) for 3 weeks until surgery and subsequent sacrifice.

### IL-22Fc Administration and Neutralizing IL-22 Antibody Administration

Male and female *AhR*^*−/−*^ mice were injected intraperitoneally with 50 μg/100 μL of either IL-22Fc (n = 13) or anti-ragweed isotype control (n = 13) (Genentech) immediately after surgery, 36 hours, and 72 hours after surgery. In the control group, one mouse did not get administered with enough isotype; of the remaining 12, 2 mice died before the end of the experiment, and thus, all 3 were removed from the analysis.

Female C57BL/6J mice (n = 16; 2 independent experiments) were injected with 150 μg/100 μL of either anti-IL-22 nAb (clone 8E11; Genentech) or IgG1 control (Genentech) at the following time points: 72 hours and 24 hours prior to surgery, immediately after surgery, and 24 hours and 72 hours after surgery. All mice were euthanized on POD 5.

### Cell Isolation for Flow Cytometry

Immune cells were isolated from the large intestinal lamina propria. Mice were scarified by cervical dislocation, and the anastomotic affected area was surgically removed. The tissue was cut open and washed in RPMI with 3% fetal bovine serum (FBS) to remove feces, patted dry on a paper towel, and then weighed. The tissue was added to a flask with 10 mL of strip media (RPMI with 3% FBS, 5 mM EDTA, and 1 mM dithiothreitol [DTT]), then incubated for 20 minutes at 37°C on a magnetic stirrer. The tissue was then added to a falcon with 10 mL of shake media (RPMI with 2mM EDTA), then shaken vigorously for 30 seconds. The shake media was replaced, the tissue was shaken 2 more times, and then the tissue was washed with phosphate-buffered saline (PBS). The tissue was then added to a beaker with 1 ml of digest media (RPMI with 0.1 mg/mL Liberase and 0.5 mg/mL DNase) and finely minced with scissors. Another 2 mL of digest media was added and incubated for 27 minutes at 37°C on a magnetic stirrer. The digestion was stopped with 6 mL of RPMI with 3% FBS and digested tissue passed through a 70-μm filter into falcon tubes to generate single-cell suspensions. Tubes were centrifuged at 400 × g for 5 minutes, and supernatant discarded. Cell pellets were resuspended in RPMI with 3% FBS then passed through a 40-μm cell strainer. Single-cell suspensions were centrifuged again, then resuspended in RPMI 10% until further flow cytometry analysis.

### Flow Cytometry

Single-cell suspensions were generated from the lamina propria of anastomotic tissue as described above. For transcription factor staining, dead cells were stained with Zombie NIR (Biolegend). Fc receptors were blocked with anti CD16/CD32 (*InVivo*MAb, BioXCell) before staining of surface antigens for 45 minutes at room temperature. Cells were fixed and permeabilized using a FoxP3/Transcription Factor Staining Buffer Set (eBioscience), then stained with intracellular antibodies for 1 hour at room temperature.

For cytokine staining, cells were incubated for 6 hours in Iscove’s Modified Dulbecco’s Medium (IMDM) with 10% FBS, 10 ng/mL IL-23, and 10 ng/mL IL-1β. Dead cells were stained with Zombie NIR (Biolegend), and Fc receptors blocked with anti CD16/CD32. Cells were stained for surface antigens for 45 minutes at room temperature, fixed in 2% formaldehyde for 20 minutes, then permeabilized with 0.01% NP-40 for 4 minutes. After washing, intracellular antigens were stained for 1 hour at room temperature. Data was acquired on a Sony ID7000 (Sony Biotechnology) and analysis performed using FlowJo software (v10.9, Treestar).

### Fluorescent in Situ Hybridization (RNAscope)

RNAscope Multiplex Fluorescent v2 Assay (ACD, Bio-Techne) was performed according to the manufacturer’s instructions with minor modifications. Cryosectioned tissue sections (12 μm) were briefly air-dried at room temperature and then dried further at 40°C. They were then post-fixed in 4% paraformaldehyde (PFA), dehydrated using an ascending ethanol series (50%, 70%, 100%) and air-dried. Sections were then treated with hydrogen peroxide (hydrogen peroxide solution), subjected to target retrieval (RNAscope Target Retrieval Reagent [10×]) at 100°C, and digested with Protease III for 15 minutes at 40°C. Probes were hybridized for 2 hours at 40°C in a HybEZ oven (ACD, Bio-Techne). After stringent washing steps Wash buffer (RNAscope Wash Buffer), amplification steps (AMP1–3) were performed sequentially at 40°C. Channel-specific horseradish peroxidase (HRP)-based signal amplification was carried out using RNAscope Multiplex FL v2 HRP reagents, followed by tyramide signal amplification (TSA) with Cy5 fluorophore (TSA Vivid dyes, 1:3000 in TSA buffer), and HRP blocking. Nuclei were counterstained with 4′,6-diamidino-2-phenylindole (DAPI) for 1 to 2 minutes. Unless stated otherwise, all reagents for the RNAscope assay were obtained from ACD Bio-Techne. Slides were coverslipped with PolyMount (Polysciences) and stored at 4°C in the dark until imaging. Spinning-disk confocal microscopy (Visitron Visiscope, Visitrom Systems) was performed using standard excitation and emission settings for Cy5 and DAPI. Images were processed with VisiView (Version 6.0.0.37, Visitrom Systems) and Fiji (ImageJ2 - Verion: 2.14.0/1.54g).

### Scratch Wound Assay

IncuCyte live cell imaging system (Sartorius) was used to capture HT29 cell proliferation and migration after wounding, in phase contrast every 4 hours at 4× magnification. The cells were seeded in an IncuCyte Imagelock 96-well plate (Sartorius) at a density of 5 × 10^4^ cells per well in 100 μL of Dulbecco’s Modified Eagle’s Medium (DMEM) high glucose (4.5 g/L) (Capricorn, Cat. No. DMEM-HPXA). After 2 days, the IncuCyte 96-well WoundMaker was used to create a uniform scratch in every well. Stimulation was performed with I3C (20 μM; Sigma-Aldrich, Cat. No. I7256), FICZ (250 ng/mL; Sigma-Aldrich, Cat. No. SML1489-1MG), IAA (100 μM; Sigma-Aldrich, Cat. No. I2886-5G) with or without CH-223191 (10 μΜ; Sigma-Aldrich, Cat. No. C8124-5MG) and recombinant human IL-22 (50 ng/mL Peprotech, Cat. No. 200-22-2UG) for 88 hours and measured on a real-time imaging system (IncuCyte). DMEM/10% FBS/1% penicillin/streptomycin (P/S), the I3C and IL-22 vehicle, and dimethyl sulfoxide (DMSO), the FICZ vehicle, were used as the negative controls. The relative wound density (%), which measures the spatial cell density in the scratched area relative to the cell density outside of the area at every time point, was calculated using the Scratch Wound analysis of the IncuCyteZOOM software (Sartorius).^59^

### Quantification of Fecal Trp Metabolites by Liquid Chromatography Coupled With High-Resolution Mass Spectrometry

Trp metabolite concentrations in the available preoperative fecal samples from the mouse diet study (high Trp group; n = 8, low Trp group; n = 12), and the human preoperative stool samples from the REVEAL study (AH; n = 33, AL; n = 19) were determined by liquid chromatography coupled with high-resolution mass spectrometry (LC-HRMS) as previously described.^60^ IDO activity was determined by the kynurenine-to-Trp ratio.^61^

### RNA Isolation

Total RNA was isolated from colon samples using the RNeasy mini kit (Qiagen; Cat. no. 74106) according to the manufacturer’s instructions. The tissues were homogenized in a Precellys apparatus (30 seconds, 6100 rpm; Bertin Technologies) and kept on ice throughout the extraction process. RNA concentrations were measured in NanoDrop 1000 spectrophotometer (Thermo Scientific).

### Gene Expression Analysis Using Quantitative Real-time Polymerase Chain Reaction

Complementary DNA (cDNA) was synthesized from 1 μg RNA using the High-Capacity cDNA Reverse Transcription Kit (Applied Biosystems; Cat. no. 4368813). Gene expression was quantified by quantitative reverse transcription-polymerase chain reaction (RT-qPCR) performed on a QuantStudio 5 System (Thermo Scientific), using Power SYBR Green PCR Master Mix (Thermo Scientific; Cat. no. #4367659) with the primers listed in Supplementary Table 9. Messenger RNA (mRNA) expression was determined with the 2^−ΔΔCT^ method normalizing to mouse *Gaphd* gene expression and using WT naïve colon group as a calibrator.

### DNA Extraction

DNA was extracted from mucosal-adherent swabs, which were stored in collection and preservation medium (COPAN; Cat No 201CS01), using a combination of repeated bead-beating and the Maxwell RSC Blood DNA kit (Promega Leiden, the Netherlands) with Stool Transport and Recovery (STAR) buffer (Roche). The samples were thawed at room temperature and centrifuged for 10 minutes at 14,000 rpm (4°C). The supernatant was removed, and 100 μL of the precipitant was mixed with 300 μL STAR buffer and 30 μL proteinase K. The total volume, including the swab stick, was transferred to bead tubes, shaken for 20 minutes at 1000 rpm (56°C), and homogenized with the bead beater for 1 minute at 5 m/sec. After 5 minutes of incubation at 95°C, the samples were centrifuged for 5 minutes at 12,700 rpm (4°C), and the supernatant was transferred to a new nuclease-free tube. The latter steps were repeated; 200 μL STAR buffer was added in the bead tubes, homogenized for 1 minute at 5 m/sec, centrifuged for 5 minutes at 12,700 rpm (4°C), and transferred to the respective nuclease-free tube. From the final volume, 250 μL was transferred to the Maxwell tubes, filled with 60 μL of elution buffer (ie, nuclease-free water).

### 16S rRNA Gene Sequencing

16S rRNA gene amplicons were generated using a single-step PCR protocol targeting the V3–V4 region.^62^ The PCR reaction in 20 μL was performed with following thermocycling conditions: initial denaturation at 94°C followed by 25 cycles denaturation (45 seconds at 94°C), annealing (60 seconds at 52°C), extension (90 seconds at 72°C), and final extension at 72°C for 10 minutes. Forward and reverse reads were truncated to 240 and 210 bases, respectively, and merged using USEARCH; merged reads that did not pass the Illumina chastity filter, had an expected error rate higher than 2, or were shorter than 380 bases were filtered. ASVs were inferred for each sample individually with a minimum abundance of 4 reads. Unfiltered reads were then mapped against the collective ASV set to determine the abundances. Taxonomy was assigned using SILVA 16S ribosomal database V132.^63^

### Statistical Analysis

GraphPad Prism version 9.5.1 was used for all analyses and preparation of graphs. SPSS version 28.0.1.1 was used for the patient cohort. Results are expressed as mean ± standard error of the mean (SEM). For comparisons between 2 groups, a 2-tailed Student's *t*-test for unpaired data or a nonparametric Mann-Whitney test was used. Two-way analysis of variance (ANOVA) and post hoc Tukey test were used for comparisons between 4 groups, and analyses were adjusted for multiple comparisons. Pearson χ^2^ was used for the patient characteristics. Normal distribution was determined by the D’Agustino Pearson omnibus normality test, Shapiro-Wilk test, and Kolmogorov-Smirnov test with Dallal-Wilkinson-Lillie correction. For datasets that failed normality tests, a nonparametric Mann-Whitney test was used to analyze statistical differences. Animals were randomly assigned to experimental groups. The sample size for the mice experiments was calculated using G∗Power based on α error probability of .05 and power (1-β error probability) of 0.80. For the 16S rRNA sequencing experiment, all statistical analyses were performed with R (v. 4.3.2, RStudio v. 2023.12.1+402), using the phyloseq (v. 1.46.0), vegan (v. 2.6.4) and DESeq2 (v. 1.42.1) packages. Alpha diversity was examined at observed species richness, and Shannon index level and microbial composition (β-diversity) was assessed using principal coordinate analysis (PCoA) at ASV level based on unweighted UniFrac distance. Non-parametric permutational multivariate analysis of variance (PERMANOVA) was applied using the vegan Adonis function on distance matrices. Differences in ASV and genus relative abundances between groups were found using the DESeq method. For all statistical tests, differences corresponding to *P* < .05 were considered significant. For genera exhibiting significant differences in relative abundance between groups, prevalence was determined as the proportion of samples in which each taxon was detected within a group, whereas relative abundance was computed as the proportion of each taxon relative to the total microbial community within individual samples.

## Supplementary Material

Extended PDF
