# Supplementary material for: Tryptophan Indole Metabolites Reduce Anastomotic Leakage Through Aryl Hydrocarbon Receptor-Driven Interleukin-22 Production
Source: Cell Mol Gastroenterol Hepatol. 2026 Feb 16;20(6):101756. doi: 10.1016/j.jcmgh.2026.101756 (PMC13094658; doi:10.1016/j.jcmgh.2026.101756)
Supplement: Supplementary file 1 [file mmc1.pdf]

Supplementary Table 1. Demographics of the clinical cohort.

CRC patients					
**Age**	**Sex**	**Tumor location**	**Stage**	**Type of surgery**	**AH/AL**
63	M	Left colon	T1	Left hemicolectomy	AH
67	M	Right colon	T2	Right hemicolectomy	AH
51	F	Rectum	T3	LAR	AH
66	M	Left colon	T3	LAR	AH
60	M	Left colon	T1	Left hemicolectomy	AH
76	M	Rectum	T0	LAR	AH
69	M	Rectum	T3	Robot LAR	AH
56	M	Left colon	T4	Sigmoid resection	AH
73	M	Left colon	T3	Robot LAR	AH
59	F	Left colon	T3	Sigmoid resection	AH
71	F	Transverse colon	Unclear	Extended right hemicolectomy	AH
79	M	Rectum	T2	Sigmoid resection	AH
81	M	Right colon	T3	Right hemicolectomy	AH
53	F	Rectum	T4	Robot PME	AH
75	M	Right colon	T3	Right hemicolectomy	AH
70	M	Left colon	T0	Sigmoid resection	AH
66	M	Right colon	T3	Right hemicolectomy	AH
75	M	Right colon	T0	Right hemicolectomy	AH
47	M	Left colon	T3	Robot PME	AH
71	M	Left colon	T1	Sigmoid resection	AH
57	M	Rectum	T2	TATME	AH
67	M	Left colon	T1	Sigmoid resection	AH
58	M	Left colon	T4	Sigmoid resection	AH
69	M	Right colon	T3	Right hemicolectomy	AH
75	M	Rectum	T2	TATME	AH
74	M	Left colon	T3	Sigmoid resection	AH
78	F	Left colon	T3	Left hemicolectomy	AH
76	M	Rectum	T2	LAR	AH
71	M	Left colon	T2	Sigmoid resection	AH
75	M	Rectum	T1	LAR	AH
75	M	Left colon	T3	LAR	AH
56	M	Left colon	T3	Sigmoid resection	AH
62	M	Left colon	T3	Sigmoid resection	AH
75	M	Rectum	T3	LAR	AL
71	M	Right colon	T4	Right hemicolectomy	AL
72	M	Rectum	T2	LAR	AL
52	M	Rectum	T3	LAR	AL
65	M	Rectum	T1	LAR	AL
59	M	Rectum	T3	Robot LAR	AL
75	M	Rectum	T3	Robot LAR	AL
71	M	Left colon	T2	Sigmoid resection	AL
51	F	Left colon	T3	Sigmoid resection	AL
83	M	Right colon	T3	Right hemicolectomy	AL
77	M	Left colon	T3	Left hemicolectomy	AL
61	F	Rectum	T3	LAR	AL
76	M	Rectum	T2	LAR	AL
68	M	Transverse colon	T2	Transversectomy	AL
73	M	Left colon	T1	Sigmoid resection	AL
75	F	Right colon	T1	Right hemicolectomy	AL
75	M	Rectum	T3	Sigmoid resection with extended left hemicolectomy	AL
58	F	Right colon	T3	Right hemicolectomy	AL
61	M	Left colon	T3	LAR	AL

Tryptophan metabolites	AH (n=33)	AL (n=19)	*P-value*
Tryptophan	6162	6311	0.5849
Indole-3-acetic acid	454.0	210.9	0.0678
Indole-3-aldehyde	172.4	244.1	0.5592
Indole-3-lactic acid	37.47	51.67	0.0947
Indole-3-acetamide	1.43[19]	1.43[14]	0.4977
Indole-3-sulfate	0.85[33]	0.85[19]	>0.999
Tryptophol	9.460	16.76	0.0708
Tryptamine	16.64	17.39	0.2262
Indole	218.0	156.2	0.9400
Kynurenine	5.310[4]	3.880[3]	0.6820
Kynurenic acid	102.9	73.59	0.6649
Xanthurenic acid	16.93[9]	19.61[6]	0.7029
Picolinic acid	1900	2037	0.5217
Quinolinic acid	1.660[17]	1.660[12]	0.5339
3-OH-Kynurenine	0.75[33]	0.75[19]	>0.999
3-OH-Anthranilic acid	3.880[13]	7.520[8]	0.6258
Serotonin	110.7	126.8	0.8360
5-OH-Tryptophan	2.310	3.120	0.7956
5-OH-Indole acetic acid	8.180[12]	10.32[7]	0.7720
Melatonin	0.000[33]	0.000[19]	>0.999
N-acetyl-serotonin	5.730	5.260	0.6960
Indoles (sum)	812.8	778.3	0.6513
IDO pathway (sum)	2067	2069	0.5341
5-HT pathway (sum)	133.6	151.7	0.8803
IDO activity (Kyn/Trp ratio)	0.00072	0.00044	0.3257
IAA/Kyn ratio	92.99	44.29	0.2158
IAA/Trp ratio	0.1053	0.04749	0.0771
IAA/Sum IDO ratio	0.2422	0.099	0.0100

Supplementary Table 2. Preoperative fecal concentrations of tryptophan metabolites in patients with colorectal cancer that underwent anastomotic surgery. Values resemble median amounts within each group. Number of samples below the detection limit are shown in square brackets. Statistical significance was determined by Mann-Whitney U test or unpaired t test for AL and AH comparisons. $**P < .01$.

Tryptophan metabolites	AH (n=28)	AL (n=15)	*P-value*
Tryptophan	5710	5937	0.7149
**Indole-3-acetic acid**	470.9	173.2	**0.0198**
Indole-3-aldehyde	173.1	276.8	0.4415
**Indole-3-lactic acid**	33.83 [1]	54.08	**0.0474**
Indole-3-acetamide	1.43[16]	1.43[4]	0.5491
Indole-3-sulfate	0.85[28]	0.85[15]	>0.999
Tryptophol	9.550	16.76	0.0954
Tryptamine	9.810	18.33	0.1373
Indole	215.2	155.0	0.6776
Kynurenine	4.815[4]	3.200[3]	0.7716
Kynurenic acid	82.97	69.02	0.3434
Xanthurenic acid	13.46[7]	17.85[5]	0.9542
Picolinic acid	1890	1910	0.6054
Quinolinic acid	1.950[13]	1.660[9]	0.5838
3-OH-Kynurenine	0.75[28]	0.75[15]	>0.999
3-OH-Anthranilic acid	4.135[10]	8.060[5]	0.3072
Serotonin	136.4	69.24	0.3699
5-OH-Tryptophan	2.240	3.160	0.5076
5-OH-Indole acetic acid	6.829[11]	9.740[3]	0.9068
Melatonin	0.000[28]	0.000[15]	>0.999
N-acetyl-serotonin	6.140	6.360	0.8653
Indoles (sum)	829.1	630.0	0.4567
IDO pathway (sum)	1999	2013	0.6231
5-HT pathway (sum)	151.2	84.16	0.3977
IDO activity (Kyn/Trp ratio)	0.00070	0.000062	0.6769
IAA/Kyn ratio	104.1	48.62	0.1373
**IAA/Trp ratio**	0.1066	0.0317	**0.0368**
**IAA/Sum IDO ratio**	0.2602	0.07783	**0.0030**

Supplementary Table 3. Preoperative fecal concentrations of tryptophan metabolites in male patients with colorectal cancer that underwent anastomotic surgery. Values resemble median amounts within each group. Number of samples below the detection limit are shown in square brackets. Statistical significance was determined by Mann-Whitney U test or unpaired t test for AL and AH comparisons. $**P < .01$.

Tryptophan metabolites	AH (n=5)	AL (n=4)	*P-value*
Tryptophan	6314	10004	0.9048
Indole-3-acetic acid	426.0	779.1	0.4127
Indole-3-aldehyde	167.5	175.4	0.9048
Indole-3-lactic acid	37.47	32.35	0.9048
Indole-3-acetamide	1.43[3]	1.43[3]	>0.999
Indole-3-sulfate	0.85[5]	0.85[5]	>0.999
Tryptophol	8.570	17.31	0.5556
Tryptamine	26.87	8.625	0.7302
Indole	218.0	293	0.7302
Kynurenine	5.310	3.965	0.9048
Kynurenic acid	121.5	164.9	0.2857
Xanthurenic acid	25.82[2]	51.97[1]	0.2778
Picolinic acid	2441	2584	0.7302
Quinolinic acid	1.660[4]	1.660[3]	>0.999
3-OH-Kynurenine	0.75[5]	0.75[4]	>0.999
3-OH-Anthranilic acid	1.550[3]	1.550[3]	0.5635
Serotonin	86.34	173.6	0.2857
5-OH-Tryptophan	2.710	2.035	0.5556
5-OH-Indole acetic acid	13.86[1]	18.0[1]	0.7937
Melatonin	0.000[5]	0.000[4]	>0.999
N-acetyl-serotonin	3.030	4.530	0.3175
Indoles (sum)	683.7	1337	0.5556
IDO pathway (sum)	2479	2808	0.7302
5-HT pathway (sum)	91.28	199.6	0.1111
IDO activity (Kyn/Trp ratio)	0.00089	0.00041	0.2857
IAA/Kyn ratio	190.1	263.8	0.5556
IAA/Trp ratio	0.02303	0.1152	0.9048
IAA/Sum IDO ratio	0.1366	0.2109	0.9048

Supplementary Table 4. Preoperative fecal concentrations of tryptophan metabolites in female patients with colorectal cancer that underwent anastomotic surgery. Values resemble median amounts within each group. Number of samples below the detection limit are shown in square brackets. Statistical significance was determined by Mann-Whitney U test for AL and AH comparisons.

	Anastomotic complication score (ACS)
	0 No adhesions or abnormalities
AH	1 Adhesion to fat pad, clean anastomosis underneath
	2 Adhesion to intestinal loop, abdominal wall or other organ
	3 Anastomotic defect found underneath adhesion, no other abnormalities
AL	4 Signs of possible contamination (e.g., small abscesses)
	5 Clear anastomotic complication; spread of pus, obstruction at anastomosis, sign of peritonitis
	6 Fecal peritonitis / Death due to peritonitis

Supplementary Table 5. ACS scoring system.

Tryptophan metabolites	Low (n=12)	High (n=8)	*P-value*
Tryptophan	71.16	4379	<0.0001
Indole-3-acetic acid	5.685	34.56	0.0002
Indole-3-aldehyde	6.375	25.80	<0.0001
Indole-3-lactic acid	5.640[1]	25.72	0.0124
Indole-3-acetamide	0.5300	0.8485	0.0001
Indole-3-sulfate	2.500[11]	2.500[5]	0.2206
Tryptophol	2.68	23.22	0.0001
Tryptamine	0.4500[12]	0.4500[8]	>0.999
Indole	26.195	97.025	0.0024
Kynurenine	11.38	60.01	0.0012
Kynurenic acid	7.685	18.42	0.0473
Xanthurenic acid	13.185	50.28	0.0095
Picolinic acid	747.0	725.0	0.6784
Quinolinic acid	46.335	675.49	<0.0001
3-OH-Kynurenine	0.135[12]	0.5455[1]	0.0006
3-OH-Anthranilic acid	1.045	4.170	0.0001
Serotonin	30.875	85.03	0.0001
5-OH-Tryptophan	3.615	13.19	0.0005
5-OH-Indole acetic acid	71.16	837.2	0.0387
Melatonin	0.015[7]	0.056[2]	0.0312
N-acetyl-serotonin	0.416	0.863	0.0238
Indoles (sum)	20.34	122.9	0.0001
IDO pathway (sum)	822.685	1685.61	0.0003
5-HT pathway (sum)	113.131	917.03	0.0062
IDO activity (Kyn/Trp)	0.01943	0.01323	0.0691

Supplementary Table 6. Fecal tryptophan metabolite concentrations after low- and high-tryptophan diets and before anastomotic surgery. Values resemble median amounts within each group. Number of samples below the detection limit are shown in square brackets. Statistical significance was determined by Mann-Whitney U test. $*P < .05$, $**P < .01$, $***P < .001$, $****P$ < A S.0001.

Supplementary Table 7. Composition of low- and high-tryptophan diets.

**Ingredients**	Product No.	**0.1 % Trp AA diet** S0016-E080	**1 % Trp AA diet** S0016-E084
Corn starch	%	30,000	30,000
Maltodextrin	%	16,000	16,000
Sucrose	%	18,077	17,170
Cellulose	%	5,000	5,000
L-Lysine HCl	%	1,800	1,800
DL-Methionine	%	0,700	0,700
L-Cystine	%	0,350	0,350
L-Threonine	%	0,900	0,900
L-Tryptophan	%	0,103	1,010
L-Arginine	%	1,000	1,000
L-Histidine	%	0,500	0,500
L-Valine	%	0,900	0,900
L-Isoleucine	%	0,820	0,820
L-Leucine	%	1,200	1,200
L-Phenylalanine	%	0,750	0,750
L-Tyrosine	%	0,500	0,500
Glycine	%	2,500	2,500
L-Glutamic acid	%	3,000	3,000
L-Glutamine	%	1,000	1,000
L-Aspartic acid	%	0,350	0,350
L-Asparagine	%	0,600	0,600
L-Proline	%	0,400	0,400
L-Alanine	%	0,400	0,400
L-Serine	%	0,350	0,350
Vitamin premix	%	1,000	1,000
Minerals	%	4,500	4,500
Choline Cl	%	0,250	0,250
Butylated hydroxytoluene	%	0,010	0,010
Food dye	%	0,040	0,040
Soybean oil	%	7,000	7,000
Proximate contents			
Crude protein (N x 6.25)	%	16,4	17,2
Crude fat	%	7,0	7,0
Crude fiber	%	5,0	5,0
Crude ash	%	3,4	3,4
Starch	%	28,9	28,9
Sugar	%	19,3	18,4
Dextrins	%	15,8	15,8
Lysine	%	1,40	1,40
Methionine	%	0,69	0,69
**Ingredients**	Product No.	**0.1 % Trp AA diet** S0016-E080	**1 % Trp AA diet** S0016-E084
Cystine	%	0,35	0,35
Met+ Cys	%	1,03	1,03
Threonine	%	0,88	0,88
Tryptophan	%	0,10	1,00
Energy (Atwater)	MJ/kg	16,7	16,7
Protein	kJ%	16	17
Fat	kJ%	16	16
Carbohydrates	kJ%	68	67

These diets are a modification of V1534-300

Supplementary Table 8. Differential abundance of ASVs (DESeq2 *P*-adj < .05).

	log2FoldChange	padj
ASVX_226_Clostridiales_vadinBB60_group	-6.830659375	6.88E-06
ASVX_224_Lachnospiraceae	-6.517952821	9.35E-06
ASVX_339_Lachnospiraceae	-5.330281352	0.0007836
ASVX_236_Clostridiales_vadinBB60_group	-5.265350626	0.00046453
ASVX_209_Lachnospiraceae	-4.610986513	0.00013535
SVX_273_Lachnospiraceae_NK4A136_group_N	-4.588031293	0.00214944
ASVX_278_Alistipes_NA	-4.313172975	0.00536893
ASVX_684_Desulfovibrionaceae	-4.241600909	0.00536893
ASVX_227_Lachnospiraceae	-4.229238314	0.00757519
ASVX_698_Faecalibaculum_NA	-4.216525926	0.00536893
ASVX_122_Lachnospiraceae	-4.075905095	0.00881748
ASVX_270_Muribaculaceae	-3.9570562	0.01087137
ASVX_121_Oscillibacter_NA	-3.890236064	0.00536893
ASVX_225_Lachnospiraceae	-3.741763898	0.01135497
ASVX_554_Lachnospiraceae	-3.340463185	0.0193342
ASVX_676_Lachnoclostridium_NA	-3.309262705	0.03640394
ASVX_181_Ruminiclostridium_9_NA	-3.270845237	0.02853696
SVX_347_Lachnospiraceae_NK4A136_group_N	-3.132495481	0.04735964
ASVX_157_Muribaculaceae	-3.011278664	0.03877034
ASVX_654_Lachnospiraceae	-3.005747703	0.03877034
ASVX_64_Anaerotruncus_colihominis	-2.999836583	0.01380567
ASVX_158_Lachnospiraceae	-2.990286734	0.02447756
ASVX_394_Muribaculaceae	-2.976956639	0.04685539
ASVX_545_Lachnospiraceae	-2.906211901	0.02853696
ASVX_993_Akkermansia_NA	-2.863992899	0.04978538
ASVX_92_Lachnospiraceae_UCG-006_NA	-2.738845325	0.03557737
ASVX_212_Muribaculaceae	2.151241055	0.0459244
ASVX_440_Ruminococcaceae_UCG-014_NA	2.32488226	0.04978538
ASVX_845_Ruminiclostridium_9_NA	2.324916017	0.03877034
ASVX_945_Lachnoclostridium_NA	2.492999423	0.04182268
ASVX_162_Turicibacter_NA	2.536135834	0.04982823
SVX_737_Lachnospiraceae_NK4A136_group_N	2.611353433	0.035856
ASVX_691_Lachnospiraceae	2.693928727	0.03135669
ASVX_582_GCA-900066575_NA	2.709177966	0.02884301
ASVX_564_Parasutterella_NA	2.815020094	0.04224675
SVX_504_Lachnospiraceae_NK4A136_group_N	2.925287207	0.02924428
ASVX_346_Clostridiales_vadinBB60_group	2.976875285	0.00984503
ASVX_535_Ruminiclostridium_NA	3.014435434	0.01270784
ASVX_565_Ruminiclostridium_9_NA	3.05703631	0.00990535
ASVX_280_Lachnospiraceae	3.072218315	0.00990535
ASVX_567_Bacteroides_NA	3.09445938	0.01489324
ASVX_551_Flavonifractor_NA	3.100700942	0.01636591
ASVX_522_Intestinimonas_NA	3.206364591	0.00984503
SVX_232_Lachnospiraceae_NK4A136_group_N	3.285813271	0.01730885
SVX_492_Lachnospiraceae_NK4A136_group_N	3.41066325	0.00933085
ASVX_254_Parasutterella_NA	3.442143263	0.0007836
ASVX_599_Ruminococcaceae_UCG-014_NA	3.49380836	0.00790046

	log2FoldChange	padj
ASVX_207_Anaerotruncus_NA	3.574508393	0.00547841
ASVX_399_Clostridiales_vadinBB60_group	4.369898664	0.00081166
SVX_471_Lachnospiraceae_NK4A136_group_N	4.546416478	0.00081166
ASVX_319_Pseudomonas_NA	5.139069309	0.0005343
ASVX_450_Ruminococcaceae_UCG-014_NA	6.139239776	4.04E-06

Supplementary Table 9. qPCR primers used for gene expression analysis.

Gene	Forward Sequence (5'-3')	Reverse Sequence (5'-3')
AhR	TCTAAGCGACACAGAGACCG	GGTGGACTTTAATGCAACATCA
Cyp1a1	CCTCATGTACCTGGTAACCA	AAGGATGAATGCCGGAAGGT
IL-22	CATGCAGGAGGTGGTACCTT	CAGACGCAAGCATTTCTCAG
IL-17A	TTTAACTCCCTTGGCGCAAAA	CTTTCCCTCCGCATTGACAC
IL-22RA1	GGAGATAAGTTGCTTCCCCAAA	CATCCACGCCCAGCCATAG
IL-23	AGCGGGACATATGAATCTACTAAGAGA	GTCCTAGTAGGGAGGTGTGAAGTTG
IL-10	AGAAGCATGGCCCAGAAATCA	GGCCTTGTAGACACCTTGGT
Reg3γ	TTCCTGTCCTCCATGATCAAAA	CATCCACCTCTGTTGGGTTCA
Reg3β	ATGCCGAAGACGATCAGTGTG	ACCAAACTTCCCTCAAACCAAT
IL-6	AAGTCGGAGGCTTAATTACACATGT	CCATTGCACAACTCTTTTCTCATT
IL-1β	TTGACGGACCCCAAAAGATG	CAGGACAGCCCAGGTCAAAG
S100a8	ATGCCGTCTGAACTGGAGAA	CGATATTTATATTCTGCACAAACTGAG
SOCS3	CCACCTGGACTCCTATGAGAAAGT	CCTCTGACCCTTTTGCTCCTTA
GAPDH	TaqMan™ probe<br>NM_008084.2 (Applied Biosystems)	
