# Supplementary material for: Tryptophan Indole Metabolites Reduce Anastomotic Leakage Through Aryl Hydrocarbon Receptor-Driven Interleukin-22 Production
Source: Cell Mol Gastroenterol Hepatol. 2026 Feb 16;20(6):101756. doi: 10.1016/j.jcmgh.2026.101756 (PMC13094658; doi:10.1016/j.jcmgh.2026.101756)
Supplement: Extended PDF [file mmc2.pdf]

cmgh
CELLULAR AND MOLECULAR GASTROENTEROLOGY AND HEPATOLOGY

ORIGINAL RESEARCH

Tryptophan Indole Metabolites Reduce Anastomotic Leakage Through Aryl Hydrocarbon Receptor-Driven Interleukin-22 Production

Vasiliki Bantavi,[1,2] Laura Gloeck,[1] Patrick Leven,[1] Konstantina Zafeiropoulou,[2,3] Olaf Welting,[2] Hilal Sengül,[1,2] Patrik Efferz,[1] Bianca Schneiker,[1] Timothy Ryan McCulloch,[4] Christoph Wilhelm,[4] Sandra Blaess,[6] Anneke Fuss,[6] REVEAL study group, Joep P. M. Derikx,[3] Bruno Sovran,[2,3,5] Wouter J. de Jonge,[1,2,§] and Sven Wehner[1,§]

[1]Department of General, Visceral, Thoracic, and Vascular Surgery, University Hospital Bonn, University of Bonn, Bonn, Germany; [2]Tytgat Institute for Liver and Intestinal Research, Amsterdam Gastroenterology Endocrinology and Metabolism, Amsterdam University Medical Centers, University of Amsterdam, Amsterdam, The Netherlands; [3]Department of Pediatric Surgery, Emma Children's Hospital, Amsterdam University Medical Centers, University of Amsterdam, Amsterdam, The Netherlands; [4]Immunopathology Unit, Institute of Clinical Chemistry and Clinical Pharmacology, University Hospital Bonn, University of Bonn, Bonn, Germany; [5]Emma Center for Personalized Medicine, Amsterdam University Medical Center (UMC), University of Amsterdam, Amsterdam, The Netherlands; and [6]Neurodevelopmental Genetics, Medical Faculty, Institute of Reconstructive Neurobiology, University of Bonn, Bonn, Germany

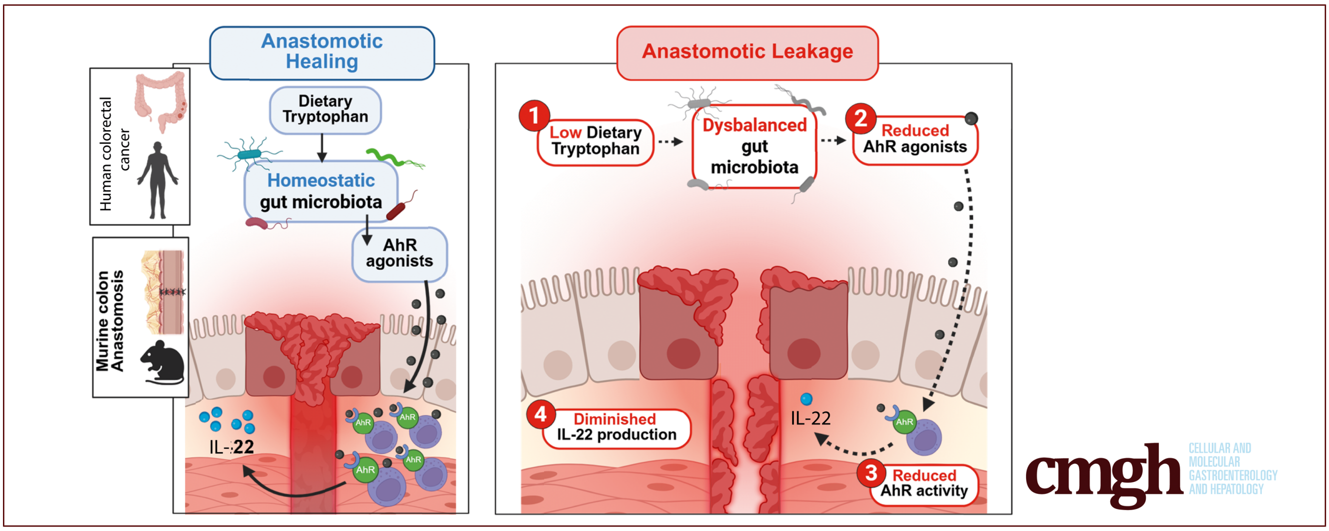

SUMMARY

Male patients with anastomotic leakage showed reduced preoperative fecal levels of indole-3 acetic acid. Aryl hydrocarbon receptor promotes anastomotic healing via the interleuken-22 signaling pathway. Aryl hydrocarbon receptor agonists rescue low tryptophan diet-induced healing disturbances in mice.

WHAT YOU NEED TO KNOW

Background: Preoperative microbiome composition and tryptophan-derived indole metabolites that activate aryl hydrocarbon receptors (AhRs) influence intestinal healing; we examined their role in anastomotic leakage in a colorectal cancer patient cohort and a colon-anastomosis mouse model.

Impact: AhR activation through interleukin-22 enhances anastomotic healing, suggesting that stimulating this pathway could reduce the risk of anastomotic leakage. Both synthetic agonists and dietary tryptophan metabolites show therapeutic potential.

Future Directions: Future studies should focus on optimizing AhR/interleukin-22-based therapies for anastomotic leakage prevention, exploring the role of diet and AhR

agonists in clinical settings, and evaluating postoperative outcomes in patients.

BACKGROUND & AIMS: Colorectal cancer (CRC) often requires surgical resection of the tumor and an anastomosis. Anastomotic leakage (AL) occurs in 2.8% to 30% of patients, which increases postoperative morbidity and complications. The preoperative microbiome composition is implicated in AL. Recent studies have shown that microbial tryptophan (Trp) metabolism into aryl hydrocarbon receptor (AhR) indole-derivatives contributes to intestinal tissue healing. Here, we addressed the role of Trp and its metabolites in AL in a CRC patient cohort and a colon-anastomosis mouse model.

METHODS: Targeted quantitative metabolomics was performed in preoperative fecal samples from patients with CRC recruited in the REVEAL cohort (n = 388), including 19 AL cases. Anastomotic healing (AH) was tested in a mouse model using wild-type (WT), *AhR*$^{-/-}$, *Villin*^{Cre}*Ahr*$^{fl/fl}$, and interleukin (IL)-22 drug-targeting to evaluate the role of AhR and IL-22 in AL.

RESULTS: Fifty-two of the 388 patients with available preoperative fecal samples were matched for AH/AL occurrence (AL, n = 19; AH, n = 33). Among Trp metabolites, indole-3-acetic acid was significantly reduced in AL compared with matched AH male patients. *AhR*$^{-/-}$ mice displayed more severe AL, reduced IL-22 expression, and a marked loss of IL-22-expressing type 3 ILCs compared with WT mice. Neutralizing IL-22 antibody augmented AL in WT mice, whereas IL-22Fc application ameliorated AL in *AhR*$^{-/-}$ mice. AhR agonism failed to rescue healing under IL-22 deficiency. Furthermore, low-Trp diet-fed WT mice exhibited reduced fecal concentration of AhR agonists, AhR-agonist producing bacteria, and augmented AL. This phenotype was prevented by dietary supplementation with the AhR agonist indole-3-carbinol.

CONCLUSIONS: Stimulation of the AhR/IL-22 by synthetic agonists or dietary-derived Trp-metabolites can prevent AL. *(Cell Mol Gastroenterol Hepatol 2026;20:101756; https://doi.org/10.1016/j.jcmgh.2026.101756)*

Keywords: Aryl Hydrocarbon Receptor; Interleukin-22; Intestinal Anastomotic Leakage; Tryptophan Metabolism.

Anastomotic leakage (AL) is a detrimental complication after colorectal surgery, wherein the anastomosis heals incompletely, leading to infections and disturbed gut barrier function.[1–3] It can occur in up to 30% of patients after intestinal resection of the colon or rectum and is more prevalent in distal colorectal and coloanal anastomosis.[4] AL can lead to complications ranging from small abscess formation to peritonitis, sepsis, and even death if left untreated or treatment is delayed.[5] It is associated with higher mortality and significant morbidity rates, which result in prolonged hospitalization and increased health care costs.[6] The etiopathogenesis of AL is not entirely known, and it is recognized as a multifactorial disease where host genetics, gut microbiota, and perioperative inflammation all contribute to risk of leakage.[1]

Disturbance in the gut microbiota composition assumed to play a role in AL as in many chronic inflammatory conditions, such as celiac disease, inflammatory bowel diseases (IBDs), and post-infectious irritable bowel syndrome.[7–11] Evidence of the impact of gut microbiota on AL was first demonstrated by Shogan et al, showing that postoperative accumulation of *Enterococcus faecalis*, a collagenolytic bacterium, impairs anastomotic healing (AH) and triggers AL.[12] More recently, Hajjar et al associated specific preoperative microbiota with the pathogenesis of AL in patients undergoing colorectal cancer (CRC) surgery.[13]

Microbial metabolites of tryptophan (Trp), including kynurenine, serotonin, and indole or its derivatives, act as agonists of the aryl hydrocarbon receptor (AhR).[14,15] It is known that AhR activation regulates the production of certain cytokines, such as interleukin (IL)-10, IL-17, and IL-22, in immune cells, which all play pivotal roles in mucosal healing.[11,16,17] Interestingly, inflammatory lesions were associated with altered Trp metabolism, leading to reduced levels of AhR agonists and its downstream target, IL-22.[14,18,19]

In the current study, we investigated the protective role of indoles against AL in a patient cohort and a mouse model. We particularly described a critical role of indole formation in activating the AhR-driven IL-22 production to support AH.

Results

Reduced Levels of Fecal Indole-3-acetic Acid Associate With Anastomotic Leakage in Patients With CRC

To address the role of gut luminal AhR agonists in patients with AL, we analyzed the concentrations of Trp

[§]Authors share co-senior authorship.

Abbreviations used in this paper: 5-HT, 5-hydroxytryptamine; ACS, anastomotic complication score; AH, anastomotic healing; AhR, aryl hydrocarbon receptor; AL, anastomotic leakage; AMP, antimicrobial peptide; ANOVA, analysis of variance; aRW, anti-ragweed isotype control; ASV, amplicon sequence variant; BMI, body mass index; cDNA, complementary DNA; CRC, colorectal cancer; CTRL, control; DAPI, 4′,6-diamidino-2-phenylindole; DIM, 3,3′-diindolylmethane; DMEM, Dulbecco's Modified Eagle's Medium; DMSO, dimethyl sulfoxide; DSS, dextran sulfate sodium; DTT, dithiothreitol; FBS, fetal bovine serum; FC, fold change; FICZ, formylindolo[3,2-b] carbazole; HRP, horseradish peroxidase; IAA, indole-3-acetic acid; IBD, inflammatory bowel disease; ICZ, indolo [3,2-b] carbazole; IDO, indoleamine 2,3-dioxygenase; IEC, intestinal epithelial cell; IL, interleukin; ILA, indole-3-lactic acid; ILC, innate leukocyte cells; I3C, indole-3-carbinol; IMDM, Iscove's Modified Dulbecco's Medium; LC-HRMS, liquid chromatography coupled with high-resolution mass spectrometry; LC-MS, liquid chromatography-mass spectrometry; mRNA, messenger RNA; nAB, neutralizing antibody; NOD, nonobese diabetic; PBS, phosphate-buffered saline; PCoA, principal coordinate analysis; PERMANOVA, permutational multivariate analysis of variance; PFA, paraformaldehyde; POD, postoperative day; RT-qPCR, quantitative reverse transcription-polymerase chain reaction; rRNA, ribosomal RNA sequencing; SD, standard deviation; SEM, standard error of the mean; SPF, specific pathogen-free; STAR, Stool Transport and Recovery; Trp, tryptophan; TSA, tyramide signal amplification; WT, wild-type.

Most current article

© 2026 The Authors. Published by Elsevier Inc. on behalf of American Gastroenterological Association Institute. This is an open access article under the CC BY license (http://creativecommons.org/licenses/by/4.0/).
2352-345X
https://doi.org/10.1016/j.jcmgh.2026.101756

A

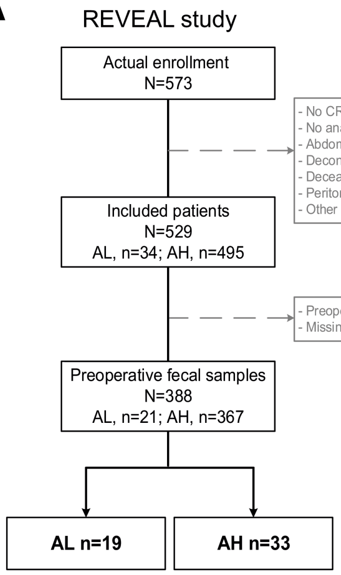

B

		AH	AL	*P-value*
Number of subjects	NA	33	19	NA
Age (years)	non-normally distributed => Mann-Whitney U	69 (8.9)	71 (9.0)	0.654
BMI (Kg/m^2)		25.7 (3.1)	25.6 (3.5)	0.739
Sex (M/F)	Pearson X2	28/5	15/4	0.588
Bowel preparation		15 (45.5)	7 (36.8)	0.545
CRC stage		[1]		
0		0 (0.0)	0 (0.0)	
I		9 (28.1)	5 (26.3)	
II		6 (18.8)	6 (31.6)	
III		15 (46.9)	7 (36.8)	
IV		2 (6.2)	1 (5.3)	0.783
Type of operation		[7]	[3]	
Left hemicolectomy		3 (11.5)	1 (6.2)	
Transverse colectomy		0 (0.0)	1 (6.2)	
Right hemicolectomy		6 (23.1)	4 (25.0)	
Sigmoid resection		11 (42.3)	3 (18.8)	
Rectosigmoid resection		6 (23.1)	7 (43.8)	0.293
Surgical approach				
Open		0 (0.0)	1 (5.3)	
Laparoscopic		31 (93.9)	18 (94.7)	
Conversion		2 (6.1)	0 (0.0)	0.236
Previous abdominal surge		11 (33.3)	7 (38.9) [1]	0.382
Comorbidities				
Diabetes Mellitus		4 (12.1)	5 (26.3)	0.193
IBD		0 (0.0)	0 (0.0)	NA
Neoadjuvant therapy		[3]		
None		22 (73.3)	14 (73.6)	
Radiotherapy		2 (6.7)	1 (5.3)	
Chemotherapy		2 (6.7)	1 (5.3)	
Both		4 (13.3)	3 (15.8)	0.989
Smoking		3 (9.1)	3 (15.8)	0,467*

C

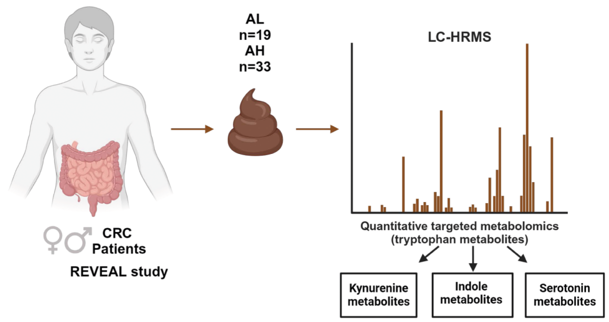

D **E** **F** **G**

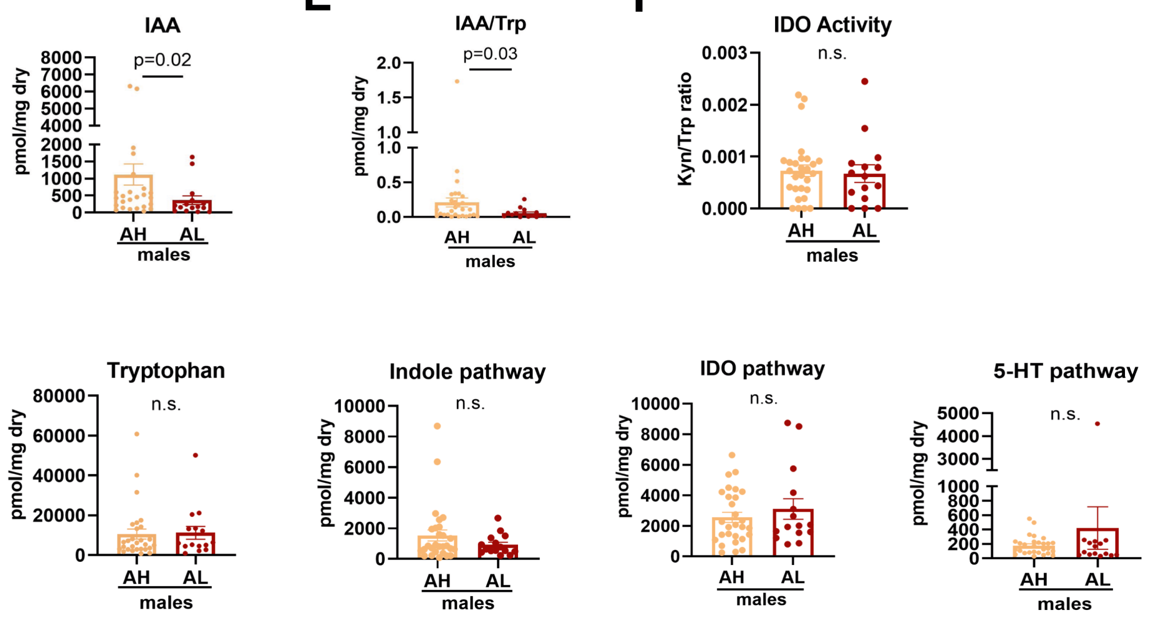

metabolites in preoperative stool samples from patients of the REVEAL study (Figure 1*A*).[20] Preoperative stool samples could be collected from 388 of 529 included patients, of whom 21 developed AL, whereas 367 presented with normal AH (Figure 1*B*). Trp metabolomics was performed on 19 patients with AL (15 males, 4 females) and 33 patients with AH (28 males, 5 females) with similar clinical characteristics (Figure 1*C* and Supplementary Table 1). Analysis showed a trend towards higher levels of indole-3-acetic acid (IAA), a known AhR agonist[14,16] in patients with AH vs AL ($P = .06$) (Supplementary Table 2), but no difference in the other metabolites related to the indole, kynurenine (indoleamine 2,3-dioxygenase [IDO]), or serotonin (5-hydroxytryptamine [5-HT]) pathway or in Trp itself (Supplementary Table 2) between patients with AL and AH. The overall IDO activity, determined by the kynurenine-to-Trp ratio, also remained unchanged between the 2 groups (Supplementary Table 2). Given that male sex is a risk factor for AL, we then compared Trp metabolite levels within the male patients and observed that the IAA and indole-3-lactic acid (ILA) concentrations were significantly higher in the stool of healing male patients compared with patients with AL ($P = .02$ and $P = .04$, respectively) (Figure 1*D*, Supplementary Table 3). Supportingly, the IAA-to-Trp ratio was higher in male patients with AH compared with male patients with AL ($P = .03$), suggesting that Trp is metabolized towards the indole pathway (Figure 1*E*). No difference in the IDO activity (Figure 1*F*), Trp itself, or the other metabolites related to the indole, kynurenine (IDO), or serotonin (5-HT) pathway was observed (Figure 1*G*, Supplementary Table 3). Analysis of female patients showed no difference in the metabolites between AH and AL (Supplementary Table 4).

AhR Deficiency Leads to Enhanced Rates of AL in a Mouse Model of Colon Anastomosis

Given that IAA is a known AhR agonist,[16] we evaluated the role of AhR in AH/AL in AhR-deficient ($AhR^{-/-}$) and wild-type (WT) mice at different time points after an end-to-end proximal colon anastomosis (Figure 2*A*). Anastomosis was assessed on postoperative day (POD) 1 and 5 using an anastomotic complication score (ACS),[21] with ACS $\geq$3 representing AL (Figure 2*B*, Supplementary Table 5). On POD1, AL rates increased in $AhR^{-/-}$ (90.9% ACS $\geq$3) compared with WT mice (18.2% ACS $\geq$3; $P < .001$) (Figure 2*C*–*E*). On POD5, ACS scores were still elevated in $AhR^{-/-}$ mice compared with WT mice ($P < .05$), with 2 of 11 AL mice presenting the highest AL levels (ACS = 5–6), characterized by severe abscess formation, spread of pus, and signs of fecal peritonitis, (Figure 2*C*–*E*).

Next, we analyzed the expression of AhR and its downstream target genes, including immune-related and host-defense genes. Notably, these analyses were initially performed on POD1, as the immune response is most prominent within the first day after surgery (Figure 2*F*). *AhR* expression was detected in WT but not in $AhR^{-/-}$ mice (Figure 2*F*). Interestingly, we found a notable increase (144-fold; $P <$ .0001) in expression of *IL-22*, a cytokine acting on epithelial cells[22,23] and supporting barrier function in the anastomotic tissue of WT mice. Notably, *IL-22* expression remained significantly low in the anastomotic tissue of $AhR^{-/-}$ mice (Figure 2*F*). We also investigated IL-22 expression as well as the expression of some of the canonical *IL-22* regulated genes, namely *Reg3b*, *Reg3g*, and *IL-22RA1*, one of the *IL-22* receptors expressed by epithelial cells, at both 6 hours and on POD1 tissues (Figure 2*F* and *G*). At 6 hours, the expression of *IL-22* was higher than at POD1 in WT mice (151-fold; $P = .06$) (Figure 2*G*). The expression of *Reg3b* and *Reg3g* was significantly altered between naive colon (control [CTRL]) of WT and $AhR^{-/-}$ mice at 6 hours and only for *Reg3b* on POD1, although we observed no difference in *IL-22RA1* expression between WT and $AhR^{-/-}$ at either time point (Figure 2*F* and *G*). Additionally, we investigated the expression of other pro- and anti-inflammatory cytokines, such as *IL-17A*, *IL-23*, *IL-10*, and *IL-6*, previously linked to the AhR pathway (Figure 2*F*). Levels of *IL-6* were significantly higher in the anastomotic tissue in both WT and $AhR^{-/-}$ mice compared with their respective naïve tissues, but unlike *IL-22*, there was no significant difference between WT and $AhR^{-/-}$ mice (Figure 2*F*). *IL-17A* showed a trend of downregulation in the anastomotic tissue of $AhR^{-/-}$ compared to WT mice (Figure 2*F*), whereas *IL-10* and *IL-23* showed no difference between genotypes (Figure 2*F*) and a slight increase in $AhR^{-/-}$ anastomosis compared to naïve controls. Together, AhR-driven *IL-22* gene expression is strongly induced during anastomosis.

AhR-Deficient Mice Display Diminished Numbers of IL-22-Producing ILC3s

As *IL-22*, as well as its downstream targets *Reg3b* and *Reg3g*, were significantly reduced in anastomotic tissue of $AhR^{-/-}$ mice compared with WT mice, we wanted to investigate its cellular origin. Using RNAscope, we were able to visualize in situ IL-22 mRNA expression in naïve WT tissue and 6 hours after anastomosis surgery, the time point when anastomotic *IL-22* mRNA expression was highest (Figure 3*A*). Interestingly, *IL-22* mRNA expression appeared tightly clustered in the anastomotic region of WT mice, whereas it was absent in both naïve WT and $AhR^{-/-}$ mice. Additionally, $AhR^{-/-}$ mice displayed a strongly reduced *IL-22* mRNA signal in

Figure 1. (***See previous page***). **REVEAL study.** (*A*) Scheme of the REVEAL study for Trp metabolite analysis. (*B*) Flow chart of the REVEAL study. (*C*) Patient characteristics. (*D*) Concentration of IAA in the preoperative stool samples of male patients (AH: n = 28; AL: n = 15) that underwent CRC surgery. (*E*) IAA/Trp ratio in males. (*F*) IDO activity (calculated as the kynurenine-to-Trp ratio) in male patients. (*G*) Quantification of Trp and total metabolites from the indole, kynurenine (IDO) and serotonin (5-HT) pathway in stool of male patients. Each dot represents a patient, with bars showing mean ± SEM. Statistical significance was determined by Mann-Whitney *U* test or Pearson χ^2. Numerical variables resemble the median. Categorical variables: n (% within group), percentages have been calculated in the available data. Square brackets indicate missing data. *Previous smokers are included in the ''No'' group. *$P < .05$.

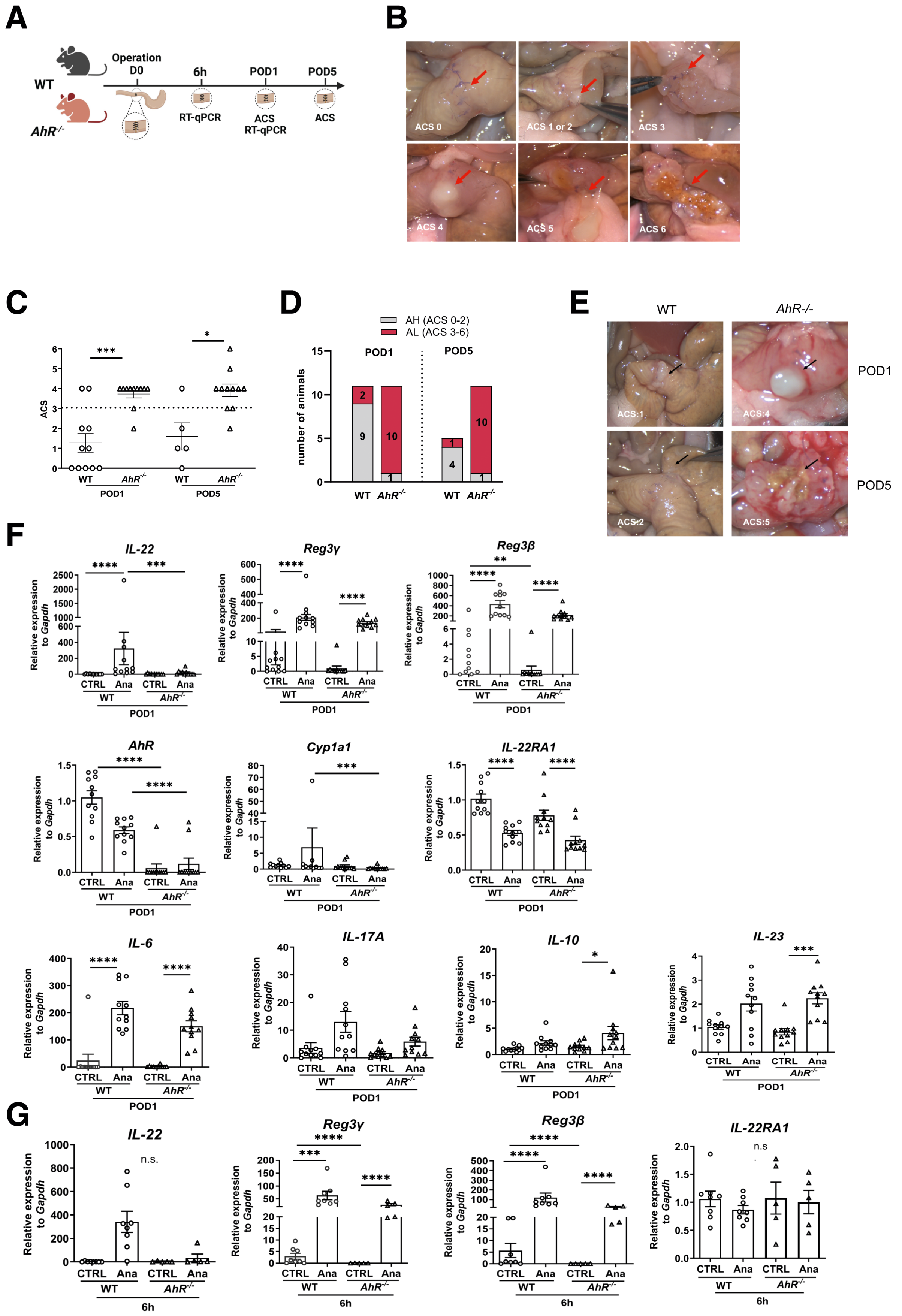

anastomotic tissue (Figure 3*A*). Next, we investigated the cellular origin of IL-22 by flow cytometry. As the AhR is important for the development of type 3 innate leukocyte cells (ILCs),[24] we first characterized both ILC2 and ILC3 populations in both WT and $AhR^{-/-}$ mice. Although we observed no difference in overall total ILC numbers, total ILC2s were increased, whereas ILC3s were reduced in $AhR^{-/-}$ mice ($P = .0848$) (Figure 3*B* and *C*). IL-22 is known to be produced by multiple immune cells, including various types of lymphoid cells, but not epithelial cells.[25] Analysis of cells isolated from the anastomosis for their capacity to produce IL-22 revealed ILCs, $\gamma\delta$ T cells, and $CD4^+$ T cells as cellular origin in both WT and $AhR^{-/-}$ mice. However, only IL-22^+ ILCs were significantly reduced in $AhR^{-/-}$ compared with WT mice (Figure 3*D* and *E*). These findings highlight the importance of AhR signaling and ILCs in AL, indicating that a reduction of ILC3s subsequently induces IL-22 deficiency, which might be one of the molecular pathways behind AL.

AhR-Driven IL-22 Supports Anastomotic Healing

Consequently, we studied the role of IL-22 in healing and AL. Treatment with recombinant IL-22 induced faster wound closure in an epithelial scratch assay (Figure 4*A* and *B*). To study the potential supportive role of IL-22 in vivo, we first treated WT mice with an anti-IL-22 neutralizing antibody (aIL-22 nAb) (Figure 4*C*). The aIL-22 treatment strongly increased AL rates (Figure 4*D*–*F*), underlining the essential role of IL-22 in transmural wound healing. In addition, aIL-22 nAb treatment revealed a significant reduction of *Reg3b* and *Reg3g*, 2 downstream target genes of IL-22 (Figure 4*G*). We also investigated the expression of *IL-6, IL-1β, S100a8,* and *Socs3*, but did not observe any difference between aIL-22- and IgG1a-treated mice, suggesting a distinct role for IL-22 in its potential to prevent AL (Figure 4*H*).

Given the supportive role of AhR and IL-22 in AH, we investigated whether intraperitoneal application of recombinant IL-22 protein rescues $AhR^{-/-}$ mice from AL (Figure 4*I*). $AhR^{-/-}$ mice treated with recombinant IL-22Fc protein displayed reduced AL compared with the anti-ragweed isotype control (aRW)-treated littermates, albeit not significant ($P = .09$) (Figure 4*J*). These data indicate that IL-22 can partially compensate for the absence of AhR, supporting its role as a downstream mediator of AhR-dependent intestinal healing.

AhR Agonism via Indole-3-Carbinol Depends on IL-22 Signaling for Anastomotic Healing

To determine whether AhR-driven AH requires IL-22 activation, we next examined the effect of blocking IL-22 while activating AhR through the AhR agonist indole-3-carbinol (I3C). WT mice were given either I3C or vehicle by oral gavage, and then they were injected with the aIL-22 antibody (Figure 4*K*; n = 16 per group). One mouse from the I3C-treated group was excluded from the analysis as it was euthanized on POD4 to meet humane endpoint criteria. Both groups exhibited impaired AH, as evidenced by comparable high ACS, with no significant difference between I3C- and vehicle-treated mice ($P = .5899$) (Figure 4*I*). These findings indicate that neutralization of IL-22 profoundly compromises anastomotic healing, and that AhR agonism through I3C exerts its effects on AH predominantly via the IL-22 signaling pathway.

Epithelial AhR Activation Is Involved in Anastomotic Healing

Because epithelial expression of AhR is known to support intestinal epithelial healing,[26] we also aimed to elucidate if epithelial AhR activation contributes to AH. First, to prove that AhR is essential for epithelial regeneration, we performed an in vitro experiment. In a scratch assay, carried out with HT29 human intestinal epithelial cells (IECs), treatment with the AhR agonists FICZ (6-formylindolo[3,2b] carbazole), or IAA accelerated wound closure, but not I3C (Figure 5*A* and *B*). The process could be blocked by simultaneous addition of the AhR inhibitor CH223191 in all treatments, demonstrating a role for AhR in epithelial wound healing (Figure 5*A* and *B*).

Next, we assessed whether epithelial AhR-signaling also affects in vivo full thickness bowel wall healing in $Villin^{Cre}$ $AhR^{fl/fl}$ mice and their Cre-negative littermates subjected to the colon anastomosis model (Figure 5*C*). A comparison of AL severity levels between $Villin^{Cre}AhR^{fl/fl}$ and full $AhR^{-/-}$ mice showed that $AhR^{-/-}$ mice have higher ACS scores than $Villin^{Cre}AhR^{fl/fl}$ mice (Figure 5*F*). However, this effect was not present when AH was compared between $Villin^{Cre}AhR^{fl/fl}$ mice and their Cre-littermates, although the latter had slightly but not significantly reduced numbers of animals with ACS score ≥3 compared with their $Villin^{Cre}AhR^{fl/fl}$ littermate mice ($P = .14$). (Figure 5*D* and *E*). These findings reveal that, although AhR-signaling supports epithelial healing in vitro, it does not play a dominant role in AH in vivo.

Figure 2. (***See previous page***). **AhR deficient mice are sensitive to colonic AL.** (*A*) Scheme of the surgical method and timeline. (*B*) Representative images for each score. (*C*) ACS in WT and $AhR^{-/-}$ mice showed a difference between the 2 groups on POD1 (WT: n = 11; $AhR^{-/-}$: n = 11) and on POD5 (WT: n = 5; $AhR^{-/-}$: n = 11). (*D*) $AhR^{-/-}$ mice showed more frequent AL (ACS ≥3) compared with WT mice on POD1, and on POD5. (*E*) Representative in situ images of WT and $AhR^{-/-}$ mice on POD1 and POD5. (*F* and *G*) Gene expression of AhR-related genes in anastomotic (Ana) and naïve colonic tissues (CTRL) collected on POD1 (*E*; $AhR^{-/-}$: n = 11; WT: n = 11) and 6 hours postoperatively (*F*; $AhR^{-/-}$: n = 5; WT: n = 8). Each dot represents 1 mouse, with bars showing mean ± SEM. *Arrows* indicate the anastomosis and the complications. Statistical significance was determined by Mann-Whitney *U* test (*C*; comparison of groups per timepoint) or 2-way ANOVA with Tukey's post hoc test (*G* and *H*). *$P < .05$; **$P < .01$; ***$P < .001$; ****$P < .0001$.

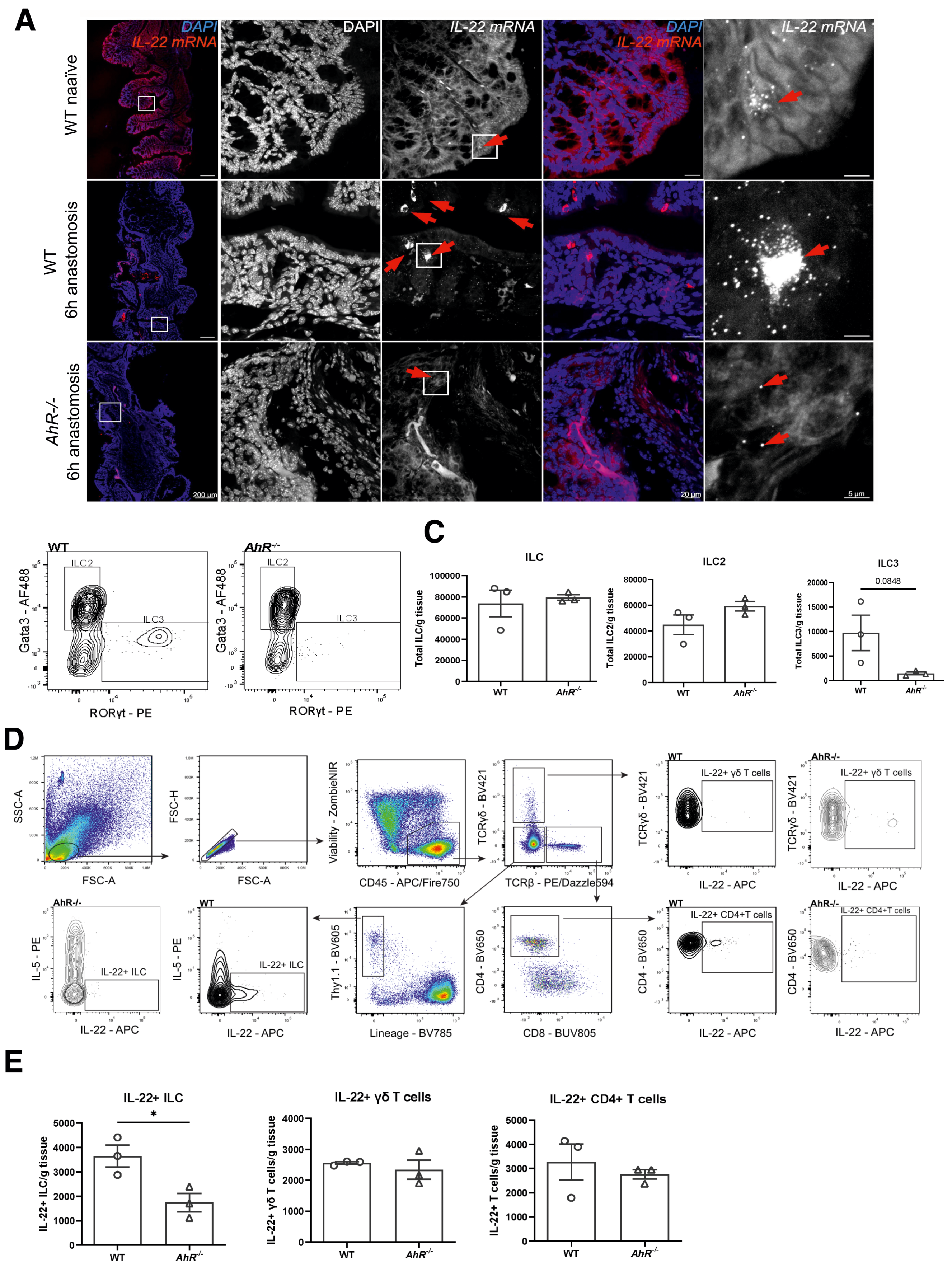
A
DAPI
IL-22 mRNA
WT naaïve
WT 6h anastomosis
AhR-/- 6h anastomosis
200 μm
20 μm
5 μm
WT
AhR-/-
ILC2
ILC3
Gata3 - AF488
RORγt - PE
C
ILC
ILC2
ILC3
0.0848
Total ILC/g tissue
Total ILC2/g tissue
Total ILC3/g tissue
D
SSC-A
FSC-A
FSC-H
Viability - ZombieNIR
CD45 - APC/Fire750
TCRγδ - BV421
TCRβ - PE/Dazzle594
IL-22+ γδ T cells
IL-22 - APC
IL-5 - PE
IL-22+ ILC
Thy1.1 - BV605
Lineage - BV785
CD4 - BV650
CD8 - BUV805
IL-22+ CD4+T cells
E
IL-22+ ILC
IL-22+ γδ T cells
IL-22+ CD4+ T cells
IL-22+ ILC/g tissue
IL-22+ γδ T cells/g tissue
IL-22+ T cells/g tissue

Low Trp Diet Alters Bacterial AhR Agonists Production, Leading to Increased AL

We next speculated that nutritional support, designed to favor AhR agonist formation, could prevent AL. Trp-rich diets in WT and $IDO1^{-/-}$ or NOD/DQ8 mice[11] have been shown to favor the growth of *Lactobacillus*, a species metabolizing Trp into AhR agonists, thereby promoting intestinal homeostasis. Therefore, we hypothesized that lack or decrease of dietary Trp would increase AL rates. We fed WT mice with diets containing either low (0.1%) or high (1%) Trp for 28 days prior to surgery and up until POD5 (Figure 6*A*). We first assessed the capacity of low- and high-Trp diets to affect the preoperative fecal Trp metabolites profile. Targeted liquid chromatography-mass spectrometry (LC-MS) Trp metabolomics quantification focused on catabolic Trp derivatives, categorized as metabolites of the kynurenine, serotonin, or indole pathways (Figure 6*B*). As expected, fecal Trp concentration was higher in the high-Trp diet group (Figure 6*C*) and was associated with increased concentration of metabolites from the indole pathway, such as IAA, ILA, Trp, and indole (Figure 6*C*, Supplementary Table 6). Metabolites of the kynurenine (IDO) and serotonin (5-HT) pathways, such as kynurenine, kynurenic acid, xanthurenic acid, quinolinic acid, 3-OH-anthranilic acid, 3-OH-kynurenine and serotonin, 5-OH-tryptophan, 5-OH-indole acetic acid, melatonin, and N-acetyl-serotonin were also higher in the high-Trp diet in the healthy WT mice (Figure 6*C* and *D*, Supplementary Table 6). Finally, the kynurenine-to-Trp ratio, a marker for IDO1 enzyme activity, used as a proxy of inflammation, tended to be lower in the feces of the high-Trp diet-fed mice ($P = .06$) (Figure 6*C*). In consequence to the low Trp diet, we observed strongly impaired AH (Figure 6*E* and *F*), with most animals exhibiting abscess formation (ACS = 4), whereas high Trp diet-fed mice mostly presented with simple adhesions or were not affected at all (ACS 0–2) (Figure 6*E* and *F*).

Given that dietary Trp levels have been shown to affect murine fecal microbiome composition, we were wondering if there are also changes to the mucosal-associated microbiome under low- or high-Trp diets in the postoperative course. 16S rRNA sequencing of mucosal swabs were taken from a distant unaffected colonic part on POD5 to not interfere with the anastomosis itself. The colonic mucosal microbial composition of both low- and high-Trp diet-fed animals, constituted mostly of *Firmicutes*, *Bacteroidetes*, and *Proteobacteria* (Figure 6*G*), with minor differences in their relative proportion to the total biomass (P-adjusted > .05 for all taxa at phylum level). However, there was no difference in the mucosal-adherent microbial α-diversity of the mice that received the high-Trp diet compared with the mice fed the low-Trp diet ($P = .733$ and $P = .494$, for Shannon-index and observed richness, respectively) (Figure 6*J* and *K*). The treatment with high- or low-Trp diet could also not explain differences in amplicon sequence variant (ASV) community structure (β-diversity) for the unweighted UniFrac distance ($R^2 = .06$; $P = .405$) (Figure 5*H*). Interestingly, within the 17 mucosal swabs, 768 distinct ASVs were found. Of these, 52 ASVs differed significantly between the mice that received a high-Trp diet and the mice fed the low-Trp diet (Supplementary Table 8). At genus level, mice fed the high-Trp diet had higher relative abundance of taxa annotated to *Bifidobacterium* and *Clostridium sensu stricto* 1 than mice treated with low-Trp diet (log2fold change [FC] = 3.5 and 2.9, for *Bifidobacterium* and *Clostridium sensu stricto* 1, respectively; P-adjusted = .051, for both taxa (Figure 5*I*). Subsumed, although high- and low-Trp diets only moderately affect overall mucosal microbial richness and diversity, we found distinct changes in AhR-agonist generating genera towards a higher presence under high-Trp diets.

Supplementation With Indole-3-Carbinol Reverses Low Tryptophan-Induced AL

To finally validate the implication of low luminal levels of AhR agonists in the low-Trp diet-induced AL, we investigated whether low-Trp diet-fed mice can be rescued from increased AL rates by oral supplementation with I3C (Figure 7*A*). Mice were fed a low-Trp diet and were gavaged daily with I3C, an AhR agonist precursor endogenously metabolized into the active AhR agonists 3,3'-diindolylmethane (DIM) and indolo [3,2-b] carbazole (ICZ). Strikingly, I3C strongly reduced signs of AL, as manifested by the lower ACS (Figure 7*B* and *C*).

Taken together, our findings provide new evidence about the supportive role of the AhR/IL-22 axis in AH and offer potential measures (eg, perioperative IL-22 or dietary Trp supplementation) as supportive measures to prevent AL.

Discussion

AL is a serious complication following intestinal resection, and its etiology and pathophysiology are still

Figure 3. (***See previous page***). **Detection of IL-22 mRNA and IL-22 producing cells.** (*A*) Representative RNAscope fluorescent in situ hybridization of *IL-22* mRNA in naïve WT mice, and WT and $AhR^{-/-}$ mice 6 hours after anastomotic surgery. Nuclei were stained with DAPI (*blue*); *IL-22* mRNA signals are shown in *white or red* (in the merged images). Regions of interest (ROIs) with higher magnification are indicated by *white rectangles*. Scale bar from left to right: 200 μm, 20 μm, 5 μm. n = 2 mice per group. *Arrows* indicate *IL-22* mRNA. (*B*) Representative flow cytometry plots showing Gata3 (ILC2 marker) and RORγt (ILC3 marker) expression in anastomotic tissue of either WT or $AhR^{-/-}$ mice 12 hours after surgery. (*C*) Total ILC, ILC2, and ILC3 cell numbers per gram of anastomotic tissue. (*D*) Gating strategy used to identify IL-22 producing cells in anastomotic tissue of WT mice 12 hours post-surgery and after an additional 6 hours of IL-1β/IL-23 stimulation. Additional representative plots of IL-22 and either TCR$\gamma\delta$ ($\gamma\delta$ T cells), IL-5 (ILC), or CD4 ($CD4^+$ T cells) are shown for both WT and $AhR^{-/-}$ mice. (*E*) Numbers of IL-22-producing ILCs, $\gamma\delta$ T cells and $CD4^+$ T cells per gram of anastomotic tissue. (*B–E*) n = 3 mice per group. Bars showing mean ± SEM. Statistical significance was determined by Student's 2-tailed *t* test. *$P < .05$.

insufficiently characterized. Recent studies addressed the role of the gut microbiome in homeostasis as well as during inflammation and found evidence of a particular role in the latter. Microbiome changes (eg, an increase of bacteria with higher collagenolytic activity[12,27,28]) are associated with higher AL rates. Recently, Hajjar et al reported distinct

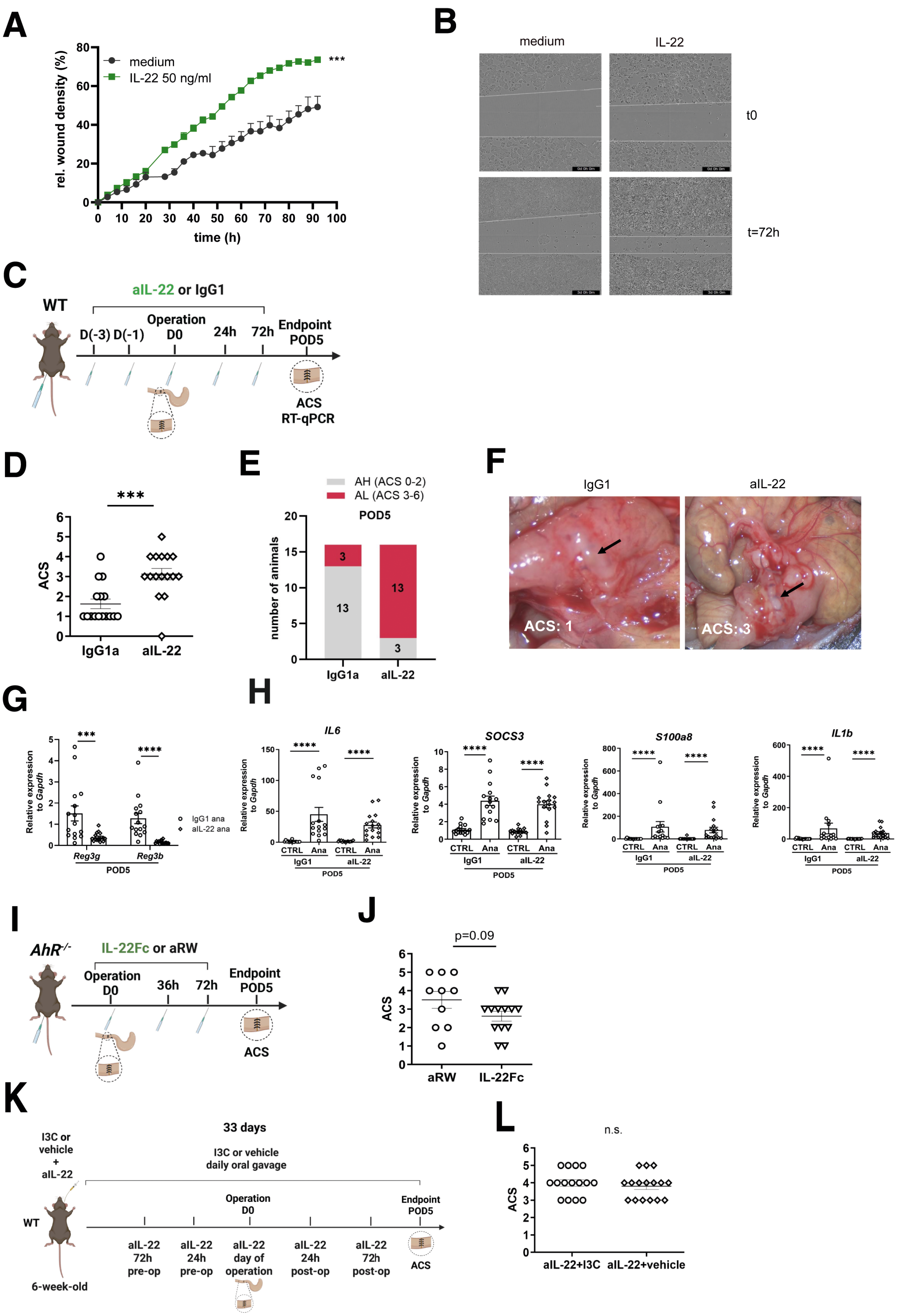

signatures of bacteria related to AL and revealed that this phenotype can be transferred to naïve mice by fecal microbiota transplantation, resulting in enhanced anastomotic inflammation.[13]

The gut microbiome composition depends on diets and, in turn, determines the availability of many bioactive metabolites relevant to intestinal homeostasis and inflammation. The metabolism of the essential amino acid Trp plays a fundamental role in microbiome-host crosstalk. Herein, we provide evidence about a causal role of reduced levels of gut luminal Trp metabolites, particularly AhR-activating indoles, in the pathogenesis of AL in male patients with CRC emphasized by mechanistic insights on the beneficial role of the AhR/IL-22 axis in AH in a murine AH model. First, among a list of 21 Trp metabolites, we associated lower preoperative fecal IAA levels with AL in male patients with CRC but not in the cohort containing males and females. This observation highlights sex as an important factor, coinciding with previous studies listing male sex as a risk factor for AL.[29]

Given that IAA is an AhR agonist, we proved that AhR is essential for proper AH by subjecting pan AhR deficient mice (*AhR*$^{-/-}$) displaying signs of AL already on POD1 and even worsening on POD5. Among several tested AhR-dependent target genes, only *IL-22* was strongly induced in anastomotic tissue, especially in the immediate postoperative phase, and fully depends on AhR presence. The early postoperative induction of IL-22 aligns with previous studies, showing that *IL-22* is immediately secreted within the first hours of inflammation.[30] Moreover, IL-22 prompts colonic mucosal epithelial cells to induce secretion of antimicrobial peptides (AMPs) and other molecules related to host protection,[22,31–33] findings we could partially confirm within *AhR*$^{-/-}$ mice showing a trend towards attenuated *Reg3b* and *Reg3g* gene expression compared with WT mice. Not unexpectedly, AMP expression was not entirely abolished by AhR deficiency, as the release of AMP by Paneth cells and resulting mucosal healing[34–36] was shown to depend on various factors apart from *IL-22*.[22,36] Furthermore, multiple groups have demonstrated that AhR acts with IL-22 in maintaining or restoring the epithelial barrier integrity and immunity through various innate immune cell types, such as ILC3, particularly the IL-22 producing NKp46^{+} RORγt^{+} ILC3 cells.[37,38] We confirmed the presence of these IL-22^{+} ILCs in the anastomotic tissue of WT mice, which were reduced in *AhR*$^{-/-}$ mice.

IL-22 is a highly relevant cytokine due to its ambivalence in inflammation, where it can be protective or pathogenic, depending on the experimental disease model, the involved tissue, and timing (immediate vs delayed).[30] In the colon of dextran sulfate sodium (DSS)-induced colitis mice, a preclinical model for IBD, mRNA levels of *IL-22* were elevated and seemed protective, as antagonism of IL-22 signaling worsened the disease score and impaired wound recovery.[31,39,40]

In agreement with these functions, our findings identify IL-22 as a crucial mediator of intestinal AH. Neutralization of IL-22 in WT mice impaired wound closure and led to AL, confirming that IL-22 signaling is indispensable for proper repair after intestinal surgery. Because IL-22 expression at the anastomotic site depends on AhR signaling, these findings position IL-22 as a downstream target of AhR-mediated regulation in intestinal AH. AhR agonism through IC3 failed to restore healing when IL-22 was neutralized, as both I3C- and vehicle-treated mice exhibited comparable leakage rates. This demonstrates that AhR-induced repair requires intact IL-22 signaling and that AhR acts upstream to regulate IL-22 production. These results suggest that AhR activation alone may be insufficient to drive mucosal healing in conditions where IL-22 signaling is compromised, such as chronic inflammation or microbial dysbiosis.[41]

Conversely, administration of recombinant IL-22Fc protein to *AhR*$^{-/-}$ mice only partially improved healing, suggesting a synergistic rather than unidirectional interaction between AhR and IL-22. Further pharmacological studies exploring IL-22Fc dosing, formulations, timing, and application route will be valuable to determine whether exogenous IL-22 can effectively compensate for impaired AhR activity.

It has been shown that AhR is broadly expressed in epithelial, stromal, and immune compartments, highlighting its potential to regulate both immune responses and epithelial regeneration. In our *AhR*$^{-/-}$ mice, IL-22 was markedly reduced, largely due to a dramatic decrease in ILC3 cells compared with WT mice. These data indicate that AhR is essential for maintaining IL-22 producing immune cells in our anastomotic model and suggest that impaired

Figure 4. (***See previous page***). **AhR-triggered IL-22 release supports AH.** (*A* and *B*) Scratch test on HT-29 treated with human recombinant IL-22: quantification of the relative wound density and representative images. (*C*) Scheme of the anastomotic surgery experiment in WT mice receiving pre- and postoperative intraperitoneal injections of neutralizing aIL-22 antibody (n = 16) or IgG1a control (n = 16). (*D*) ACS from anastomotic surgery on POD5 corresponding to (*C*). (*E*) aIL-22-treated WT mice showed more frequent AL (ACS ≥3; 13 of 16 mice) compared with control IgG1a-treated WT mice (3 of 16) on POD5. (*F*) Representative images from IgG1a and aIL-22-treated mice. (*G*) Transcript expression of *Reg3g* and *Reg3b*, 2 downstream targets of IL-22 in the anastomotic tissue of IgG1 and aIL-22-treated WT mice. (*H*) Transcript expression of *IL-6*, *IL-1β*, *S100a8*, and *Socs3* in the anastomotic and non-anastomotic control tissue of IgG1 and aIL-22-treated WT mice. (*I*) Scheme of the anastomotic surgery in *AhR*$^{-/-}$ mice that received postoperative intraperitoneal injections of recombinant IL-22Fc protein (n = 13) or aRW control (n = 10). (*J*) ACS from anastomotic surgery on POD5 corresponding to (*I*). (*K*) Scheme of the anastomotic surgery experiment in WT mice treated with aIL-22 antibody and I3C (n = 16) or vehicle control (n = 16). (*L*) ACS from anastomotic surgery on POD5 corresponding to (*K*). Data represents pooled results of 2 independent experiments. Each dot represents a mouse, with bars showing mean ± SEM. Statistical significance was determined by unpaired *t*-test (*A*, *G* and *J*), Mann-Whitney *U* test (*D* and *L*), or 2-way ANOVA with Tukey's post hoc test (*H*). *$P < .05$; ***$P < .001$; ****$P < .0001$. aIL-22, antiI-IL-22.

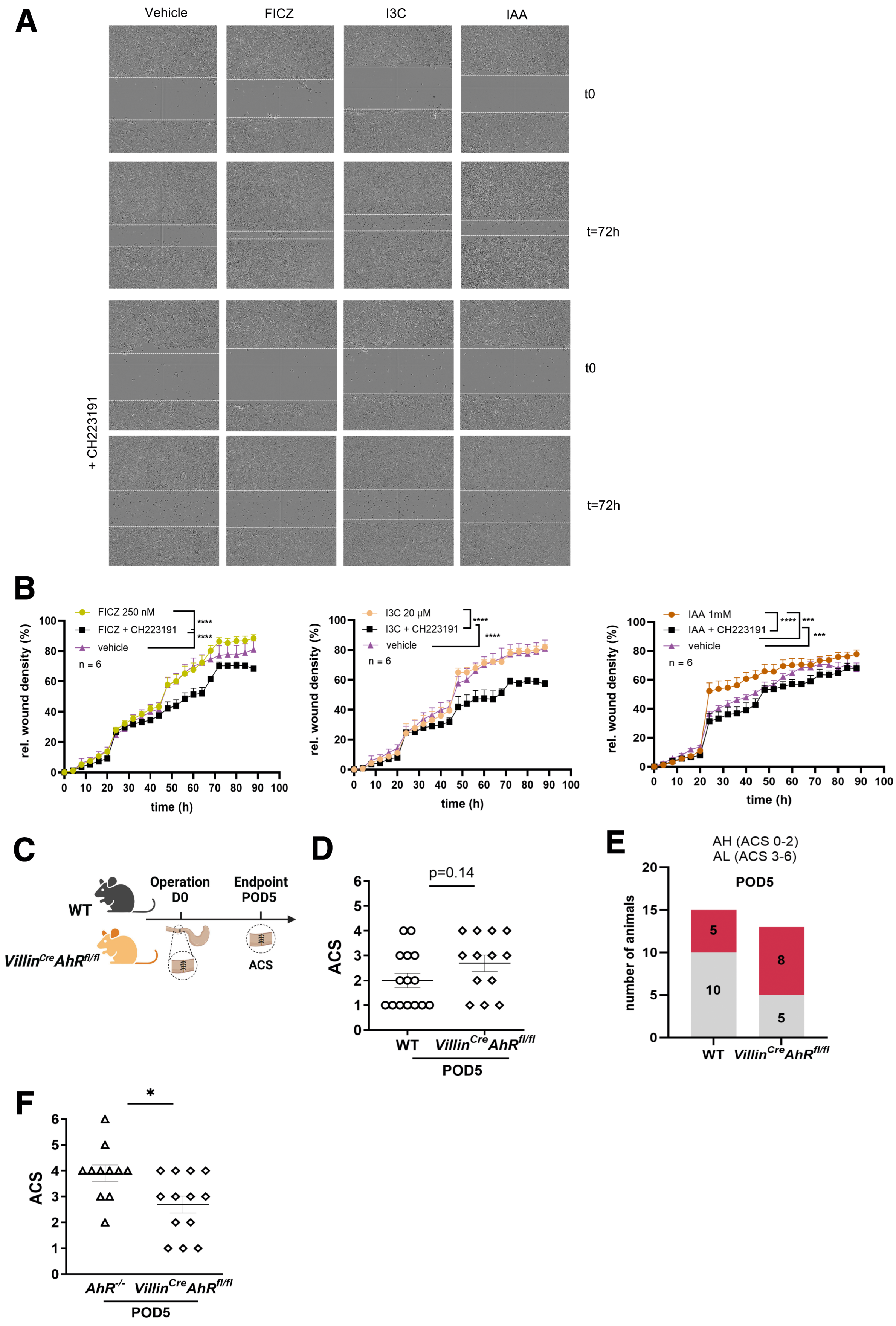
A
Vehicle
FICZ
I3C
IAA
t0
t=72h
t0
t=72h
+ CH223191
B
FICZ 250 nM
FICZ + CH223191
vehicle
n = 6
I3C 20 µM
I3C + CH223191
vehicle
n = 6
IAA 1mM
IAA + CH223191
vehicle
n = 6
rel. wound density (%)
time (h)
C
WT
VillinCreAhRfl/fl
Operation D0
Endpoint POD5
ACS
D
p=0.14
ACS
WT
VillinCreAhRfl/fl
POD5
E
AH (ACS 0-2)
AL (ACS 3-6)
POD5
number of animals
WT
VillinCreAhRfl/fl
F
ACS
AhR-/-
VillinCreAhRfl/fl
POD5

IL-22 signaling underlies the anastomotic healing disturbances observed in the *AhR*$^{-/-}$ mice.

The AhR is broadly expressed in various intestinal cells.[24,26,42–44] Besides its effect in immune cells, which appears to be the main driver of AH, AhR signaling was described being supportive for epithelial healing by a direct action on epithelial cells.[45,46] Therefore, we also investigated whether epithelial AhR contributes to AH. ACS scores of *Villin*^{Cre}*Ahr*$^{fl/fl}$ mice did only moderately, but not significantly, differ from their Cre-negative AhR$^{fl/fl}$ littermates. The missing difference indicates that immune cells, particularly ILC3s, are key for the AhR-mediated beneficial action in intestinal "transmural" healing, a process that requires more cells than the epithelium. Nevertheless, our findings do not totally exclude a role of epithelial AhR action in AH, as Metidji et al showed that IL-22 expression is increased in *Villin*^{Cre}*Ahr*$^{fl/fl}$ mice compared with WT mice. This might be due to the elevated levels of AhR agonists that are available to the mucosal immune cells as they were not bound anymore by epithelial AhR.[44] Therefore, AH in *Villin*^{Cre}*Ahr*$^{fl/fl}$ mice might be partially supported by increased levels of IL-22, and this could explain the modest but non-significant increase of mice with elevated ACS scores in the *Villin*^{Cre}*Ahr*$^{fl/fl}$ group. This explanation is consistent with the concept that epithelial restoration depends on crosstalk between immune-derived cytokines and epithelial responsiveness.[47]

Given the beneficial effect of AhR presence on AH, we studied a nutrition-based AhR agonistic concept as a potentially clinically relevant approach to support AH. Many experimental models have been exploited to test hypotheses regarding dietary-induced shifts in the microbiome. For instance, a high-fat/low-fiber diet, described as a Western diet, creates a dysbiotic microbial community, which increases the AL rate.[48] Moreover, an enriched Trp diet has displayed decreased immunopathology in nonobese diabetic (NOD) mice expressing the DQ8 celiac disease susceptibility gene (*NOD/DQ8*) after exposure to gluten.[11] Pre- and perioperative dietary interventions have been a major consideration among surgeons, and one human study has shown an association between higher habitual preoperative dietary fiber intake and lower risk of postoperative complications in patients with CRC.[49] However, although a healing supportive effect has been suggested, no human clinical trials have been conducted yet.

In line with previous findings reporting increased fecal levels of indole-producing bacteria (eg, *Lactobacillus spp*) and enrichment of AhR agonists after 3-weeks of high-Trp diet feeding to mice,[11] we detected increased fecal levels of AhR agonists in high-Trp diet-fed WT mice. These agonists included indoles and Trp catabolites generated by the host cells from the IDO and 5-HT pathways, which in the low-Trp diet led to disturbed healing, as shown by the ACS. In contrast to the findings of Lamas et al,[11] we also observed increased levels of kynurenine. We can speculate that this difference is due to divergent housing conditions in the animal facilities, other genetic backgrounds and microbiomes, or durations of the feeding regimes. However, as kynurenine has been shown to act as an AhR agonist,[50] its higher presence might promote AhR signaling. As the low-Trp diet promoted AL, strict monitoring and perioperative control of dietary Trp contents in patients scheduled for surgery might present a promising future clinical intervention in the context of AL prevention. Although this study focused on the importance of the microbiome in AH/AL through its production of indoles, it is worth mentioning that intestinal host cells can also metabolize Trp. Different dietary Trp concentration affected the fecal metabolite profile of the mice fecal samples but did not affect significantly the colonic mucosal microbiome. Noteworthy, in a small human crossover study in healthy subjects, oral Trp supplementation showed only modest alterations in fecal microbiome. In particular, the authors reported that supplementation of healthy individuals with oral L-Trp affected duodenal rather than colonic fecal Trp metabolites concentrations as readout through AhR activity assays, leaving the conclusion that Trp is mainly catabolized in duodenum lumen.[51] Future work should demonstrate these local differences for patients subjected to colonic surgery.

Furthermore, previous studies reported that losing the fragile equilibrium between host cells and gut microbiota leads to various diseases.[14] Interestingly, the IDO and 5-HT pathways often display higher activation than the indole pathway in multiple intestinal and metabolic diseases.[52,53] This pathway shift might either occur due to the impaired ability of the gut microbiota to metabolize Trp into AhR or their direct or indirect (via their effect on the host cells) influence on the other 2 pathways. However, although indoles improve gut and overall human metabolic health, partly by acting as anti-inflammatory molecules via AhR/IL-22 activation,[16,17,54] there are few indole derivatives, such as indole, which is converted into indoxyl sulfate in the liver and is positively correlated with chronic kidney disease.[55,56] Therefore, exploiting quantitative methods to determine

Figure 5. (***See previous page***). **Epithelial-restricted AhR activation in cell migration and AH.** (*A*) Representative images from a scratch test on HT-29 cells treated with either I3C, IAA, or FICZ (*upper panel*), or one of these substances and AhR antagonist CH223191 (*bottom panel*) at the day of the scratch ($t = 0$) or three days later ($t = 72$ hours). (*B*) Quantification of the relative wound density (%) of conditions in (*A*) over the course of 88 hours in 4-hour intervals. Results show mean ± SD ($n = 6$). (*C*) Scheme of the anastomotic surgery experiment in *Villin*^{Cre}*AhR*$^{fl/fl}$ mice. (*D* and *E*) ACS and frequency of AL occurrence from the anastomotic surgery in *Villin*^{Cre}*AhR*$^{fl/fl}$ mice ($n = 13$) and their WT littermates ($n = 15$). (*F*) Comparison of ACS between *Villin*^{Cre}*AhR*$^{fl/fl}$ and full *AhR*$^{-/-}$ mice. Each dot represents a mouse, with bars showing mean ± SEM. Statistical significance was determined by 2-way ANOVA with Dunnett's multiple comparisons test (*B*), and Mann-Whitney *U* test (*D* and *F*). *$P < .05$; **$P < .01$; ****$P < .0001$.

the concentration of indole and its derivatives in every clinical condition is important for the interpretation of the side effects.

In summary, the present study demonstrates a critical link between AhR and IL-22 signaling pathways, provides new insight into intestinal transmural AH, and suggests

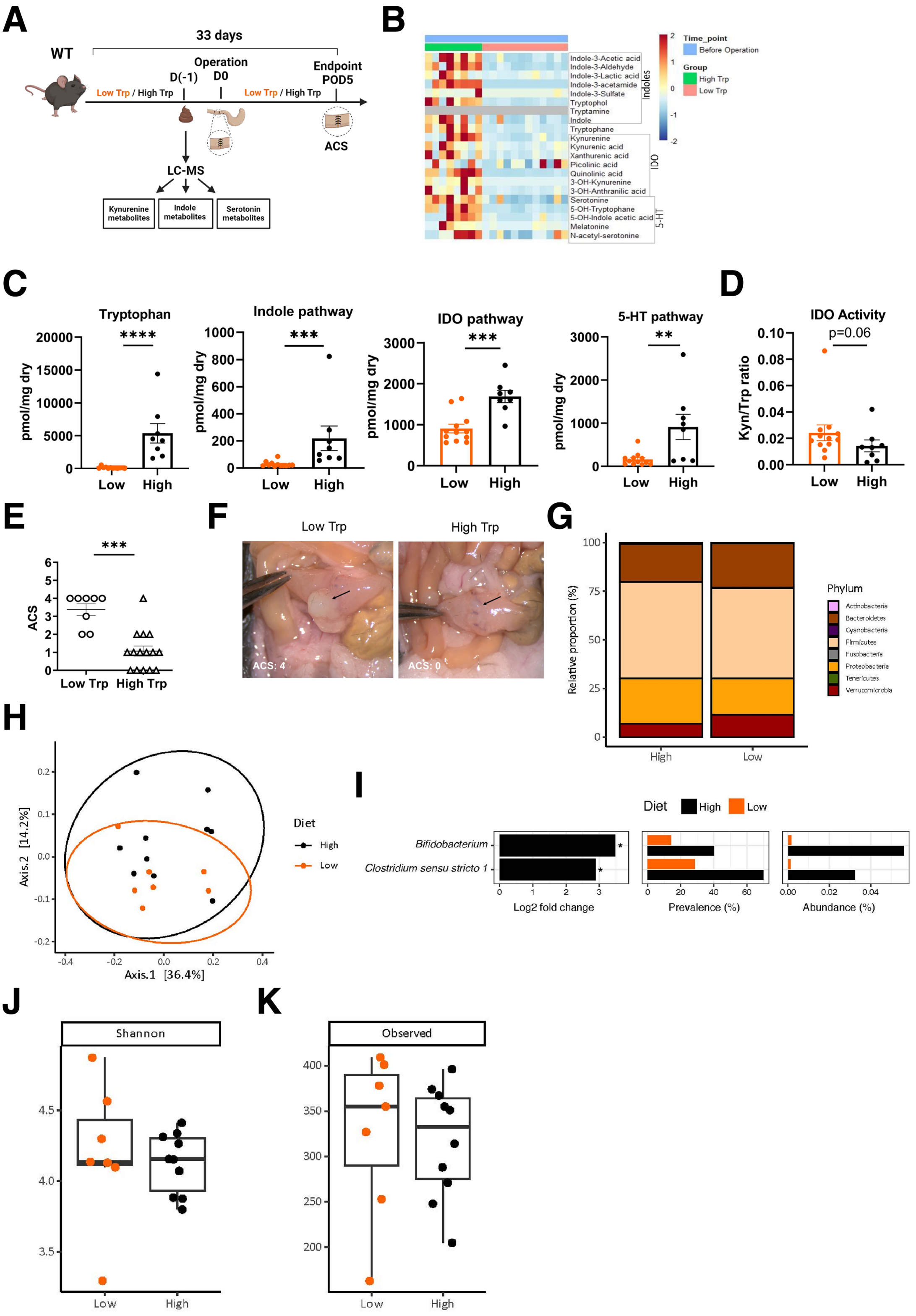

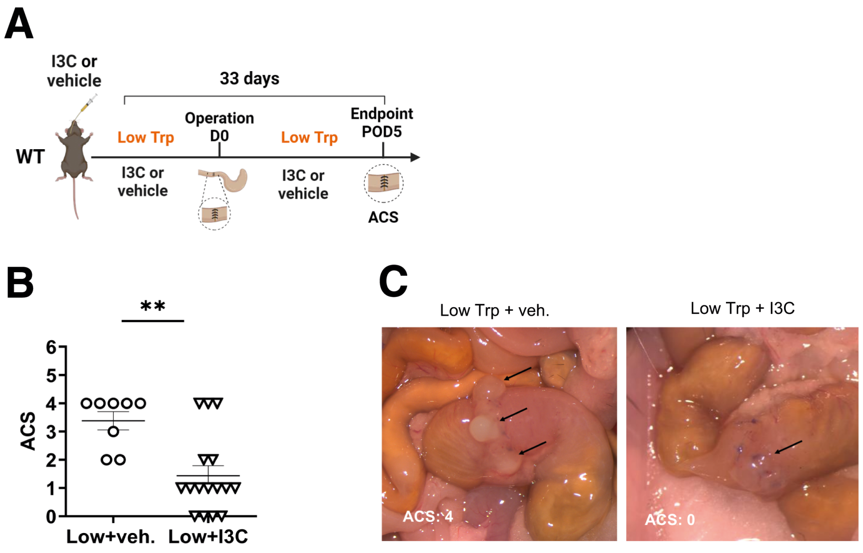

Figure 7. Supplementation with I3C reverses low Trp-induced AL. (*A*) Scheme of the I3C/low-Trp diet experiments. WT mice receiving a low-Trp diet were gavaged daily with I3C or vehicle prior- and post-surgery. (*B*) ACS on POD5 from operated WT mice after low-Trp diet and vehicle (n = 8) or I3C (n = 16) supplementation. (*C*) Representative images. Each dot represents a mouse, with bars showing mean ± SEM. Statistical significance was determined by Mann-Whitney *U* test. $^{**}P < .01$.

potential therapeutic strategies to enhance AH. Supplementation of AhR agonists, recombinant IL-22Fc protein, or preoperative nutritional changes towards diets enriched in Trp seemed to promote physiological AhR activation, which positively affected AH. From a translational perspective, targeting the AhR/IL-22 axis may be particularly relevant for colorectal surgery, where AL remains a major cause of postoperative morbidity and mortality.

Limitations

This study has few limitations. First, IL-22Fc showed only a trend in improving the impaired AH in $AhR^{-/-}$ mice. More extensive pharmacological studies are needed to find optimized regimes for improving AH by IL-22 application. Secondly, it remains unclear which cells, apart from epithelial cells, contribute to AhR signaling in AH. However, due to the broad AhR expression in various cell types,[42–44] a concerted action from multiple cell types seems likely. Furthermore, although we set our endpoint on POD5 to enable observation of the healing phenotype, this time point in the wound healing process largely involves tissue remodeling.[57] Thus, it is defined by the secretion of molecules associated with extracellular remodeling and wound healing, and not cytokines, which are rather expressed in the immediate inflammatory phase of healing.

Materials and Methods

Human Study

Patients with colorectal cancer scheduled to undergo surgical resection with anastomosis were recruited in the REVEAL trial, a multicenter, prospective, observational study.[20] Patients with AL and AH were matched to achieve a ~1:2 case-control cohort based on age, sex, body mass index (BMI), tumor location, and neoadjuvant therapy. Participants provided written informed consent. The study protocol followed principles of Good Clinical Practice and was conducted in accordance with the Declaration of Helsinki. The study was approved by the Medical Ethical Committee of the Maastricht University Medical Centre (Maastricht, the Netherlands) and registered at ClinicalTrials.gov (ClinicalTrials.gov, Number: NCT02347735). Feces were collected by the patients on the day prior to surgery and immediately stored at the −20°C freezers of the local hospitals. When patients' recruitment was completed, all samples were transferred

Figure 6. (***See previous page***). **Low-Trp diet alters bacterial AhR ligand production leading to increased AL.** (*A*) Scheme of Trp diet experiments. (*B*) Heatmap of Trp metabolites in preoperative fecal samples of WT mice fed a low-Trp (n = 12) or high-Trp (n = 8) diet, depicting the feces in the high-Trp group having higher concentrations of all the metabolites that are produced from the 3 Trp metabolic pathways compared with the feces from the low-Trp group, where they are significantly reduced. (*C*) Quantification of Trp and total metabolites from the indole, IDO, and 5-HT pathways. (*D*) IDO activity. (*E*) ACS on POD5 from operated WT mice after low- (n = 8) or high-Trp (n = 15) diets. (*F*) Representative images. (*G*) Mucosal-adherent relative abundance at the phylum level for mice fed the low Trp diet (n = 7) or the high-Trp (n = 10) diet. (*H*) PCoA for bacterial *16S rRNA* gene sequences based on unweighted UniFrac distance in the mucosal swabs of mice fed the low Trp diet (n = 7) or the high-Trp (n = 10) diet. (*I*) Relative abundance of taxa annotated to *Bifidobacterium* and *Clostridium sensu stricto 1*. (*J*) Alpha diversity of mucosal-adherent microbiota of WT mice fed a low-Trp diet (n = 7) or a high-Trp diet (n = 10), shown by the Shannon index and (*K*) the observed richness. Each dot represents a mouse, with bars showing mean ± SEM. Statistical significance was determined by Mann-Whitney *U* test (*C* and *E*: indoles, tryptophan, *D*) or unpaired *t* test (*C*: IDO pathway, 5-HT pathway). For differences in microbial composition, all statistical analyses were performed in R. PERMANOVA was applied using the vegan Adonis function on distance matrices. Differences in genus relative abundances between groups were found using the DESeq method. $^{**}P < .01$; $^{***}P < .001$; $^{****}P < .0001$.

to Amsterdam Medical Centers, location AMC, Amsterdam, The Netherlands, and kept for permanent storage at −80°C.

Animals

Eight-to-ten-week-old male and female $AhR^{-/-}$ mice and their WT littermates on the C57BL/6JRccHsd background were used. Female mice were purchased either from Janvier-Labs (C57BL/6J) or Envigo (RMS B.V.; C57BL/6JRccHsd) and allowed to acclimatize for 1 week before surgery. Only females were purchased to reduce fights and minimize the number of animals according to the 3R rules. Littermates of the $AhR^{-/-}$ and $Villin^{Cre}Ahr^{fl/fl}$ mice were cohoused after weaning and throughout the experiment. All the mice were maintained in individually ventilated cages under specific pathogen-free (SPF) conditions with standard rodent food, unless otherwise stated in the text, and tap water ad libitum. The mice were randomly re-caged into the different groups in each experiment, and they were randomized within blocks in each experiment. All experiments in Bonn were performed according to the federal law for animal protection and were approved by the State Agency for Nature, Environment and Consumer Protection (LANUV) (#81-02.04.2021.A179). All animal experiments in Amsterdam were approved by the Amsterdam University Medical Center Animal Experiments Committee, and the protocol complied with the Dutch Animal Experimental Act (DMO20-11104-1-11).

Surgery

Prior to surgery, mice received a subcutaneous injection of analgesic 15 minutes before the operation: tramadol (30 mg/kg body weight; Grünenthal) at the UKB; baytril (5 mg/kg; Bayer) and buprenorphine (0.1 mg/kg; Ecuphar S.A.) at the AMC. The mice underwent general anesthesia with isoflurane (2%–4%). The abdomen was shaved, the skin was disinfected, and a protective salve was placed on the eyes. The mice were placed on a heating pad where they were maintained on pressurized air (2 L/min) at the UKB or oxygen at the AMC and isoflurane (1.5%–2%). After a 2-cm median laparotomy, the cecum and proximal colon were identified, mobilized outside of the abdominal cavity, and placed onto sterile saline infused gauze. The proximal colon (approximately 1 cm from the cecum) was transected, and an end-to-end anastomosis was constructed with 8 interrupted sutures, using 8-0 Vicryl sutures (Ethicon; Cat no. V547G). The integrity of the anastomosis was tested by light pressure of the area with cotton swabs. After creation of the anastomosis, the colon was placed back in the abdominal cavity and warm NaCl was added. The peritoneum was closed with 5-0 Vicryl and the skin sutured with the 5-0 silk (C-0762067). Drinking water was supplemented with tramadol (1 mg/mL; Aliud Pharma) to assist on analgesia (UKB).

Anastomotic Complication Score

The AH was evaluated using the previously described ACS.[21,57,58] A score between 0 and 2 was considered regular AH, with no signs of AL, whereas a score ≥3 is regarded as AL (Supplementary Table 5). The operations and assessment of anastomosis were performed blindly by the researchers.

Neutralizing IL-22 Antibody and I3C Administration

For the AhR activation and IL-22 depletion study, 6-week-old female C57BL/6J mice (n = 16) were given daily I3C (100 μL of 25 mg/kg body weight, Sigma-Aldrich, Cat. No. I7256; n = 16), or saline via oral gavage for 3 weeks prior to surgery until sacrifice. The mice were injected with 150 μg/100 μL of anti-IL-22 neutralizing antibody (clone 8E11; Genentech) at the following time points: 72 hours and 24 hours prior to surgery, immediately after surgery, and 24 hours and 72 hours after surgery. All mice were euthanized on POD 5.

Custom TrP Mouse Diet and I3C treatment

For the diet studies, 5-week-old female C57BL/6J mice (n = 16 per diet) were fed either customized purified diet S0016-E080 (0.1% Trp AA diet) or S0016-E084 (1% Trp AA diet; SSNIFF.de) (Supplementary Table 7), for 3 weeks prior to surgery until sacrifice. In the S0016-E084 group, one mouse died before the operation, and was thus removed from the analysis. An additional set of mice fed with the low tryptophan diet S0016-E080 (0.1% Trp AA diet) and received daily oral gavage of I3C (100 μL of 25 mg/kg body weight, Sigma-Aldrich, Cat. No. I7256; n = 16), or saline (n = 8) for 3 weeks until surgery and subsequent sacrifice.

IL-22Fc Administration and Neutralizing IL-22 Antibody Administration

Male and female $AhR^{-/-}$ mice were injected intraperitoneally with 50 μg/100 μL of either IL-22Fc (n = 13) or anti-ragweed isotype control (n = 13) (Genentech) immediately after surgery, 36 hours, and 72 hours after surgery. In the control group, one mouse did not get administered with enough isotype; of the remaining 12, 2 mice died before the end of the experiment, and thus, all 3 were removed from the analysis.

Female C57BL/6J mice (n = 16; 2 independent experiments) were injected with 150 μg/100 μL of either anti-IL-22 nAb (clone 8E11; Genentech) or IgG1 control (Genentech) at the following time points: 72 hours and 24 hours prior to surgery, immediately after surgery, and 24 hours and 72 hours after surgery. All mice were euthanized on POD 5.

Cell Isolation for Flow Cytometry

Immune cells were isolated from the large intestinal lamina propria. Mice were scarified by cervical dislocation, and the anastomotic affected area was surgically removed. The tissue was cut open and washed in RPMI with 3% fetal bovine serum (FBS) to remove feces, patted dry on a paper towel, and then weighed. The tissue was

added to a flask with 10 mL of strip media (RPMI with 3% FBS, 5 mM EDTA, and 1 mM dithiothreitol [DTT]), then incubated for 20 minutes at 37°C on a magnetic stirrer. The tissue was then added to a falcon with 10 mL of shake media (RPMI with 2mM EDTA), then shaken vigorously for 30 seconds. The shake media was replaced, the tissue was shaken 2 more times, and then the tissue was washed with phosphate-buffered saline (PBS). The tissue was then added to a beaker with 1 ml of digest media (RPMI with 0.1 mg/mL Liberase and 0.5 mg/mL DNase) and finely minced with scissors. Another 2 mL of digest media was added and incubated for 27 minutes at 37°C on a magnetic stirrer. The digestion was stopped with 6 mL of RPMI with 3% FBS and digested tissue passed through a 70-μm filter into falcon tubes to generate single-cell suspensions. Tubes were centrifuged at 400 × g for 5 minutes, and supernatant discarded. Cell pellets were resuspended in RPMI with 3% FBS then passed through a 40-μm cell strainer. Single-cell suspensions were centrifuged again, then resuspended in RPMI 10% until further flow cytometry analysis.

Flow Cytometry

Single-cell suspensions were generated from the lamina propria of anastomotic tissue as described above. For transcription factor staining, dead cells were stained with Zombie NIR (Biolegend). Fc receptors were blocked with anti CD16/CD32 (*InVivo*MAb, BioXCell) before staining of surface antigens for 45 minutes at room temperature. Cells were fixed and permeabilized using a FoxP3/Transcription Factor Staining Buffer Set (eBioscience), then stained with intracellular antibodies for 1 hour at room temperature.

For cytokine staining, cells were incubated for 6 hours in Iscove's Modified Dulbecco's Medium (IMDM) with 10% FBS, 10 ng/mL IL-23, and 10 ng/mL IL-1β. Dead cells were stained with Zombie NIR (Biolegend), and Fc receptors blocked with anti CD16/CD32. Cells were stained for surface antigens for 45 minutes at room temperature, fixed in 2% formaldehyde for 20 minutes, then permeabilized with 0.01% NP-40 for 4 minutes. After washing, intracellular antigens were stained for 1 hour at room temperature. Data was acquired on a Sony ID7000 (Sony Biotechnology) and analysis performed using FlowJo software (v10.9, Treestar).

Fluorescent in Situ Hybridization (RNAscope)

RNAscope Multiplex Fluorescent v2 Assay (ACD, Bio-Techne) was performed according to the manufacturer's instructions with minor modifications. Cryosectioned tissue sections (12 μm) were briefly air-dried at room temperature and then dried further at 40°C. They were then post-fixed in 4% paraformaldehyde (PFA), dehydrated using an ascending ethanol series (50%, 70%, 100%) and air-dried. Sections were then treated with hydrogen peroxide (hydrogen peroxide solution), subjected to target retrieval (RNAscope Target Retrieval Reagent [10×]) at 100°C, and digested with Protease III for 15 minutes at 40°C. Probes were hybridized for 2 hours at 40°C in a HybEZ oven (ACD, Bio-Techne). After stringent washing steps Wash buffer (RNAscope Wash Buffer), amplification steps (AMP1–3) were performed sequentially at 40°C. Channel-specific horseradish peroxidase (HRP)-based signal amplification was carried out using RNAscope Multiplex FL v2 HRP reagents, followed by tyramide signal amplification (TSA) with Cy5 fluorophore (TSA Vivid dyes, 1:3000 in TSA buffer), and HRP blocking. Nuclei were counterstained with 4′,6-diamidino-2-phenylindole (DAPI) for 1 to 2 minutes. Unless stated otherwise, all reagents for the RNAscope assay were obtained from ACD Bio-Techne. Slides were coverslipped with PolyMount (Polysciences) and stored at 4°C in the dark until imaging. Spinning-disk confocal microscopy (Visitron Visiscope, Visitrom Systems) was performed using standard excitation and emission settings for Cy5 and DAPI. Images were processed with VisiView (Version 6.0.0.37, Visitrom Systems) and Fiji (ImageJ2 - Verion: 2.14.0/1.54g).

Scratch Wound Assay

IncuCyte live cell imaging system (Sartorius) was used to capture HT29 cell proliferation and migration after wounding, in phase contrast every 4 hours at 4× magnification. The cells were seeded in an IncuCyte Imagelock 96-well plate (Sartorius) at a density of 5 × 10^4 cells per well in 100 μL of Dulbecco's Modified Eagle's Medium (DMEM) high glucose (4.5 g/L) (Capricorn, Cat. No. DMEM-HPXA). After 2 days, the IncuCyte 96-well WoundMaker was used to create a uniform scratch in every well. Stimulation was performed with I3C (20 μM; Sigma-Aldrich, Cat. No. I7256), FICZ (250 ng/mL; Sigma-Aldrich, Cat. No. SML1489-1MG), IAA (100 μM; Sigma-Aldrich, Cat. No. I2886-5G) with or without CH-223191 (10 μM; Sigma-Aldrich, Cat. No. C8124-5MG) and recombinant human IL-22 (50 ng/mL Peprotech, Cat. No. 200-22-2UG) for 88 hours and measured on a real-time imaging system (IncuCyte). DMEM/10% FBS/1% penicillin/streptomycin (P/S), the I3C and IL-22 vehicle, and dimethyl sulfoxide (DMSO), the FICZ vehicle, were used as the negative controls. The relative wound density (%), which measures the spatial cell density in the scratched area relative to the cell density outside of the area at every time point, was calculated using the Scratch Wound analysis of the IncuCyteZOOM software (Sartorius).[59]

Quantification of Fecal Trp Metabolites by Liquid Chromatography Coupled With High-Resolution Mass Spectrometry

Trp metabolite concentrations in the available preoperative fecal samples from the mouse diet study (high Trp group; n = 8, low Trp group; n = 12), and the human preoperative stool samples from the REVEAL study (AH; n = 33, AL; n = 19) were determined by liquid chromatography coupled with high-resolution mass spectrometry (LC-HRMS) as previously described.[60] IDO activity was determined by the kynurenine-to-Trp ratio.[61]

RNA Isolation

Total RNA was isolated from colon samples using the RNeasy mini kit (Qiagen; Cat. no. 74106) according to the manufacturer's instructions. The tissues were homogenized in a Precellys apparatus (30 seconds, 6100 rpm; Bertin Technologies) and kept on ice throughout the extraction process. RNA concentrations were measured in NanoDrop 1000 spectrophotometer (Thermo Scientific).

Gene Expression Analysis Using Quantitative Real-time Polymerase Chain Reaction

Complementary DNA (cDNA) was synthesized from 1 μg RNA using the High-Capacity cDNA Reverse Transcription Kit (Applied Biosystems; Cat. no. 4368813). Gene expression was quantified by quantitative reverse transcription-polymerase chain reaction (RT-qPCR) performed on a QuantStudio 5 System (Thermo Scientific), using Power SYBR Green PCR Master Mix (Thermo Scientific; Cat. no. #4367659) with the primers listed in Supplementary Table 9. Messenger RNA (mRNA) expression was determined with the $2^{-\Delta\Delta CT}$ method normalizing to mouse *Gaphd* gene expression and using WT naïve colon group as a calibrator.

DNA Extraction

DNA was extracted from mucosal-adherent swabs, which were stored in collection and preservation medium (COPAN; Cat No 201CS01), using a combination of repeated bead-beating and the Maxwell RSC Blood DNA kit (Promega Leiden, the Netherlands) with Stool Transport and Recovery (STAR) buffer (Roche). The samples were thawed at room temperature and centrifuged for 10 minutes at 14,000 rpm (4°C). The supernatant was removed, and 100 μL of the precipitant was mixed with 300 μL STAR buffer and 30 μL proteinase K. The total volume, including the swab stick, was transferred to bead tubes, shaken for 20 minutes at 1000 rpm (56°C), and homogenized with the bead beater for 1 minute at 5 m/sec. After 5 minutes of incubation at 95°C, the samples were centrifuged for 5 minutes at 12,700 rpm (4°C), and the supernatant was transferred to a new nuclease-free tube. The latter steps were repeated; 200 μL STAR buffer was added in the bead tubes, homogenized for 1 minute at 5 m/sec, centrifuged for 5 minutes at 12,700 rpm (4°C), and transferred to the respective nuclease-free tube. From the final volume, 250 μL was transferred to the Maxwell tubes, filled with 60 μL of elution buffer (ie, nuclease-free water).

16S rRNA Gene Sequencing

16S rRNA gene amplicons were generated using a single-step PCR protocol targeting the V3–V4 region.[62] The PCR reaction in 20 μL was performed with following thermocycling conditions: initial denaturation at 94°C followed by 25 cycles denaturation (45 seconds at 94°C), annealing (60 seconds at 52°C), extension (90 seconds at 72°C), and final extension at 72°C for 10 minutes. Forward and reverse reads were truncated to 240 and 210 bases, respectively, and merged using USEARCH; merged reads that did not pass the Illumina chastity filter, had an expected error rate higher than 2, or were shorter than 380 bases were filtered. ASVs were inferred for each sample individually with a minimum abundance of 4 reads. Unfiltered reads were then mapped against the collective ASV set to determine the abundances. Taxonomy was assigned using SILVA 16S ribosomal database V132.[63]

Statistical Analysis

GraphPad Prism version 9.5.1 was used for all analyses and preparation of graphs. SPSS version 28.0.1.1 was used for the patient cohort. Results are expressed as mean ± standard error of the mean (SEM). For comparisons between 2 groups, a 2-tailed Student's *t*-test for unpaired data or a nonparametric Mann-Whitney test was used. Two-way analysis of variance (ANOVA) and post hoc Tukey test were used for comparisons between 4 groups, and analyses were adjusted for multiple comparisons. Pearson χ^2 was used for the patient characteristics. Normal distribution was determined by the D'Agustino Pearson omnibus normality test, Shapiro-Wilk test, and Kolmogorov-Smirnov test with Dallal-Wilkinson-Lillie correction. For datasets that failed normality tests, a nonparametric Mann-Whitney test was used to analyze statistical differences. Animals were randomly assigned to experimental groups. The sample size for the mice experiments was calculated using G*Power based on α error probability of .05 and power (1-β error probability) of 0.80. For the 16S rRNA sequencing experiment, all statistical analyses were performed with R (v. 4.3.2, RStudio v. 2023.12.1+402), using the phyloseq (v. 1.46.0), vegan (v. 2.6.4) and DESeq2 (v. 1.42.1) packages. Alpha diversity was examined at observed species richness, and Shannon index level and microbial composition (β-diversity) was assessed using principal coordinate analysis (PCoA) at ASV level based on unweighted UniFrac distance. Nonparametric permutational multivariate analysis of variance (PERMANOVA) was applied using the vegan Adonis function on distance matrices. Differences in ASV and genus relative abundances between groups were found using the DESeq method. For all statistical tests, differences corresponding to $P < .05$ were considered significant. For genera exhibiting significant differences in relative abundance between groups, prevalence was determined as the proportion of samples in which each taxon was detected within a group, whereas relative abundance was computed as the proportion of each taxon relative to the total microbial community within individual samples.

Supplementary Material

Note: To access the supplementary material accompanying this article, visit the full text version at https://doi.org/10.1016/j.jcmgh.2026.101756.

References

1. Lee JA, Chico TJA, Renshaw SA. The triune of intestinal microbiome, genetics and inflammatory status and its impact on the healing of lower gastrointestinal anastomoses. FEBS J 2018;285:1212–1225.
2. Nandakumar G, Stein SL, Michelassi F. Anastomoses of the lower gastrointestinal tract. Nat Rev Gastroenterol Hepatol 2009;6:709–716.
3. Ochs V, Taha-Mehlitz S, Lavanchy JL, et al. Development of a clinical prediction model for anastomotic leakage in colorectal surgery. JAMA Netw Open 2025;8:e2538267.
4. McDermott FD, Heeney A, Kelly ME, et al. Systematic review of preoperative, intraoperative and postoperative risk factors for colorectal anastomotic leaks. Br J Surg 2015;102:462–479.
5. Sripathi S, Khan MI, Patel N, et al. Factors contributing to anastomotic leakage following colorectal surgery: why, when, and who leaks? Cureus 2022;14:e29964.
6. Hammond J, Lim S, Wan Y, Gao X, Patkar A. The burden of gastrointestinal anastomotic leaks: an evaluation of clinical and economic outcomes. J Gastrointest Surg 2014;18:1176–1185.
7. Lamas B, Michel ML, Waldschmitt N, et al. Card9 mediates susceptibility to intestinal pathogens through microbiota modulation and control of bacterial virulence. Gut 2018;67:1836–1844.
8. Blacher E, Levy M, Tatirovsky E, Elinav E. Microbiome-modulated metabolites at the interface of host immunity. J Immunol 2017;198:572–580.
9. Michaudel C, Danne C, Agus A, et al. Rewiring the altered tryptophan metabolism as a novel therapeutic strategy in inflammatory bowel diseases. Gut 2023;72:1296–1307.
10. Meynier M, Baudu E, Rolhion N, et al. AhR/IL-22 pathway as new target for the treatment of post-infectious irritable bowel syndrome symptoms. Gut Microbes 2022;14:2022997.
11. Lamas B, Hernandez-Galan L, Galipeau HJ, et al. Aryl hydrocarbon receptor ligand production by the gut microbiota is decreased in celiac disease leading to intestinal inflammation. Sci Transl Med 2020;12:eaba0624.
12. Shogan BD, Belogortseva N, Luong PM, et al. Collagen degradation and MMP9 activation by Enterococcus faecalis contribute to intestinal anastomotic leak. Sci Transl Med 2015;7:286ra68.
13. Hajjar R, Gonzalez E, Fragoso G, et al. Gut microbiota influence anastomotic healing in colorectal cancer surgery through modulation of mucosal proinflammatory cytokines. Gut 2023;72:1143–1154.
14. Agus A, Planchais J, Sokol H. Gut microbiota regulation of tryptophan metabolism in health and disease. Cell Host Microbe 2018;23:716–724.
15. Ghiboub M, Verburgt CM, Sovran B, et al. Nutritional therapy to modulate tryptophan metabolism and aryl hydrocarbon-receptor signaling activation in human diseases. Nutrients 2020;12:2846.
16. Mar JS, Ota N, Pokorzynski ND, et al. IL-22 alters gut microbiota composition and function to increase aryl hydrocarbon receptor activity in mice and humans. Microbiome 2023;11:47.
17. Zelante T, Iannitti RG, Cunha C, et al. Tryptophan catabolites from microbiota engage aryl hydrocarbon receptor and balance mucosal reactivity via interleukin-22. Immunity 2013;39:372–385.
18. Lamas B, Richard ML, Leducq V, et al. CARD9 impacts colitis by altering gut microbiota metabolism of tryptophan into aryl hydrocarbon receptor ligands. Nat Med 2016;22:598–605.
19. Seo SK, Kwon B. Immune regulation through tryptophan metabolism. Exp Mol Med 2023;55:1371–1379.
20. Jongen AC, Bosmans JW, Kartal S, et al. Predictive factors for anastomotic leakage after colorectal surgery: study protocol for a prospective observational study (REVEAL study). JMIR Res Protoc 2016;5:e90.
21. Bosmans J, Moossdorff M, Al-Taher M, et al. International consensus statement regarding the use of animal models for research on anastomoses in the lower gastrointestinal tract. Int J Colorectal Dis 2016;31:1021–1030.
22. Keir M, Yi Y, Lu T, Ghilardi N. The role of IL-22 in intestinal health and disease. J Exp Med 2020;217:e20192195.
23. Zenewicz LA. IL-22: there is a gap in our knowledge. Immunohorizons 2018;2:198–207.
24. Kiss EA, Vonarbourg C, Kopfmann S, et al. Natural aryl hydrocarbon receptor ligands control organogenesis of intestinal lymphoid follicles. Science 2011;334:1561–1565.
25. Perusina Lanfranca M, Lin Y, Fang J, et al. Biological and pathological activities of interleukin-22. J Mol Med (Berl) 2016;94:523–534.
26. Lamas B, Natividad JM, Sokol H. Aryl hydrocarbon receptor and intestinal immunity. Mucosal Immunol 2018;11:1024–1038.
27. Anderson DI, Keskey R, Ackerman MT, et al. Enterococcus faecalis is associated with anastomotic leak in patients undergoing colorectal surgery. Surg Infect (Larchmt) 2021;22:1047–1051.
28. Jacobson RA, Wienholts K, Williamson AJ, et al. Enterococcus faecalis exploits the human fibrinolytic system to drive excess collagenolysis: implications in gut healing and identification of druggable targets. Am J Physiol Gastrointest Liver Physiol 2020;318:G1–G9.
29. Suding P, Jensen E, Abramson MA, et al. Definitive risk factors for anastomotic leaks in elective open colorectal resection. Arch Surg 2008;143:907–911; discussion 11–2.
30. Arshad T, Mansur F, Palek R, et al. A double edged sword role of interleukin-22 in wound healing and tissue regeneration. Front Immunol 2020;11:2148.
31. Zindl CL, Lai JF, Lee YK, et al. IL-22-producing neutrophils contribute to antimicrobial defense and restitution of colonic epithelial integrity during colitis. Proc Natl Acad Sci U S A 2013;110:12768–12773.
32. Zha JM, Li HS, Lin Q, et al. Interleukin 22 expands transit-amplifying cells while depleting Lgr5(+) stem cells via inhibition of Wnt and notch signaling. Cell Mol Gastroenterol Hepatol 2019;7:255–274.

33. Coorens M, Rao A, Grafe SK, et al. Innate lymphoid cell type 3-derived interleukin-22 boosts lipocalin-2 production in intestinal epithelial cells via synergy between STAT3 and NF-kappaB. J Biol Chem 2019; 294:6027–6041.
34. Neurath MF, Travis SP. Mucosal healing in inflammatory bowel diseases: a systematic review. Gut 2012; 61:1619–1635.
35. Pickert G, Neufert C, Leppkes M, et al. STAT3 links IL-22 signaling in intestinal epithelial cells to mucosal wound healing. J Exp Med 2009;206:1465–1472.
36. Bevins CL, Salzman NH. Paneth cells, antimicrobial peptides and maintenance of intestinal homeostasis. Nat Rev Microbiol 2011;9:356–368.
37. Qiu J, Heller JJ, Guo XH, et al. The aryl hydrocarbon receptor regulates gut immunity through modulation of innate lymphoid cells. Immunity 2012;36:92–104.
38. Lee JS, Cella M, McDonald KG, Garlanda C, et al. AHR drives the development of gut ILC22 cells and postnatal lymphoid tissues via pathways dependent on and independent of Notch. Nat Immunol 2011;13:144–151.
39. Zenewicz LA, Yancopoulos GD, Valenzuela DM, et al. Innate and adaptive interleukin-22 protects mice from inflammatory bowel disease. Immunity 2008;29:947–957.
40. Sugimoto K, Ogawa A, Mizoguchi E, et al. IL-22 ameliorates intestinal inflammation in a mouse model of ulcerative colitis. J Clin Invest 2008;118:534–544.
41. Kang H, Chen Z, Wang B, Chen Z. The AhR/IL-22 axis in chronic gut inflammation: unraveling mechanisms and therapeutic prospects. Front Immunol 2025;16: 1668173.
42. Diny NL, Schonfeldova B, Shapiro M, et al. The aryl hydrocarbon receptor contributes to tissue adaptation of intestinal eosinophils in mice. J Exp Med 2022;219: e20210970.
43. Wiggins BG, Wang YF, Burke A, et al. Endothelial sensing of AHR ligands regulates intestinal homeostasis. Nature 2023;621:821–829.
44. Metidji A, Omenetti S, Crotta S, et al. The environmental sensor AHR protects from inflammatory damage by maintaining intestinal stem cell homeostasis and barrier integrity. Immunity 2019;50:1542.
45. Shah K, Maradana MR, Joaquina Delas M, et al. Cell-intrinsic Aryl Hydrocarbon Receptor signalling is required for the resolution of injury-induced colonic stem cells. Nat Commun 2022;13:1827.
46. Stockinger B, Shah K, Wincent E. AHR in the intestinal microenvironment: safeguarding barrier function. Nat Rev Gastroenterol Hepatol 2021;18:559–570.
47. Guenin-Mace L, Konieczny P, Naik S. Immune-epithelial cross talk in regeneration and repair. Annu Rev Immunol 2023;41:207–228.
48. Hyoju SK, Adriaansens C, Wienholts K, et al. Low-fat/high-fibre diet prehabilitation improves anastomotic healing via the microbiome: an experimental model. Br J Surg 2020;107:743–755.
49. Kok DE, Arron MNN, Huibregtse T, et al. Association of habitual preoperative dietary fiber intake with complications after colorectal cancer surgery. JAMA Surg 2021;156:1–10.
50. Yamamoto T, Hatabayashi K, Arita M, et al. Kynurenine signaling through the aryl hydrocarbon receptor maintains the undifferentiated state of human embryonic stem cells. Sci Signal 2019;12:eaaw3306.
51. Rueda GH, Causada-Calo N, Borojevic R, et al. Oral tryptophan activates duodenal aryl hydrocarbon receptor in healthy subjects: a crossover randomized controlled trial. Am J Physiol Gastrointest Liver Physiol 2024;326:G687–G696.
52. Natividad JM, Agus A, Planchais J, et al. Impaired aryl hydrocarbon receptor ligand production by the gut microbiota is a key factor in metabolic syndrome. Cell Metab 2018;28:737–749.e4.
53. Nikolaus S, Schulte B, Al-Massad N, et al. Increased tryptophan metabolism is associated with activity of inflammatory bowel diseases. Gastroenterology 2017; 153:1504–1516.e2.
54. Roager HM, Licht TR. Microbial tryptophan catabolites in health and disease. Nat Commun 2018;9:3294.
55. Ye X, Li H, Anjum K, et al. Dual role of indoles derived from intestinal microbiota on human health. Front Immunol 2022;13:903526.
56. Schroeder JC, Dinatale BC, Murray IA, et al. The uremic toxin 3-indoxyl sulfate is a potent endogenous agonist for the human aryl hydrocarbon receptor. Biochemistry 2010;49:393–400.
57. Sengul H, Bantavi V, Gloeck L, et al. Layer-specific molecular signatures of colon anastomotic healing and leakage in mice. Mol Med 2025;31:124.
58. van Helsdingen CPM, Wildeboer ACL, Zafeiropoulou K, et al. Histology and transcriptome insights into the early processes of intestinal anastomotic healing: a rat model. BJS Open 2023;7:zrad099.
59. Ternes D, Tsenkova M, Pozdeev VI, et al. Author correction: the gut microbial metabolite formate exacerbates colorectal cancer progression. Nat Metab 2023; 5:1638.
60. Lefèvre A, Mavel S, Nadal-Desbarats L, et al. Validation of a global quantitative analysis methodology of tryptophan metabolites in mice using LC-MS. Talanta 2019; 195:593–598.
61. Laurans L, Venteclef N, Haddad Y, et al. Genetic deficiency of indoleamine 2,3-dioxygenase promotes gut microbiota-mediated metabolic health. Nat Med 2018; 24:1113–1120.
62. Kozich JJ, Westcott SL, Baxter NT, et al. Development of a dual-index sequencing strategy and curation pipeline for analyzing amplicon sequence data on the MiSeq Illumina sequencing platform. Appl Environ Microbiol 2013;79:5112–5120.
63. Quast C, Pruesse E, Yilmaz P, et al. The SILVA ribosomal RNA gene database project: improved data processing and web-based tools. Nucleic Acids Res 2013; 41(Database issue):D590–D596.

Received December 12, 2024. Accepted February 10, 2026.

Correspondence
Address correspondence to: Sven Wehner, PhD, Department of Surgery, University Hospital Bonn, Venusberg-Campus 1, Bonn 53127, Germany. e-mail: sven.wehner@ukbonn.de.

Acknowledgments

The authors thank Heike Weighardt and Irmgard Förster (LIMES Institute, Bonn, Germany) and Brigitta Stockinger (Francis Crick Institute, London, United Kingdom) for providing the mouse lines (AhR$^{-/-}$ and VillinCreAhrfl/fl [(Tg(Vil1-cre))1000Gum/J]), respectively. The authors thank Artemiy Kovynev for validating independently the 16S RNA sequencing analysis. The authors thank all the members of the animal facility ARIA of the Amsterdam UMC, The Netherlands, Daisy I. Picavet (Department of Medical Biology, and Imaging Core Facility of Amsterdam UMC, The Netherlands), and Antoine Lefevre and Patrick Emond from Laboratoire de Médecine Nucléaire In Vitro, Centre Hospitalier Régionale Universitaire de Tours in Tours, France for the targeted tryptophan metabolomics. The authors also thank the Microscopy Core Facility of the University Hospital Bonn for providing help, services, and devices funded, among others, by the German Research Council (DFG, German Research Foundation; Project number 388159768), and the Flow Cytometry Core Facility (DFG Project number 216372401) of the University Hospital Bonn for supporting all FACS experiments. Finally, we thank Genentech, Inc, for providing neutralizing IL-22 antibody and IL-22Fc protein. The illustrative figures were created with BioRender.com.

The REVEAL study group includes: Joop L.M Konsten,[1] Jan Stoot,[2] and Nicole D. Bouvy,[3] from the [1]VieCuri Medisch Centrum, Venlo, Limburg, The Netherlands; [2]Zuyderland Medical Center, Limburg, The Netherlands; and [3]University Hospital Maastricht, Maastricht, The Netherlands.

CRediT Authorship Contributions

Vasiliki Bantavi (Data curation: Lead; Formal analysis: Lead; Investigation: Lead; Methodology: Lead; Software: Lead; Visualization: Lead; Writing – original draft: Lead; Writing – review & editing: Lead)

Laura Gloeck (Formal analysis: Supporting; Visualization: Supporting; Writing – review & editing: Supporting)

Patrick Leven (Visualization: Supporting; Writing – review & editing: Supporting)

Konstantina Zafeiropoulou (Formal analysis: Supporting; Writing – review & editing: Supporting)

Olaf Welting (Methodology: Supporting)

Hilal Sengul (Writing – review & editing: Supporting)

Patrik Efferz (Methodology: Supporting)

Bianca Schneiker (Methodology: Supporting)

Timothy Ryan McCulloch (Formal analysis: Supporting; Methodology: Supporting; Resources: Supporting)

Christoph Wilhelm (Methodology: Supporting; Resources: Supporting; Writing – review & editing: Supporting)

Sandra Blaess (Resources: Supporting; Supervision: Supporting)

Anneke Fuss (Methodology: Supporting)

Joep Derikx (Resources: Supporting; Writing – review & editing: Supporting)

Bruno Sovran (Conceptualization: Supporting; Supervision: Supporting; Writing – review & editing: Supporting)

Wouter de Jonge (Conceptualization: Lead; Funding acquisition: Lead; Project administration: Lead; Resources: Lead; Supervision: Lead; Writing – review & editing: Lead)

Sven Wehner (Conceptualization: Lead; Funding acquisition: Lead; Project administration: Lead; Resources: Lead; Supervision: Lead; Writing – review & editing: Lead)

Conflicts of interest

The authors disclose no conflicts.

Funding

Vasiliki Bantavi, Hilal Sengul, Wouter de Jonge, and Sven Wehner were funded by the international doctoral training network GROWTH as part of the Horizon 2020 Maria Skłodowska-Curie program (agreement No. 814168). Vasiliki Bantavi, Bruno Sovran, Patrick Leven, Wouter de Jonge, and Sven Wehner received funding from “Healthy Diet, Healthy Life (HDHL)” project “METARYL.” Joep Derikx is supported by a Vidi-stipendium (016.196.343) entitled ‘Unraveling the mechanisms of intestinal anastomotic healing and leakage’ awarded from the Netherlands Organization for Health Research and Development (ZonMw), and an Amsterdam UMC stipendium (200402) entitled ‘A nervous anastomosis’ awarded from Amsterdam University Medical Centers. Konstantina Zafeiropoulou was funded by an Amsterdam UMC stipendium (200402) entitled ‘A nervous anastomosis’ awarded from Amsterdam University Medical Centers, and a TKI Life Science and Health grant. Wouter de Jonge and Bruno Sovran were supported by a grant from Biostime Institute for Nutrition and Care. Christoph Wilhelm was supported by the Deutsche Forschungsgemeinschaft (DFG, German Research Foundation) under Germany’s Excellence Strategy – EXC2151 – 390873048 and the DFG program grant 470987585. Timothy Ryan McCulloch was supported by a DFG Walter Benjamin Fellowship 556527736. Anneke Fuss was supported by the iBehave project funded from the program ‘Netzwerke 2021’, an initiative of the Ministry of Culture and Science of the State of North Rhine-Westphalia. The funders had no role in the design of the study, in the collection, analyses, or interpretation of the data, in the writing of the manuscript, or in the decision to publish the results.

Data Availability

The authors declare that all data, analytical methods, and study materials will be available to other researchers through this publication or direct reasonable request to the corresponding author. This study includes no data deposited in external repositories.

Cellular and Molecular Gastroenterology and Hepatology, Volume *20*

Supplemental information

Tryptophan Indole Metabolites Reduce Anastomotic Leakage Through Aryl Hydrocarbon Receptor-Driven Interleukin-22 Production

Vasiliki Bantavi, Laura Gloeck, Patrick Leven, Konstantina Zafeiropoulou, Olaf Welting, Hilal Sengül, Patrik Efferz, Bianca Schneiker, Timothy Ryan McCulloch, Christoph Wilhelm, Sandra Blaess, Anneke Fuss, REVEAL study groupJoep P.M. Derikx, Bruno Sovran, Wouter J. de Jonge, and Sven Wehner

Supplementary Table 1. Demographics of the clinical cohort.

CRC patients					
**Age**	**Sex**	**Tumor location**	**Stage**	**Type of surgery**	**AH/AL**
63	M	Left colon	T1	Left hemicolectomy	AH
67	M	Right colon	T2	Right hemicolectomy	AH
51	F	Rectum	T3	LAR	AH
66	M	Left colon	T3	LAR	AH
60	M	Left colon	T1	Left hemicolectomy	AH
76	M	Rectum	T0	LAR	AH
69	M	Rectum	T3	Robot LAR	AH
56	M	Left colon	T4	Sigmoid resection	AH
73	M	Left colon	T3	Robot LAR	AH
59	F	Left colon	T3	Sigmoid resection	AH
71	F	Transverse colon	Unclear	Extended right hemicolectomy	AH
79	M	Rectum	T2	Sigmoid resection	AH
81	M	Right colon	T3	Right hemicolectomy	AH
53	F	Rectum	T4	Robot PME	AH
75	M	Right colon	T3	Right hemicolectomy	AH
70	M	Left colon	T0	Sigmoid resection	AH
66	M	Right colon	T3	Right hemicolectomy	AH
75	M	Right colon	T0	Right hemicolectomy	AH
47	M	Left colon	T3	Robot PME	AH
71	M	Left colon	T1	Sigmoid resection	AH
57	M	Rectum	T2	TATME	AH
67	M	Left colon	T1	Sigmoid resection	AH
58	M	Left colon	T4	Sigmoid resection	AH
69	M	Right colon	T3	Right hemicolectomy	AH
75	M	Rectum	T2	TATME	AH
74	M	Left colon	T3	Sigmoid resection	AH
78	F	Left colon	T3	Left hemicolectomy	AH
76	M	Rectum	T2	LAR	AH
71	M	Left colon	T2	Sigmoid resection	AH
75	M	Rectum	T1	LAR	AH
75	M	Left colon	T3	LAR	AH
56	M	Left colon	T3	Sigmoid resection	AH
62	M	Left colon	T3	Sigmoid resection	AH
75	M	Rectum	T3	LAR	AL
71	M	Right colon	T4	Right hemicolectomy	AL
72	M	Rectum	T2	LAR	AL
52	M	Rectum	T3	LAR	AL
65	M	Rectum	T1	LAR	AL
59	M	Rectum	T3	Robot LAR	AL
75	M	Rectum	T3	Robot LAR	AL
71	M	Left colon	T2	Sigmoid resection	AL
51	F	Left colon	T3	Sigmoid resection	AL
83	M	Right colon	T3	Right hemicolectomy	AL
77	M	Left colon	T3	Left hemicolectomy	AL
61	F	Rectum	T3	LAR	AL
76	M	Rectum	T2	LAR	AL
68	M	Transverse colon	T2	Transversectomy	AL
73	M	Left colon	T1	Sigmoid resection	AL
75	F	Right colon	T1	Right hemicolectomy	AL
75	M	Rectum	T3	Sigmoid resection with extended left hemicolectomy	AL
58	F	Right colon	T3	Right hemicolectomy	AL
61	M	Left colon	T3	LAR	AL

Tryptophan metabolites	AH (n=33)	AL (n=19)	*P-value*
Tryptophan	6162	6311	0.5849
Indole-3-acetic acid	454.0	210.9	0.0678
Indole-3-aldehyde	172.4	244.1	0.5592
Indole-3-lactic acid	37.47	51.67	0.0947
Indole-3-acetamide	1.43[19]	1.43[14]	0.4977
Indole-3-sulfate	0.85[33]	0.85[19]	>0.999
Tryptophol	9.460	16.76	0.0708
Tryptamine	16.64	17.39	0.2262
Indole	218.0	156.2	0.9400
Kynurenine	5.310[4]	3.880[3]	0.6820
Kynurenic acid	102.9	73.59	0.6649
Xanthurenic acid	16.93[9]	19.61[6]	0.7029
Picolinic acid	1900	2037	0.5217
Quinolinic acid	1.660[17]	1.660[12]	0.5339
3-OH-Kynurenine	0.75[33]	0.75[19]	>0.999
3-OH-Anthranilic acid	3.880[13]	7.520[8]	0.6258
Serotonin	110.7	126.8	0.8360
5-OH-Tryptophan	2.310	3.120	0.7956
5-OH-Indole acetic acid	8.180[12]	10.32[7]	0.7720
Melatonin	0.000[33]	0.000[19]	>0.999
N-acetyl-serotonin	5.730	5.260	0.6960
Indoles (sum)	812.8	778.3	0.6513
IDO pathway (sum)	2067	2069	0.5341
5-HT pathway (sum)	133.6	151.7	0.8803
IDO activity (Kyn/Trp ratio)	0.00072	0.00044	0.3257
IAA/Kyn ratio	92.99	44.29	0.2158
IAA/Trp ratio	0.1053	0.04749	0.0771
IAA/Sum IDO ratio	0.2422	0.099	0.0100

Supplementary Table 2. Preoperative fecal concentrations of tryptophan metabolites in patients with colorectal cancer that underwent anastomotic surgery. Values resemble median amounts within each group. Number of samples below the detection limit are shown in square brackets. Statistical significance was determined by Mann-Whitney U test or unpaired t test for AL and AH comparisons. $**P < .01$.

Tryptophan metabolites	AH (n=28)	AL (n=15)	*P-value*
Tryptophan	5710	5937	0.7149
**Indole-3-acetic acid**	470.9	173.2	**0.0198**
Indole-3-aldehyde	173.1	276.8	0.4415
**Indole-3-lactic acid**	33.83 [1]	54.08	**0.0474**
Indole-3-acetamide	1.43[16]	1.43[4]	0.5491
Indole-3-sulfate	0.85[28]	0.85[15]	>0.999
Tryptophol	9.550	16.76	0.0954
Tryptamine	9.810	18.33	0.1373
Indole	215.2	155.0	0.6776
Kynurenine	4.815[4]	3.200[3]	0.7716
Kynurenic acid	82.97	69.02	0.3434
Xanthurenic acid	13.46[7]	17.85[5]	0.9542
Picolinic acid	1890	1910	0.6054
Quinolinic acid	1.950[13]	1.660[9]	0.5838
3-OH-Kynurenine	0.75[28]	0.75[15]	>0.999
3-OH-Anthranilic acid	4.135[10]	8.060[5]	0.3072
Serotonin	136.4	69.24	0.3699
5-OH-Tryptophan	2.240	3.160	0.5076
5-OH-Indole acetic acid	6.829[11]	9.740[3]	0.9068
Melatonin	0.000[28]	0.000[15]	>0.999
N-acetyl-serotonin	6.140	6.360	0.8653
Indoles (sum)	829.1	630.0	0.4567
IDO pathway (sum)	1999	2013	0.6231
5-HT pathway (sum)	151.2	84.16	0.3977
IDO activity (Kyn/Trp ratio)	0.00070	0.000062	0.6769
IAA/Kyn ratio	104.1	48.62	0.1373
**IAA/Trp ratio**	0.1066	0.0317	**0.0368**
**IAA/Sum IDO ratio**	0.2602	0.07783	**0.0030**

Supplementary Table 3. Preoperative fecal concentrations of tryptophan metabolites in male patients with colorectal cancer that underwent anastomotic surgery. Values resemble median amounts within each group. Number of samples below the detection limit are shown in square brackets. Statistical significance was determined by Mann-Whitney U test or unpaired t test for AL and AH comparisons. $**P < .01$.

Tryptophan metabolites	AH (n=5)	AL (n=4)	*P-value*
Tryptophan	6314	10004	0.9048
Indole-3-acetic acid	426.0	779.1	0.4127
Indole-3-aldehyde	167.5	175.4	0.9048
Indole-3-lactic acid	37.47	32.35	0.9048
Indole-3-acetamide	1.43[3]	1.43[3]	>0.999
Indole-3-sulfate	0.85[5]	0.85[5]	>0.999
Tryptophol	8.570	17.31	0.5556
Tryptamine	26.87	8.625	0.7302
Indole	218.0	293	0.7302
Kynurenine	5.310	3.965	0.9048
Kynurenic acid	121.5	164.9	0.2857
Xanthurenic acid	25.82[2]	51.97[1]	0.2778
Picolinic acid	2441	2584	0.7302
Quinolinic acid	1.660[4]	1.660[3]	>0.999
3-OH-Kynurenine	0.75[5]	0.75[4]	>0.999
3-OH-Anthranilic acid	1.550[3]	1.550[3]	0.5635
Serotonin	86.34	173.6	0.2857
5-OH-Tryptophan	2.710	2.035	0.5556
5-OH-Indole acetic acid	13.86[1]	18.0[1]	0.7937
Melatonin	0.000[5]	0.000[4]	>0.999
N-acetyl-serotonin	3.030	4.530	0.3175
Indoles (sum)	683.7	1337	0.5556
IDO pathway (sum)	2479	2808	0.7302
5-HT pathway (sum)	91.28	199.6	0.1111
IDO activity (Kyn/Trp ratio)	0.00089	0.00041	0.2857
IAA/Kyn ratio	190.1	263.8	0.5556
IAA/Trp ratio	0.02303	0.1152	0.9048
IAA/Sum IDO ratio	0.1366	0.2109	0.9048

Supplementary Table 4. Preoperative fecal concentrations of tryptophan metabolites in female patients with colorectal cancer that underwent anastomotic surgery. Values resemble median amounts within each group. Number of samples below the detection limit are shown in square brackets. Statistical significance was determined by Mann-Whitney U test for AL and AH comparisons.

	Anastomotic complication score (ACS)
AH	0 No adhesions or abnormalities
	1 Adhesion to fat pad, clean anastomosis underneath
	2 Adhesion to intestinal loop, abdominal wall or other organ
AL	3 Anastomotic defect found underneath adhesion, no other abnormalities
	4 Signs of possible contamination (e.g., small abscesses)
	5 Clear anastomotic complication; spread of pus, obstruction at anastomosis, sign of peritonitis
	6 Fecal peritonitis / Death due to peritonitis

Supplementary Table 5. ACS scoring system.

Tryptophan metabolites	Low (n=12)	High (n=8)	*P-value*
Tryptophan	71.16	4379	<0.0001
Indole-3-acetic acid	5.685	34.56	0.0002
Indole-3-aldehyde	6.375	25.80	<0.0001
Indole-3-lactic acid	5.640[1]	25.72	0.0124
Indole-3-acetamide	0.5300	0.8485	0.0001
Indole-3-sulfate	2.500[11]	2.500[5]	0.2206
Tryptophol	2.68	23.22	0.0001
Tryptamine	0.4500[12]	0.4500[8]	>0.999
Indole	26.195	97.025	0.0024
Kynurenine	11.38	60.01	0.0012
Kynurenic acid	7.685	18.42	0.0473
Xanthurenic acid	13.185	50.28	0.0095
Picolinic acid	747.0	725.0	0.6784
Quinolinic acid	46.335	675.49	<0.0001
3-OH-Kynurenine	0.135[12]	0.5455[1]	0.0006
3-OH-Anthranilic acid	1.045	4.170	0.0001
Serotonin	30.875	85.03	0.0001
5-OH-Tryptophan	3.615	13.19	0.0005
5-OH-Indole acetic acid	71.16	837.2	0.0387
Melatonin	0.015[7]	0.056[2]	0.0312
N-acetyl-serotonin	0.416	0.863	0.0238
Indoles (sum)	20.34	122.9	0.0001
IDO pathway (sum)	822.685	1685.61	0.0003
5-HT pathway (sum)	113.131	917.03	0.0062
IDO activity (Kyn/Trp)	0.01943	0.01323	0.0691

Supplementary Table 6. Fecal tryptophan metabolite concentrations after low- and high-tryptophan diets and before anastomotic surgery. Values resemble median amounts within each group. Number of samples below the detection limit are shown in square brackets. Statistical significance was determined by Mann-Whitney U test. *P < .05, **P < .01, ***P < .001, ****P < A S.0001.

Supplementary Table 7. Composition of low- and high-tryptophan diets.

**Ingredients**	Product No.	**0.1 % Trp AA diet** S0016-E080	**1 % Trp AA diet** S0016-E084
Corn starch	%	30,000	30,000
Maltodextrin	%	16,000	16,000
Sucrose	%	18,077	17,170
Cellulose	%	5,000	5,000
L-Lysine HCl	%	1,800	1,800
DL-Methionine	%	0,700	0,700
L-Cystine	%	0,350	0,350
L-Threonine	%	0,900	0,900
L-Tryptophan	%	0,103	1,010
L-Arginine	%	1,000	1,000
L-Histidine	%	0,500	0,500
L-Valine	%	0,900	0,900
L-Isoleucine	%	0,820	0,820
L-Leucine	%	1,200	1,200
L-Phenylalanine	%	0,750	0,750
L-Tyrosine	%	0,500	0,500
Glycine	%	2,500	2,500
L-Glutamic acid	%	3,000	3,000
L-Glutamine	%	1,000	1,000
L-Aspartic acid	%	0,350	0,350
L-Asparagine	%	0,600	0,600
L-Proline	%	0,400	0,400
L-Alanine	%	0,400	0,400
L-Serine	%	0,350	0,350
Vitamin premix	%	1,000	1,000
Minerals	%	4,500	4,500
Choline Cl	%	0,250	0,250
Butylated hydroxytoluene	%	0,010	0,010
Food dye	%	0,040	0,040
Soybean oil	%	7,000	7,000
Proximate contents			
Crude protein (N x 6.25)	%	16,4	17,2
Crude fat	%	7,0	7,0
Crude fiber	%	5,0	5,0
Crude ash	%	3,4	3,4
Starch	%	28,9	28,9
Sugar	%	19,3	18,4
Dextrins	%	15,8	15,8
Lysine	%	1,40	1,40
Methionine	%	0,69	0,69
**Ingredients**	Product No.	**0.1 % Trp AA diet** S0016-E080	**1 % Trp AA diet** S0016-E084
Cystine	%	0,35	0,35
Met+ Cys	%	1,03	1,03
Threonine	%	0,88	0,88
Tryptophan	%	0,10	1,00
Energy (Atwater)	MJ/kg	16,7	16,7
Protein	kJ%	16	17
Fat	kJ%	16	16
Carbohydrates	kJ%	68	67

These diets are a modification of V1534-300

Supplementary Table 8. Differential abundance of ASVs (DESeq2 *P*-adj < .05).

	**log2FoldChange**	**padj**
ASVX_226_Clostridiales_vadinBB60_group	-6.830659375	6.88E-06
ASVX_224_Lachnospiraceae	-6.517952821	9.35E-06
ASVX_339_Lachnospiraceae	-5.330281352	0.0007836
ASVX_236_Clostridiales_vadinBB60_group	-5.265350626	0.00046453
ASVX_209_Lachnospiraceae	-4.610986513	0.00013535
SVX_273_Lachnospiraceae_NK4A136_group_N	-4.588031293	0.00214944
ASVX_278_Alistipes_NA	-4.313172975	0.00536893
ASVX_684_Desulfovibrionaceae	-4.241600909	0.00536893
ASVX_227_Lachnospiraceae	-4.229238314	0.00757519
ASVX_698_Faecalibaculum_NA	-4.216525926	0.00536893
ASVX_122_Lachnospiraceae	-4.075905095	0.00881748
ASVX_270_Muribaculaceae	-3.9570562	0.01087137
ASVX_121_Oscillibacter_NA	-3.890236064	0.00536893
ASVX_225_Lachnospiraceae	-3.741763898	0.01135497
ASVX_554_Lachnospiraceae	-3.340463185	0.0193342
ASVX_676_Lachnoclostridium_NA	-3.309262705	0.03640394
ASVX_181_Ruminiclostridium_9_NA	-3.270845237	0.02853696
SVX_347_Lachnospiraceae_NK4A136_group_N	-3.132495481	0.04735964
ASVX_157_Muribaculaceae	-3.011278664	0.03877034
ASVX_654_Lachnospiraceae	-3.005747703	0.03877034
ASVX_64_Anaerotruncus_colihominis	-2.999836583	0.01380567
ASVX_158_Lachnospiraceae	-2.990286734	0.02447756
ASVX_394_Muribaculaceae	-2.976956639	0.04685539
ASVX_545_Lachnospiraceae	-2.906211901	0.02853696
ASVX_993_Akkermansia_NA	-2.863992899	0.04978538
ASVX_92_Lachnospiraceae_UCG-006_NA	-2.738845325	0.03557737
ASVX_212_Muribaculaceae	2.151241055	0.0459244
ASVX_440_Ruminococcaceae_UCG-014_NA	2.32488226	0.04978538
ASVX_845_Ruminiclostridium_9_NA	2.324916017	0.03877034
ASVX_945_Lachnoclostridium_NA	2.492999423	0.04182268
ASVX_162_Turicibacter_NA	2.536135834	0.04982823
SVX_737_Lachnospiraceae_NK4A136_group_N	2.611353433	0.035856
ASVX_691_Lachnospiraceae	2.693928727	0.03135669
ASVX_582_GCA-900066575_NA	2.709177966	0.02884301
ASVX_564_Parasutterella_NA	2.815020094	0.04224675
SVX_504_Lachnospiraceae_NK4A136_group_N	2.925287207	0.02924428
ASVX_346_Clostridiales_vadinBB60_group	2.976875285	0.00984503
ASVX_535_Ruminiclostridium_NA	3.014435434	0.01270784
ASVX_565_Ruminiclostridium_9_NA	3.05703631	0.00990535
ASVX_280_Lachnospiraceae	3.072218315	0.00990535
ASVX_567_Bacteroides_NA	3.09445938	0.01489324
ASVX_551_Flavonifractor_NA	3.100700942	0.01636591
ASVX_522_Intestinimonas_NA	3.206364591	0.00984503
SVX_232_Lachnospiraceae_NK4A136_group_N	3.285813271	0.01730885
SVX_492_Lachnospiraceae_NK4A136_group_N	3.41066325	0.00933085
ASVX_254_Parasutterella_NA	3.442143263	0.0007836
ASVX_599_Ruminococcaceae_UCG-014_NA	3.49380836	0.00790046

	log2FoldChange	padj
ASVX_207_Anaerotruncus_NA	3.574508393	0.00547841
ASVX_399_Clostridiales_vadinBB60_group	4.369898664	0.00081166
SVX_471_Lachnospiraceae_NK4A136_group_N	4.546416478	0.00081166
ASVX_319_Pseudomonas_NA	5.139069309	0.0005343
ASVX_450_Ruminococcaceae_UCG-014_NA	6.139239776	4.04E-06

Supplementary Table 9. qPCR primers used for gene expression analysis.

Gene	Forward Sequence (5'-3')	Reverse Sequence (5'-3')
AhR	TCTAAGCGACACAGAGACCG	GGTGGACTTTAATGCAACATCA
Cyp1a1	CCTCATGTACCTGGTAACCA	AAGGATGAATGCCGGAAGGT
IL-22	CATGCAGGAGGTGGTACCTT	CAGACGCAAGCATTTCTCAG
IL-17A	TTTAACTCCCTTGGCGCAAAA	CTTTCCCTCCGCATTGACAC
IL-22RA1	GGAGATAAGTTGCTTCCCCAAA	CATCCACGCCCAGCCATAG
IL-23	AGCGGGACATATGAATCTACTAAGAGA	GTCCTAGTAGGGAGGTGTGAAGTTG
IL-10	AGAAGCATGGCCCAGAAATCA	GGCCTTGTAGACACCTTGGT
Reg3γ	TTCCTGTCCTCCATGATCAAAA	CATCCACCTCTGTTGGGTTCA
Reg3β	ATGCCGAAGACGATCAGTGTG	ACCAAACTTCCCTCAAACCAAT
IL-6	AAGTCGGAGGCTTAATTACACATGT	CCATTGCACAACTCTTTTCTCATT
IL-1β	TTGACGGACCCCAAAAGATG	CAGGACAGCCCAGGTCAAAG
S100a8	ATGCCGTCTGAACTGGAGAA	CGATATTTATATTCTGCACAAACTGAG
SOCS3	CCACCTGGACTCCTATGAGAAAGT	CCTCTGACCCTTTTGCTCCTTA
GAPDH	TaqMan™ probe NM_008084.2 (Applied Biosystems)	
